# Modulation of the endoplasmic reticulum stress and unfolded protein response mitigates the behavioral effects of early-life stress

**DOI:** 10.1007/s43440-023-00456-6

**Published:** 2023-02-27

**Authors:** Anna Solarz-Andrzejewska, Iwona Majcher-Maślanka, Joanna Kryst, Agnieszka Chocyk

**Affiliations:** 1 Laboratory of Pharmacology and Brain Biostructure, Department of Pharmacology, Maj Institute of Pharmacology, Polish Academy of Sciences, Smętna Street 12, 31-343 Kraków, Poland; 2 Department of Chemistry and Biochemistry, Institute for Basics Sciences, Faculty of Physiotherapy, University of Physical Education, Jana Pawła II Av. 78, 31-571 Kraków, Poland

**Keywords:** Endoplasmic reticulum stress, Unfolded protein response, Maternal separation, Apoptosis, Salubrinal, Medial prefrontal cortex

## Abstract

**Background:**

Early-life stress (ELS) affects brain development and increases the risk of mental disorders associated with the dysfunction of the medial prefrontal cortex (mPFC). The mechanisms of ELS action are not well understood. Endoplasmic reticulum (ER) stress and the unfolded protein response (UPR) are cellular processes involved in brain maturation through the regulation of pro-survival or proapoptotic processes. We hypothesized that ER stress and the UPR in the mPFC are involved in the neurobiology of ELS.

**Methods:**

We performed a maternal separation (MS) procedure from postnatal days 1 to 14 in rats. Before each MS, pups were injected with an inhibitor of ER stress, salubrinal or a vehicle. The mRNA and protein expression of UPR and apoptotic markers were evaluated in the mPFC using RT-qPCR and Western blot methods, respectively. We also estimated the numbers of neurons and glial cells using stereological methods. Additionally, we assessed behavioral phenotypes related to fear, anhedonia and response to psychostimulants.

**Results:**

MS slightly enhanced the activation of the UPR in juveniles and modulated the expression of apoptotic markers in juveniles and preadolescents but not in adults. Additionally, MS did not affect the numbers of neurons and glial cells at any age. Both salubrinal and vehicle blunted the expression of UPR markers in juvenile and preadolescent MS rats, often in a treatment-specific manner. Moreover, salubrinal and vehicle generally alleviated the behavioral effects of MS in preadolescent and adult rats.

**Conclusions:**

Modulation of ER stress and UPR processes may potentially underlie susceptibility or resilience to ELS.

**Supplementary Information:**

The online version contains supplementary material available at 10.1007/s43440-023-00456-6.

## Introduction

Clinical and epidemiological studies have clearly indicated that early-life stress (ELS) increases the risk of mental health problems, such as mood and anxiety disorders, substance use disorder and cognitive deficits. Moreover, ELS accelerates the early onset of the abovementioned mental disorders in children and adolescents [1]. Thanks to animal studies and advances in neuroimaging techniques in humans, it is evident that ELS interferes with brain development [2, 3]. Although psychobiological consequences of ELS have been extensively explored in the past decade (for review see: [3–5]), the specific mechanisms of ELS action on brain development and maturation are still poorly understood and, thus, require intensive study.

One of the widely used and popular model of ELS and human psychopathology with a high construct validity is the repeated maternal separation procedure (MS) in rodents during the first two weeks of life [6, 7]. Our previous studies based on such MS procedure in rats have shown that this early-life experience affects the process of neurodevelopmental apoptosis in the midbrain and medial prefrontal cortex (mPFC) in males [8–10]. Specifically, we observed a sustained increase in the survival of midbrain neurons and a specific delay in neuronal apoptosis during adolescence in the mPFC, manifested as an increase in the number of neuronal cells, which could potentially affect proper neuronal network building and functioning [8, 10].

One of the key cellular processes affecting the decision of cells to survive or die in response to different environmental insults is endoplasmic reticulum (ER) stress and, closely related to it, the unfolded protein response (UPR) [11]. The ER governs the synthesis, folding, modification and transport of over one-third of cellular proteins and, thus, plays a central role in maintaining protein homeostasis (proteostasis). Many conditions, such as nutrient deprivation, hypoxia, loss of redox and calcium balance and increased protein load, may disturb proteostasis and lead to accumulation of unfolded or misfolded proteins and, consequently, to the induction of ER stress and the UPR [11, 12]. These processes, in general protective and adaptive, act to restore ER homeostasis by attenuating general translation, inducing chaperones and eliminating misfolded proteins. However, if cellular stress exceeds the pro-survival capability of the UPR, the ER induces cell death pathways through the proapoptotic component of the UPR [13, 14].

ER stress is sensed by three ER transmembrane proteins, i.e., inositol-requiring enzyme 1 (IRE1α), RNA-activated protein kinase (PKR)-like endoplasmic reticulum kinase (PERK) and activating transcription factor 6 (ATF6). Under resting conditions, these UPR sensors are bound to heat shock 70 kDa protein A 5 (HSPA5), also known as glucose-regulated protein 78 (GRP78). Upon ER stress and accumulation of unfolded or misfolded proteins, HSPA5 dissociates from these sensor molecules and allows their activation. During activation, IRE1α, also known as endoplasmic reticulum to nucleus signaling 1 and encoded by the Ern1 gene, undergoes autophosphorylation at serine 724 and then induces cleavage of X box-binding protein 1 (XBP1) mRNA and production of spliced XBP1 mRNA and protein. The spliced XBP1 protein is a highly active transcription factor that regulates genes involved in the UPR. Release of HSPA5 from ATF6 induces its translocation to the Golgi apparatus, where it is cleaved (activated). Active forms of ATF6 migrate into the nucleus, where they act as transcription factors that are also engaged in the regulation of UPR-related genes. The third sensor, PERK, also known as eukaryotic translation initiation factor 2 alpha kinase 3 and encoded by the Eif2ak3 gene, undergoes autophosphorylation at threonine 980 during its activation. Next, PERK phosphorylates (at serine 51), and in this way inactivates eukaryotic translation initiation factor 2α (eIF2α, encoded by the Eif2a gene), which causes an inhibition of general protein synthesis. Concurrently, some specific mRNA translation is allowed to further regulate UPR processes [11, 14, 15].

ER stress and UPR processes have been implicated in the pathophysiology of numerous diseases, such as cancer, diabetes, atherosclerosis and autoimmune and neurodegenerative diseases [15, 16]. However, in recent years, there has been an accumulation of data showing the involvement of ER stress and the UPR in the mechanisms of mental disorders [17–22]. Increased expression levels of UPR-related genes or proteins have been observed in the mPFC and temporal cortex of subjects with major depressive disorder (MDD) who died from suicide and in leukocytes from patients with MDD and posttraumatic stress disorder [20, 21, 23]. In contrast, an impaired ER stress response was observed in leukocytes from patients with bipolar disorder (BD) [24–26]. Additionally, functional polymorphisms in the promoter regions of XBP1 and HSPA5 were shown to have a possible association with BD in a Japanese population [17, 25]. Enhanced ER stress and activation of the UPR have also been observed in animal models of depression based on chronic restraint and chronic social defeat stress paradigms [27–29]. Surprisingly, although ELS is considered a relevant factor in the etiology of mental and neurodegenerative disorders, ER stress and UPR processes in the brain have not been well studied in animal models of ELS. We have recently shown that the repeated MS procedure in rats produces long-lasting upregulation of HSPA5 and another chaperone belonging to the 70-kDa heat shock protein family, HSPA1B, in the brain and blood, which suggests that ELS may influence ER stress and UPR processes throughout development [30]. Dynamic regulation of the UPR has been implicated in cell fate acquisition, cortical neurogenesis, cell maturation, apoptosis and neuritogenesis during prenatal development of the central nervous system [31]. However, the role of ER stress and UPR processes in postnatal brain maturation is poorly explored. Therefore, in the present study, we investigated whether MS enhances ER stress and the UPR and consequently affects cell fate, particularly the pro-survival or apoptotic processes during postnatal maturation of the mPFC. We focused on this specific brain region because the mPFC shows a prolonged developmental trajectory, characterized by waves of postnatal neurodevelopmental apoptosis and intensive structural and functional reorganization during preadolescence and adolescence periods, which makes it especially vulnerable to the effects of stress [32–35]. Moreover, the mPFC is highly implicated in the pathophysiology of mood and anxiety disorders [36]. Simultaneously, to further evaluate the potential role of ER stress and the UPR in the mechanisms of ELS, we also studied the short- and long-term effects of transient early-life inhibition of ER stress processes by salubrinal (SAL), a selective inhibitor of eIF2α dephosphorylation, on MS-induced biochemical changes and behavioral phenotypes related to anxiety, fear memory, anhedonia and response to psychostimulants.

## Materials and methods

### Animals

All experimental procedures were approved by the Local Ethics Committees for Animal Research in Krakow, Poland (permit no. 186/2018 issued 07/06/2018 and 136/2019 issued 27/06/2019) and met the requirements of the Directive 2010/63/EU of the European Parliament and of the Council of 22 September 2010 on the protection of animals used for scientific purposes. All efforts were made to minimize animal suffering.

Adult male and female Wistar rats were purchased from Charles River Laboratories (Sulzfeld, Germany). All animals were housed under controlled conditions with an artificial 12-h light/dark cycle (lights on from 07:00 to 19:00), 55% ± 10 humidity, and a temperature of 22 ºC ± 2. Food and tap water were freely available. The rats were mated at the Maj Institute of Pharmacology, PAS, Krakow Animal Facility. The offspring of primiparous dams were used in this study. Before delivery, the dams were housed individually in standard plastic cages (38 × 24 × 19 cm). The day of birth was designated as postnatal day (PND) 0. On PND 1, the litter size was standardized to eight pups per litter (four males and four females), and the litters were randomly assigned to one of the following early-life treatment: animal facility rearing (AFR), i.e., control condition, MS procedure, MS with salubrinal injections (SAL-MS) or MS with vehicle injections (VEH-MS).

### Repeated maternal separation and salubrinal injections

The MS procedure was performed as described previously by Solarz et al. and Chocyk et al. [8, 9, 30, 37–41]. Briefly, on PNDs 1–14, the dams and pups were removed from the maternity cages for 3 h (09.00–12.00) daily. The mothers were placed individually in the holding cages (38 × 24 × 19 cm), while each litter was placed in a plastic container (22 × 16 × 10 cm) lined with fresh bedding material, and the containers were moved to an adjacent room and placed in an incubator that was set at a constant temperature of 34 °C mimicking the nest temperature (a basic MS group). Additionally, before each daily separation, the pups from SAL-MS group received a single injection of salubrinal (1 mg/kg/5 ml *sc*, PNDs 1–14, Tocris), whereas VEH-MS group was injected with respective vehicle, i.e., 2.5% dimethyl sulfoxide (DMSO) in PBS (5 ml/kg, *sc*, PNDs 1–14, Sigma). SAL dose was chosen based on the data from literature which showed that SAL in a dose of 1 mg/kg can modulate ER stress processes in the brain and promotes neuroprotection [42, 43]. Moreover, such dose of SAL in repeated injections has been also safely administered to juvenile mice [44].

**Fig. 1:**
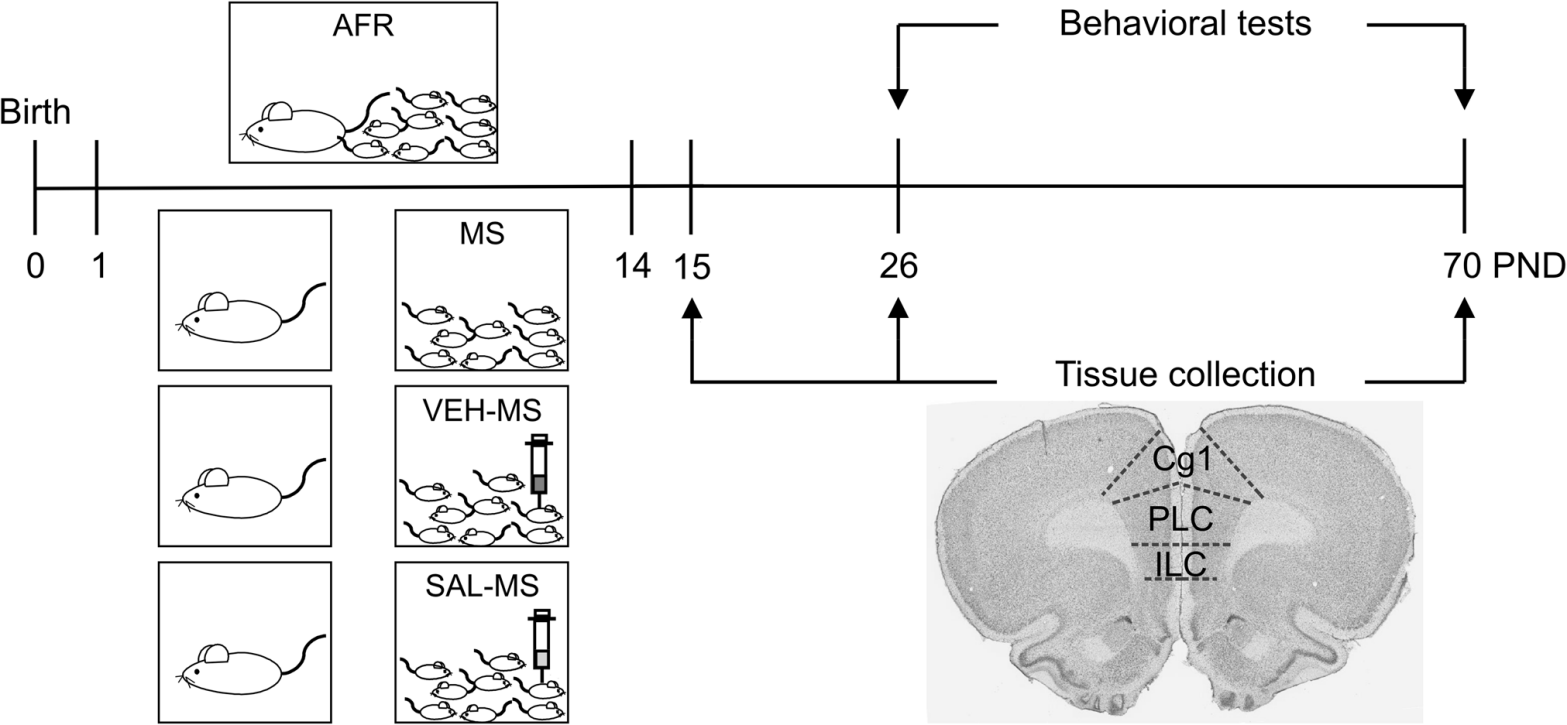
Scheme of the experimental paradigm applied in the study. On PNDs 1–14, the dams and pups were separated for 3 h daily. Before each daily separation, the pups from SAL-MS group received a single injection of salubrinal (SAL, 1 mg/kg *sc*), whereas VEH-MS group was injected with respective vehicle (2.5% DMSO in PBS, 5 ml/kg *sc*). On PND 26 and PND 70, behavioral phenotype of rats was assessed in the battery of tests, such as, the light/dark exploration, fear conditioning, sucrose preference and novelty- and amphetamine-induced locomotor activity. On PNDs 15, 26 and 70, the mPFC samples were also collected for biochemical analyses. Included photomicrograph shows a cresyl violet-stained rat brain section with marked subregions of the mPFC. *AFR* animal facility rearing, *Cg1* cingulate cortex 1, *ILC* infralimbic cortex, *MS* maternal separation, *mPFC* medial prefrontal cortex, *PLC* prelimbic cortex, *PND* postnatal day, *SAL* salubrinal, *VEH* vehicle

After the 3-h separation, the pups and dams were returned to the maternity cages. The AFR animals were left undisturbed with their mothers except during the weekly cage cleaning corresponding to a small amount of handling. Twenty-four hours after the last MS, i.e., on PND 15, a part of the animals was assigned to the experimental groups to investigate the effects of repeated MS and SAL in juveniles (Fig. 1). The rest of the animals were weaned on PND 22, sexed, and randomly distributed between subsequent experimental groups to investigate the long-term effects of repeated MS and SAL. These rats were housed under the controlled conditions (as described above) in standard plastic cages (57 × 33 × 20 cm) in the same-sex groups of five unrelated subjects according to the same treatment protocol until the preadolescence period (PND 26) or adulthood (PND 70) (Fig. 1). An exception was made in the case of sucrose preference test, when animals were singly housed in plastic cages (38 × 24 × 19 cm) from PND 22 to PND 26. The body weights of animals were measured daily from PND 1 to PND 15 and on PND 26 and PND 70 with a Kerm PCB electronic precision scale (Balingen, Germany).

### Experimental groups

A total number of 291 male rats were used in the study: 80 AFR, 68 MS, 71 VEH-MS and 72 SAL-MS rats. Female offspring was used in other scientific projects. In the case of biochemical experiments, the final experimental groups included the animals that originated from different litters and were unrelated (*n* = 5–6). In the case of behavioral experiments, maximum two subjects from the same litter were used (*n* = 9–15). The separate groups of animals were analyzed for (1) gene expression (on PND 15 and PND 26), (2) protein expression (on PND 15 and PND 26), and (3) different behavioral test (part of animals from that cohort was also used for immunohistochemistry and gene expression on PND 70).

### RT-qPCR

The RT-qPCR procedure was performed as described previously by Solarz et al. [30, 40, 41]. Briefly, on PND 15, PND 26, or PND70, the animals were sacrificed by decapitation (6 rats in each treatment and age group) and the brain was immediately removed from the skull. The mPFC (including the cingulate cortex 1 (Cg1), prelimbic cortex (PLC), and infralimbic cortex (ILC) regions) was dissected from 1 mm thick coronal sections using a rodent brain matrix (Ted Pella Inc., CA, USA). After dissection, the brain tissue was quickly frozen in liquid nitrogen and stored at − 80 ºC for later use. Total RNA from the brain tissue was extracted using the RNAeasy Mini Kit (Qiagen). The total RNA concentration was measured using an Eon absorbance microplate reader and Gen 5 software (BioTek, Winooski, VT, USA). RNA was reverse transcribed using a High-Capacity cDNA Reverse Transcription Kit (Thermo Fisher Scientific, MA). Quantitative real-time PCR was performed in duplicate with TaqMan® Gene Expression Assays (Thermo Fisher Scientific, MA; Table 1) using TaqMan™ Universal Master Mix II, no UNG (Thermo Fisher Scientific, MA) and the QuantStudio 12 K Flex System (Thermo Fisher Scientific, MA). Real-time PCR was conducted under the following conditions: 50 °C for 2 min and 95 °C for 10 min followed by 40 cycles of 95 °C for 15 s and 60 °C for 1 min. The abundance of RNA was calculated according to the following equation: abundance = 2^−(threshold cycle)^ [45]. The results were normalized to glyceraldehyde-3-phosphate dehydrogenase (Gapdh*)* expression levels.

**Table 1:** The list of TaqMan® Gene Expression Assays used in the study

Gene product	Gene name	Assay ID
Heat shock protein family A member 5 alias: glucose-regulated protein GRP 78kD	Hspa5	Rn01435769_g1
Endoplasmic reticulum to nucleus signaling 1 alias: inositol-requiring enzyme 1(IRE1α)	Ern1	Rn01471008_m1
Eukaryotic translation initiation factor 2 α kinase 3 alias: RNA-activated protein kinase (PRK)-like endoplasmic reticulum kinase (PERK)	Eif2ak3	Rn00581002_m1
Activating transcription factor 6	Atf6	Rn01490844_m1
Eukaryotic initiation factor 2	Eif2a	Rn01494813_m1
Caspase-12	Casp12	Rn00590440_m1
Caspase-9	Casp9	Rn00581212_m1
Caspase-3	Casp3	Rn00563902_m1
Apoptosis regulator Bcl2	Bcl2	Rn99999125_m1
Apoptosis regulator Bax	Bax	Rn01480161_g1
Glyceraldehyde-3-phosphate dehydrogenase	Gapdh	Rn99999916_s1

### Western blot

Western blot was performed as described previously by Majcher-Maślanka et al. [46, 47] and Solarz et al. [30]. Briefly, on PND 15 or PND 26, the animals were sacrificed by decapitation (6 rats in each treatment and age group), and the brain was rapidly removed from the skull. The mPFC (including Cg1, PLC and ILC regions) was dissected from 1 mm thick coronal sections using a rodent brain matrix (Ted Pella, CA, USA). After dissection, the brain tissue was quickly frozen in liquid nitrogen and stored at − 80 ºC until further use. The tissue was homogenized (TissueLyser, Retsch, Germany) in ice-cold lysis buffer (PathScan® Sandwich ELISA Lysis Buffer, Cell Signaling). The homogenates were then centrifuged for 15 min at 15,000 g at 4 ºC. The total protein concentration of the supernatants was determined using the bicinchoninic acid (BCA) method (Sigma-Aldrich, USA). Samples with equal protein concentrations were loaded into each lane, run on 10% sodium dodecyl sulfate–polyacrylamide gels in a Laemmli buffer system, and transferred onto a nitrocellulose membrane (Bio-Rad, USA). The blots were probed with diluted primary antibodies listed in Table 2. Then, the blots were incubated with the appropriate (anti-mouse IgG or anti-rabbit IgG) horseradish peroxidase-conjugated secondary antibodies (Roche Diagnostics, Basel, Switzerland), and the bands were visualized by enhanced chemiluminescence (Lumi-LightPlus Western Blotting Kit, Roche Diagnostics, Switzerland). The immunoblots were evaluated using a luminescent image analyzer (LAS-4000, Fujifilm, USA). Immunoblot images were acquired using exposure time increment option. Representative, unprocessed images were presented in Online Resource ESM_11-14. The relative levels of immunoreactivity were quantified using the Image Gauge software (Fujifilm, USA). The ratio of the specific protein level to the actin level was calculated for each sample to normalize for small variations in loading and transfer.

**Table 2:** List of primary antibodies used in the study

Specificity	Host	Dilution	Supplier	Product #
GRP78/HSPA5	Rabbit	1:1000	Cell Signaling Tech	3183
IRE1α	Rabbit	1:1000	Abcam	ab37073
p-IRE1α (S724)	Rabbit	1:1000	Abcam	ab48187
PERK	Rabbit	1:1000	Cell Signaling Tech	3192
p-PERK (Thr980)	Rabbit	1:1000	Cell Signaling Tech	3179
ATF6	Rabbit	1:1000	Proteintech	24169–1-AP
eIF2α	Rabbit	1:2000	Cell Signaling Tech	5324
p-eIF2α (S51)	Rabbit	1:1000	Cell Signaling Tech	3398
Bax	Mouse	1:500	Santa Cruz Biotech	sc-7480
Bcl2	Mouse	1:500	Santa Cruz Biotech	sc-7382
Caspase-3 (cleaved)	Rabbit	1:500	Cell Signaling Tech	9661
Caspase-9	Mouse	1:500	Cell Signaling Tech	9508
Caspase-12	Rabbit	1:500	Abcam	ab62484
NeuN	Mouse	1:1000	Millipore	MAB377
GFAP	Goat	1:250	Santa Cruz Biotech	sc-6170
IBA1	Rabbit	1:500	Proteintech	10904–1-AP

### Immunohistochemistry

Immunohistochemistry was performed as previously described by Chocyk et al. [8, 39] and Majcher-Maślanka et al. [10]. Briefly, on PND 26 and PND 70, the animals (6 rats in each treatment and age group) were deeply anesthetized and transcardially perfused with saline followed by 4% paraformaldehyde in 0.1 M PBS (pH 7.4). The brains were removed from the skulls, postfixed in 4% paraformaldehyde in PBS for 24 h at 4 °C, and sectioned using a vibratome (VT1000S, Leica, Wetzlar, Germany) into 50-μm thick coronal slices at the level of the mPFC (Bregma = 3.70 to 2.20 mm) according to a stereotaxic atlas of the rat brain [48]. Every fourth section was preserved for further processing (10–11 sections from each subject).

Free-floating sections were washed in 0.01 M PBS (pH 7.4) and incubated for 30 min in PBS containing 0.3% H_2_O_2_ and 0.2% Triton X-100. The sections were then rinsed and transferred to a blocking buffer (5% solution of appropriate normal serum (goat, horse or rabbit) in 0.2% Triton X-100 in PBS) for 1 h. After the blocking procedure, the sections were incubated for 48 h at 4 °C with a mouse anti-NeuN antibody (1:1000; Millipore), a goat anti-glial fibrillary acidic protein (GFAP) antibody (1:250; Santa Cruz Biotechnology), or a rabbit anti-ionized calcium-binding adapter molecule (IBA1), also known as allograft inflammatory factor 1 (AIF-1), antibody (1:500; Proteintech) (Table 2). The antibodies were diluted with 3% normal serum and 0.2% Triton X-100 in PBS. After being washed in PBS, the sections were incubated for 1 h with an appropriate solution of biotinylated secondary antibodies (goat anti-rabbit, horse anti-mouse or rabbit anti-goat IgG, 1:500; Vector Laboratories), followed by a 1 h incubation with an avidin–biotin-peroxidase complex (1:200, 1 h; Vectastain ABC Kit, Vector Laboratories). The immunochemical reaction was developed in a diaminobenzidine (DAB)-nickel solution containing 0.02% DAB, 0.01% H_2_O_2_ and 0.06% NiCl_2_ in TBS, which stained the immunoreactive material black. The sections were mounted onto gelatin-coated slides, air-dried and coverslipped using Permount (Fisher Scientific) as the mounting medium.

### Cell counting

The number of immunoreactive (IR) cells was estimated by unbiased stereological methods [49], as described previously by Chocyk et al. [8, 39] and Majcher-Maślanka et al. [10]. Briefly, every fourth section, selected by systematic random sampling along the rostrocaudal axis of the mPFC, was chosen for analysis (10–11 sections per animal). Optical fractionator sampling was performed using a Leica DM 6000 B microscope equipped with a motorized stage (Ludl Electronic Products, Hawthorne, NY, USA) connected to a controller (MAC 5000, Ludl) and a digital camera (MBF C × 9000, Williston, VT, USA). Sampling was performed using Stereo Investigator 8.0 software (MBF Bioscience, Williston, VT, USA) by experimenters unaware of treatment group allocation to the specific slides. The studied regions of the mPFC, i.e., the Cg1, PLC and ILC, were outlined under low magnification (2.5×) according to a stereotaxic atlas of the rat brain [48] (Fig. 1). Sampling was performed bilaterally under high magnification (63×, oil-immersion objective) using counting frames with areas of 2500 µm^2^ and heights of 15 µm. The sampling parameters resulted in the mean of 1277 NeuN-IR cell counted (range 724–2161), 471 IBA1-IR cells counted (range 228–723) and 642 GFAP-IR cells counted (range 397–995) in each subregion of the mPFC. The total number of immunoreactive (IR) cells per region was estimated from the number of cells sampled within the optical dissectors and calculated by multiplying the numerical density of the cells (the number of IR cells/mm^3^) by the regional volume occupied by the cells within the studied region. The regional volumes of the studied mPFC areas in each animal were determined using the Cavalieri method [50]. The final results are presented as the estimated total number of cells within the specific regions.

### The light/dark exploration test

On PND 26, separate groups of rats (10 rats per experimental group) were subjected to the light/dark exploration test to assess anxiety-like behaviors. The light/dark exploration test was performed as described previously by Chocyk et al. [8, 37] and Solarz et al. [30]. Briefly, each experimental cage included an arena (45 × 45 × 45 cm) with a light compartment made of clear acrylic and a dark compartment made of black acrylic. The black compartment covered 33% of the total cage area, and the black dividing wall was equipped with a central tunnel gate (11 × 8.4 cm). The light compartment was brightly illuminated (100 lx), whereas the dark compartment received no light at all. The animals were kept in total darkness for 30 min prior to the testing, and the entire experiment was conducted with the room lights off. The animals were individually tested in single 10 min trials. Six weeks later, the same groups of rats were retested for anxiety-like behaviors when they approached adulthood (PND 70), together with additional 5 AFR, 2 VEH-MS and 2 SAL-MS adult rats. The behavioral responses during the test sessions were recorded using Fear Conditioning (FC) software (TSE, Bad Homburg, Germany). Specifically, the number of transitions between the compartments, time spent in each compartment, and locomotor activity (the distance traveled) were measured.

### Fear conditioning

Behavioral tests were performed and analyzed using a computer-controlled FC system (TSE, Bad Homburg, Germany) as described previously by Chocyk et al. [38] and Bialon et al. [51]. Each FC unit consisted of sound-attenuating housing with a loudspeaker, camera, ventilation fan and 4 symmetrically mounted lamps in the ceiling construction and test box. The test box comprised the test arena and a base construction containing integrated infrared animal detection sensors in the X, Y (horizontal) and Z (vertical) axes. The sensor frames along the axes were equipped with 32 sensor pairs mounted 14 mm apart. All sensors were scanned at a sampling rate of 100 Hz, i.e., the position of the animal was checked 100 times per second. Several pilot experiments were run that compared automatic and manual scoring of freezing behavior; the data obtained from each method were highly correlated.

During the experimental procedure, the animals were tested in two different arenas and contexts (A and B). For the first context (Context A), the arena (46 × 46 × 47 cm) was made of transparent acrylic and had a floor made up of stainless steel rods (4 mm in diameter) spaced 8.9 mm apart (center to center). The floor was connected to a shocker-scrambler unit for delivering shocks of defined duration and intensity. The arena was cleaned with 1% acetic acid solution. A ventilation fan provided background noise (65 dB), and lamps provided uniform illumination of 60 lx inside the fear conditioning housing. During tests in Context A, the room lights remained on. Animals were transported to this context with transparent plastic boxes. Experimenters wore white clothes and gloves.

For the second context (Context B), the arena (46 × 46 × 47 cm) was made of black acrylic (permeable to infrared light) with a gray plastic floor. The arena was cleaned with 70% ethanol solution and faintly illuminated (4 lx). The tests in Context B were conducted with the room light off. Animals were transported to this context with black plastic boxes. Experimenters wore blue clothes and gloves.

Fear conditioning (FC) and memory were assessed using the Pavlovian paradigm. The schedule of FC procedures is presented in Table 3. On day 1 of experiment (PND 26), the animals (14 AFR rats and 10 rats in each other treatment group) were subjected to FC procedure in Context A (acquisition/training). Animals were placed in Context A and allowed to habituate for 180 s. Next, the animals received five tone-shock pairings in which the tone (amplitude: 80 dB; frequency: 2 kHz; duration: 10 s) was co-terminated with foot shock (intensity: 0.7 mA; duration: 1 s). The intertrial interval was 60 s. Animals were removed from Context A 60 s after the last trial.

**Table 3:** A schedule of FC procedures

PND	Procedure
26	FC acquisition/training (Context A)
27	CFC expression (Context A) AFC expression (Context B)
70	CFC memory test (recall) in adulthood (Context A)
71	AFC memory test (recall) in adulthood (Context B)
77	FC retraining in adulthood (Context A)
78	CFC expression after retraining in adulthood (Context A) AFC expression after retraining in adulthood (Context B)

*AFC* auditory fear conditioning, *CFC* contextual fear conditioning, *FC* fear conditioning, *PND* postnatal day

On day 2, all animals were once again exposed to Context A and were left undisturbed for 6 min (expression of contextual fear conditioning, CFC), then returned to their home cages. Two hours later, the animals were placed in a new context (Context B) and, after 180 s of habituation, received five presentations of tone-alone with 61-s intertrial intervals (expression of auditory fear conditioning, AFC). Animals were removed from Context B 60 s after the last trial.

Six weeks after the FC training (on PND 70), the same animals were once again tested in both Context A and B for recall of fear memories. First, they were placed and left undisturbed for 6 min in Context A, and 24 h later, they were exposed to five presentations of tone-alone in Context B (Table 3). Seven days later, all animals were subjected to a session of retraining of fear conditioning in Context A; retraining followed the same procedure as training during preadolescence (Day 1). On the following day, fear memory was tested both in Context A and B (the same sessions as on Day 2 during preadolescence) (Table 3).

Behavioral responses during all sessions were recorded and automatically analyzed using FC software (TSE, Bad Homburg, Germany). Freezing (i.e., immobility) was taken as the behavioral measure of fear and was defined as the absence of all non-respiratory movements for at least 2 s. The cumulative duration of freezing was calculated for each session and expressed as percentage of entire session time, excluding habituation time.

### Sucrose preference test

To assess anhedonic-like behavior, a sucrose preference was measured in a two-bottle choice paradigm as described previously by Chocyk et al. [8] and Solarz et al. [30]. Briefly, on PND 22 separate groups of rats (10 rats per experimental group) were singly housed and habituated to drink water from two bottles for 2 days. Then, the water in one of the bottles was replaced by 1% sucrose solution for 2 days to avoid neophobia. The position of the bottles (sucrose left or right) was reversed every 8 h to prevent the development of place preference. After habituation, the rats were subjected to water deprivation for 16 h before performing the sucrose preference test. During the test, two bottles, one containing tap water and another containing 1% sucrose solution, were presented to each rat for 4 h (from 8 a.m. to 12 p.m.). The positioning of the water and sucrose bottles (left or right) was balanced between the experimental groups. Six weeks later, the same groups of rats were retested for anhedonic-like behavior when they approached adulthood (PND 70). Sucrose preference was calculated as the percentage of sucrose intake versus total liquid intake (water + sucrose) over the 4-h test period.

### Measurement of novelty- and amphetamine-induced locomotor activity

On PND 26, a separate group of animals was subjected to the locomotor activity test (*n* = 9–12). Locomotor activity was recorded and analyzed individually for each animal using Opto-Varimex cages (43 × 44 cm) and Auto-Track software (Columbus Instruments, OH, USA), as described previously by Majcher-Maślanka et al. [10, 52]. The rats were placed into the test cages without previous habituation and were free to explore the environment for 20 min (novelty-induced locomotion, session 1). Next, the rats received vehicle injections (saline, 1 ml/kg *sc*) and were left in test cages for 50 min (session 2). Finally, the same rats were injected with amphetamine (1 mg/kg) and left in test cages for another 50 min (session 3). Six weeks later, the same groups of rats were retested for locomotor activity when they approached adulthood (PND 70). Locomotor activity of the animals was recorded for each session separately. The data are presented as the average distance traveled over the entire session.

### Statistical analysis

Statistical analysis of the data was performed using Statistica 13.3 software (TIBCO Software Inc., USA). Initially, data were tested for normal distribution and homogeneity of variances using Shapiro–Wilk test and Levene’s test, respectively. In the case of data that followed normal distribution and had equal variances among groups, they were further analyzed by one-way analysis of variance (ANOVA) with early-life treatment (AFR, MS, VEH-MS and SAL-MS) as independent variable followed by Tukey’s HSD post hoc test. Amphetamine-induced locomotor activity was specifically analyzed by mixed-design ANOVA with early-life treatment as a between-subject factor and saline and amphetamine injections as within-subject factor. In the case of data which did not show normal distribution and homogeneity of variances, statistical differences between experimental groups were analyzed by Kruskal–Wallis test followed by Dunn’s test for multiple comparisons. *P* values < 0.05 were considered significantly different. The data are presented as the group mean and standard deviation (SD, parametric statistics) or as the median and interquartile range (IQR, nonparametric statistics).

## Results

### The effects of MS and early-life SAL/VEH injections on body weight

**Fig. 2:**
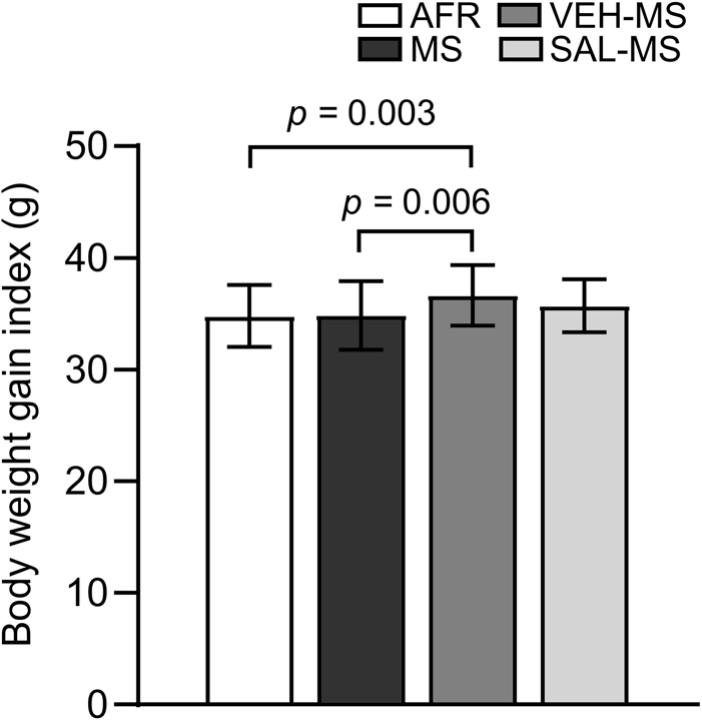
The effects of MS and early-life SAL/VEH injections on a body weight gain in juvenile rats. The data are presented as the mean ± SD (*n* = 48 − 64) and expressed as a body weight gain index calculated as the body weight difference between PND 15 and PND 1. Connectors indicate statistically significant differences between specific experimental groups (one-way ANOVA followed by Tukey’s HSD post hoc analysis). *AFR* animal facility rearing, *MS* maternal separation, *SAL* salubrinal, *VEH* vehicle

To examine whether specific early-life treatment, such as repeated MS and SAL/VEH injections, affected the body weight of juvenile rats, a body weight gain index between PND 1 and PND 15 was calculated. Statistical analysis revealed that early-life treatment significantly affected body weight gain in juvenile rats (*F*_3,220_ = 5.21, *p* = 0.002, one-way ANOVA followed by Tukey’s test). Specifically, VEH-MS rats showed a slightly higher body weight gain index in comparison to AFR and MS rats (Fig. 2).

Body weight was also assessed during the preadolescence and adulthood periods. However, Kruskal–Wallis test did not show any significant differences between the experimental groups (PND 26: *H*_3_ = 2.64, N_1_ = 31, N_2_ = 29, N_3_ = 26, N_4_ = 30, *p* = 0.450; PND 70: *H*_3_ = 1.46, N_1_ = 34, N_2_ = 25, N_3_ = 25, N_4_ = 26, *p* = 0.690) (Table 4).

**Table 4:** The effects of MS and early-life SAL/VEH treatment on body weight of preadolescent and adult rats

PND	Group	Body weight
26	AFR	90.0 (11.5)
	MS	92.5 (13.8)
	VEH-MS	93.2 (15.0)
	SAL-MS	93.0 (19.1)
70	AFR	440.5 (63.0)
	MS	449.0 (26.0)
	VEH-MS	442.0 (82.0)
	SAL-MS	449.5 (87.0)

Data are presented as the median (IQR) (*n* = 25–34) and expressed in grams. *AFR* animal facility rearing, *IQR* interquartile range, *MS* maternal separation, *PND* postnatal day, *SAL* salubrinal, *VEH* vehicle

### The effects of MS and early-life SAL/VEH injections on ER stress and UPR processes in juvenile rats (PND 15)

To study the early effects of repeated MS and SAL/VEH injections on ER stress and UPR processes, we examined the gene and protein expression of ER stress and UPR markers in the mPFC of rats on PND 15, which was 24 h after the last MS procedure.

**Fig. 3:**
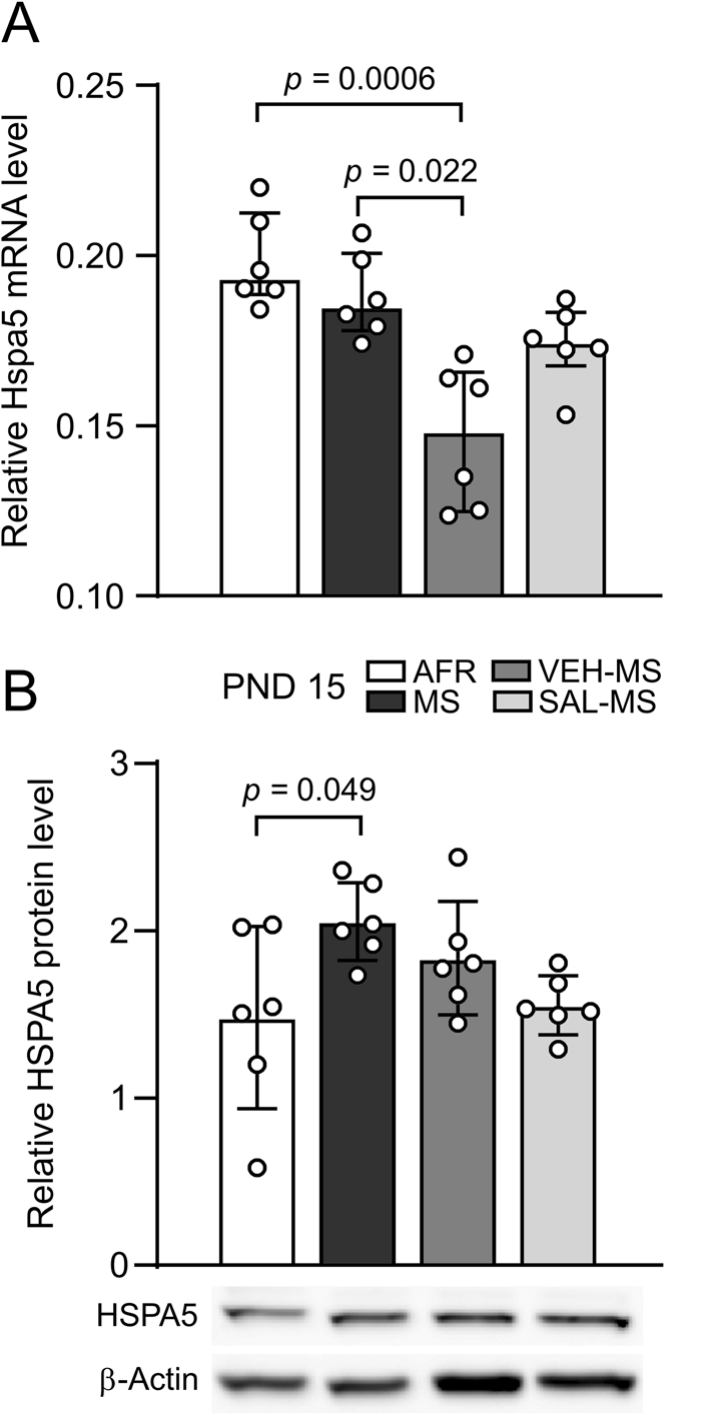
The effects of MS and early-life SAL/VEH injections on HSPA5 mRNA (**A**) and protein (**B**) expression in the mPFC of juvenile rats. The data are presented as the median and IQR (**A**) or mean ± SD (**B**) and were analyzed by Kruskal–Wallis test or one-way ANOVA, respectively (*n* = 6). Circles represent individual data points. Connectors indicate statistically significant differences between specific experimental groups in Dunn’s post hoc test (**A**) or Tukey’s HSD post hoc test (**B**). *AFR* animal facility rearing, *MS* maternal separation, *PND* postnatal day, *SAL* salubrinal, *VEH* vehicle

Statistical analysis of Hspa5 mRNA expression in the mPFC showed a significant effect of early-life treatment (Kruskal–Wallis test, *H*_3_ = 17.02, N_1–4_ = 6, *p* = 0.0007). Specifically, VEH-MS rats had lower Hspa5 mRNA levels than AFR and MS rats (Dunn’s test) (Fig. 3A).

The analysis of HSPA5 (GRP 78) protein levels in the mPFC of juveniles also revealed a significant effect of early-life treatment (one-way ANOVA, *F*_3,20_ = 3.35, *p* = 0.039). In this case, MS rats showed enhanced expression of HSPA5 protein in comparison to AFR rats (Tukey’s test) (Fig. 3B).

Next, we examined the effect of MS and SAL/VEH on the mRNA and protein expression of the ER stress sensors Eif2ak3 (PERK), Ern1 (IRE1α) and ATF6 in the mPFC.

**Fig. 4:**
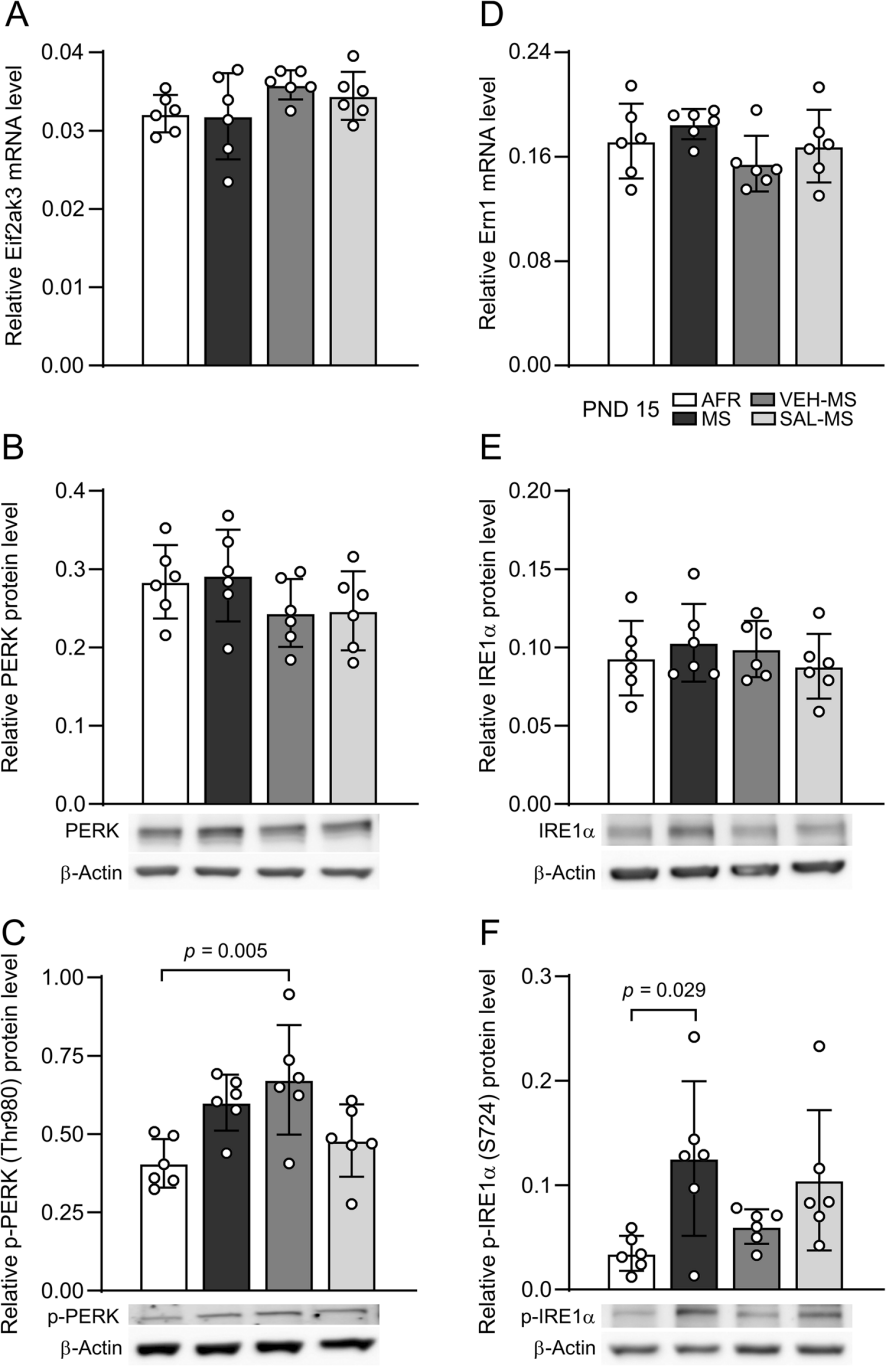
The effects of MS and early-life SAL/VEH injections on mRNA expression and protein expression and phosphorylation of ER stress sensors PERK (**A**–**C**) and IRE1α (**D**–**F**) in the mPFC of juvenile rats. The data are presented as the mean ± SD (*n* = 6) and were analyzed by one-way ANOVA. Circles represent individual data points. Connectors indicate statistically significant differences between specific experimental groups in Tukey’s HSD post hoc test. *AFR* animal facility rearing, *MS* maternal separation, *PND* postnatal day, *SAL* salubrinal, *VEH* vehicle

In the case of all ER stress sensors, analysis of their mRNA expression showed no significant differences between experimental groups (one-way ANOVA, Online Resource ESM_1: Table S1; ESM_2: Fig. S1A) (Fig. 4A, D). Moreover, we did not observe an effect of MS and SAL/VEH on the total protein levels of PERK, IRE1α and ATF6 (one-way ANOVA, Table S1) (Fig. 4B, E; ESM_2: Fig. S1B). However, we did find a significant effect of early-life treatment on activation by phosphorylation of PERK at residue Thr980 (*F*_3,20_ = 5.93, *p* = 0.005, one-way ANOVA followed by Tukey’s test). Specifically, VEH-MS rats showed increased levels of p-PERK (Thr980) protein in comparison to AFR rats (Fig. 4C). A similar trend was also observed in the case of the MS group, though it did not reach statistical significance (*p* = 0.051). Nevertheless, when a single comparison between AFR and MS groups was performed, the effect was observed to be statistically significant (*U* = 2, N_1-2_ = 6, *p* = 0.013, Mann‒Whitney *U* test). Analysis of the phosphorylation of IRE1α at residue S724 also showed a significant effect of early-life treatment (one-way ANOVA,* F*_3,20_ = 3.88, *p* = 0.024). In this case, MS increased the phosphorylation of IRE1α at S724 in comparison to AFR rats (Tukey’s test) (Fig. 4F).

**Fig. 5:**
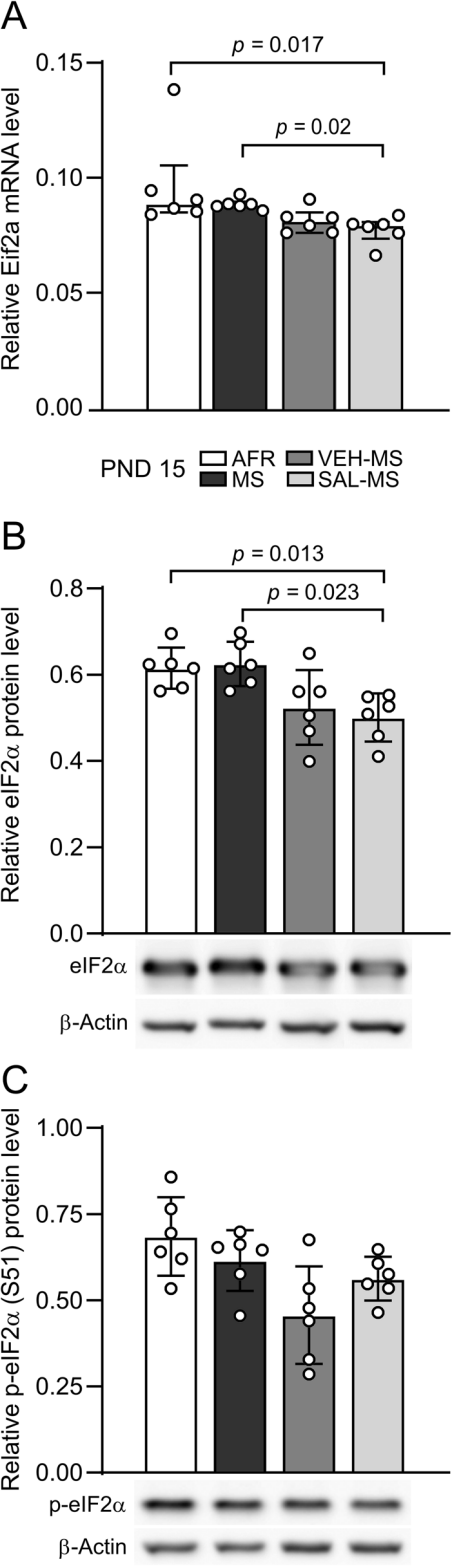
The effects of MS and early-life SAL/VEH injections on mRNA expression (**A**) and protein expression and phosphorylation (**B**–**C**) of eIF2α in the mPFC of juvenile rats. The data are presented as the median and IQR (**A**) or mean ± SD (**B**, **C**) and were analyzed by Kruskal–Wallis test or one-way ANOVA, respectively (*n* = 6). Circles represent individual data points. Connectors indicate statistically significant differences between specific experimental groups in Dunn’s post hoc test (**A**) or Tukey’s HSD post hoc test (**B**). The same immunoblot of β-Actin was used for normalization of both p-eIF2α (**C**) and p-IRE1α immunoblots (Fig. 4F). After protein electrotransfer the blots were horizontally cut into two pieces to separately evaluate p-eIF2α and p-IRE1α protein levels from the same samples. Next, after membrane stripping procedure, appropriate blot was reprobed with β-Actin antibody. *AFR* animal facility rearing, *MS* maternal separation, *PND* postnatal day, *SAL* salubrinal, *VEH* vehicle

Because SAL acts as an inhibitor of eIF2α dephosphorylation at residue S51, we also investigated how repeated MS and SAL/VEH injections impacted the expression and phosphorylation of eIF2α in the mPFC of juveniles. We observed that SAL-MS rats showed lower levels of Eif2a mRNA than MS and AFR rats (*H*_3_ = 18.83, N_1-4_ = 6, *p* = 0.003, Kruskal–Wallis test followed by Dunn’s test) (Fig. 5A). We also observed a similar effect in total protein of eIF2α (*F*_3,20_ = 6.10, *p* = 0.004, ANOVA followed by Tukey’s test) (Fig. 5B). However, one-way ANOVA of eIF2α phosphorylation did not reveal any differences in the level of p-eIF2α (S51) between the experimental groups (*F*_3,20_ = 4.97, *p* = 0.050) (Fig. 5C).

### The effects of MS and early-life SAL/VEH injections on apoptotic processes in the mPFC of juvenile rats (PND 15)

Since ER stress and UPR processes are known to affect cell death and survival decisions, the next goal of our study was to examine the gene and protein expression of the main apoptotic markers in the mPFC.

**Fig. 6:**
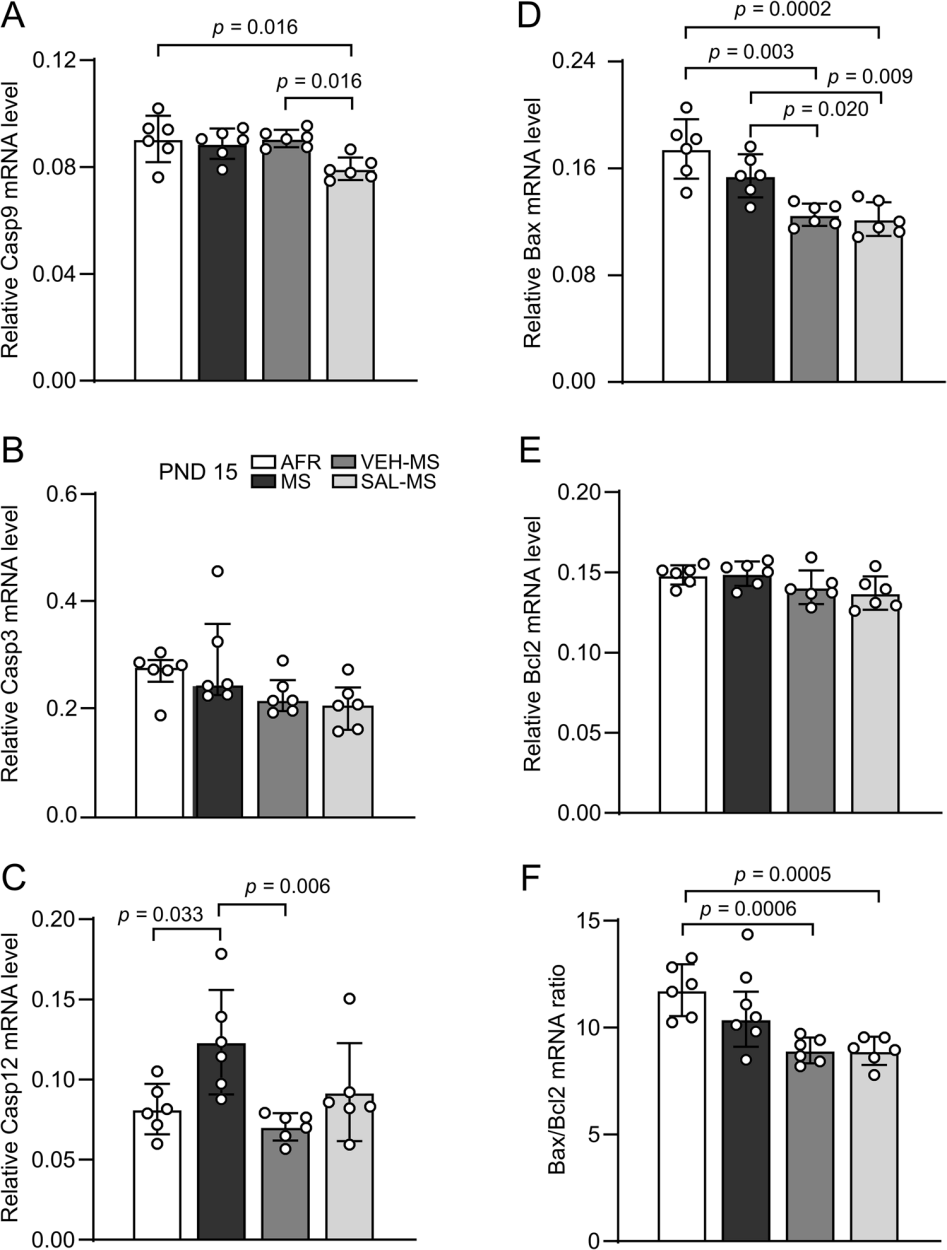
The effects of MS and early-life SAL/VEH injections on mRNA expression of apoptotic markers in the mPFC of juvenile rats: Casp9 (**A**), Casp3 (**B**), Casp12 (**C**), Bax (**D**), Bcl2 (**E**), and Bax/Bcl2 mRNA ratio (**F**). The data are presented as the mean ± SD (**A**, **C**–**F**) or median and IQR (**B**) and were analyzed by one-way ANOVA or Kruskal–Wallis test, respectively (*n* = 6). Circles represent individual data points. Connectors indicate statistically significant differences between specific experimental groups in Tukey’s HSD post hoc test. *AFR* animal facility rearing, *MS* maternal separation, *PND* postnatal day, *SAL* salubrinal, *VEH* vehicle

Statistical analysis showed that early-life MS and SAL/VEH treatment affected the mRNA expression of a few apoptotic markers but not their protein levels (Online Resource ESM_1: Table S1; ESM_2: Fig. S2; Fig. S3) (Fig. 6). Notably, we observed that MS rats subjected to SAL treatment had lower levels of caspase-9 mRNA in comparison to that of VEH-MS and AFR rats (*F*_3,20_ = 5.11, *p* = 0.009, one-way ANOVA followed by Tukey’s test) (Fig. 6A). A trend toward reduced expression of caspase-9 was also observed in the SAL-MS group compared to MS rats, though it did not reach statistical significance (*p* = 0.051). On the other hand, we did not observe any differences between the experimental groups in caspase-3 mRNA expression, a key effector caspase involved in a mitochondrial pathway of apoptosis (Kruskal–Wallis test, *H*_3_ = 7.13, N_1–4_ = 6, *p* = 0.068) (Fig. 6B).

Interestingly, our study also revealed a statistically significant effect of early-life treatment on the mRNA expression of caspase-12, a specific caspase that is localized in the ER membrane and engaged in the ER stress-induced pathway of apoptosis (one-way ANOVA, *F*_3,20_ = 5.36, *p* = 0.007). The MS procedure increased caspase-12 mRNA expression compared to that in the AFR group (Fig. 6C). Moreover, VEH-MS rats had a significantly lower level of caspase-12 mRNA than MS rats (Tukey’s test).

As described above, we did not observe a statistically significant effect of early-life treatment on procaspase-9 or procaspase-12 protein expression or cleaved (active) caspase-9, -12 or cleaved caspase-3 protein levels (one-way ANOVA, Table S1) (ESM_2: Fig. S2). Nevertheless, in the case of cleaved caspase-9, when a single comparison between AFR and MS groups was performed using a Student’s *t*-test, statistical significance was observed, and MS rats showed increased cleaved caspase-9 protein levels in the mPFC (*t*_10_ = 2.56, *p* = 0.023) (Fig. S2B).

Next, we examined Bax and Bcl2 expression and the Bax/Bcl2 ratio and observed a significant effect of early-life treatment on Bax mRNA levels (*F*_3,20_ = 15.34, *p* < 0.0001, ANOVA) and the Bax/Bcl2 mRNA ratio (*F*_3,20_ = 11.29, *p* < 0.0001) (Fig. 6D–F). Specifically, both SAL- and VEH-injected MS rats showed lower levels of Bax mRNA compared to MS and AFR rats (Tukey’s test) (Fig. 6D). The Bax/Bcl2 mRNA ratio was decreased in SAL-MS and VEH-MS rats compared to AFR rats only (Tukey’s test) (Fig. 6F). There was no statistically significant effect of early-life treatment on the protein expression of Bax or Bcl2 or the Bax/Bcl2 protein ratio (one-way ANOVA, ESM_1: Table S1; ESM_2: Fig. S3). Nevertheless, in the case of Bax protein expression, when a single comparison between AFR and MS groups was performed using a Student’s *t*-test, statistical significance was observed, and the MS rats showed increased Bax protein levels in the mPFC (*t*_10_ = 3.08, *p* = 0.012) (Fig. S3A).

### The effects of MS and early-life SAL/VEH injections on ER stress and UPR processes in preadolescent rats (PND 26)

Preadolescence and adolescence are crucial periods in mPFC postnatal maturation. During the preadolescence period, neurodevelopmental apoptosis starts to progress. The next goal of our study was to determine whether early-life MS and SAL/VEH treatment affect ER stress and UPR processes specifically in preadolescent rats (on PND 26) and in this way influence neurodevelopmental apoptosis in the mPFC.

**Fig. 7:**
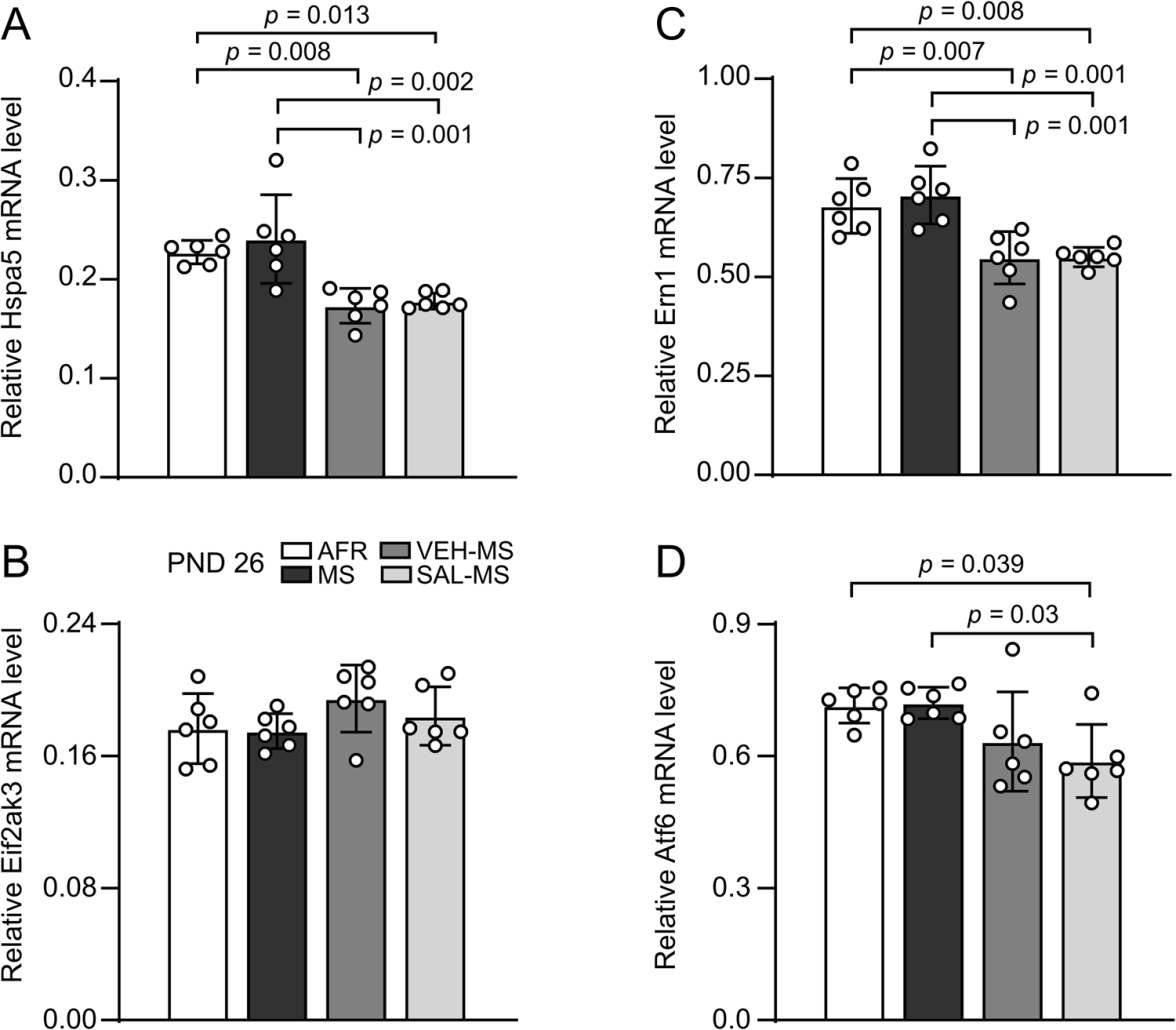
The effects of MS and early-life SAL/VEH injections on mRNA expression of Hspa5 (**A**) and ER stress sensors Eif2ak3 (**B**), Ern1 (**C**), Atf6 (**D**) in the mPFC of preadolescent rats. The data are presented as the mean ± SD (*n* = 6) and were analyzed by one-way ANOVA. Circles represent individual data points. Connectors indicate statistically significant differences between specific experimental groups in Tukey’s HSD post hoc test. *AFR* animal facility rearing, *MS* maternal separation, *PND* postnatal day, *SAL* salubrinal, *VEH* vehicle

Statistical analysis showed that in preadolescent rats, early-life treatment only affected mRNA expression of Hspa5 (*F*_3,20_ = 11.23, *p* < 0.0001) and two ER stress sensors, Ern1 (*F*_3,20_ = 11.15, *p* = 0.0002**)** and Atf6 (*F*_3,20_ = 4.42, *p* = 0.015) (one-way ANOVA) (Fig. 7). We did not observe differences between experimental groups in protein expression or activation (phosphorylation) of any of the ER stress or UPR markers we examined (ESM_1: Table S1) (ESM_3: Fig S4; Fig S5). Post hoc analysis of mRNA expression showed that both SAL- and VEH-injected MS rats had lower levels of Hspa5 mRNA compared to MS and AFR rats (Tukey’s test) (Fig. 7A). A similar effect of early-life treatment was also observed in the case of Ern1 (Fig. 7C). However, analysis of Atf6 mRNA levels showed that only SAL-injected MS rats had reduced Atf6 mRNA expression compared to MS and AFR (Tukey’s test) (Fig. 7D).

Next, we examined Eif2a mRNA and protein expression and eIF2α phosphorylation at residue S51. Statistical analysis revealed a lack of any significant differences between experimental groups in all these parameters (Table S1) (ESM_3: Fig. S5).

### The effects of MS and early-life SAL/VEH injections on apoptotic processes in the mPFC of preadolescent rats (PND 26)

**Fig. 8:**
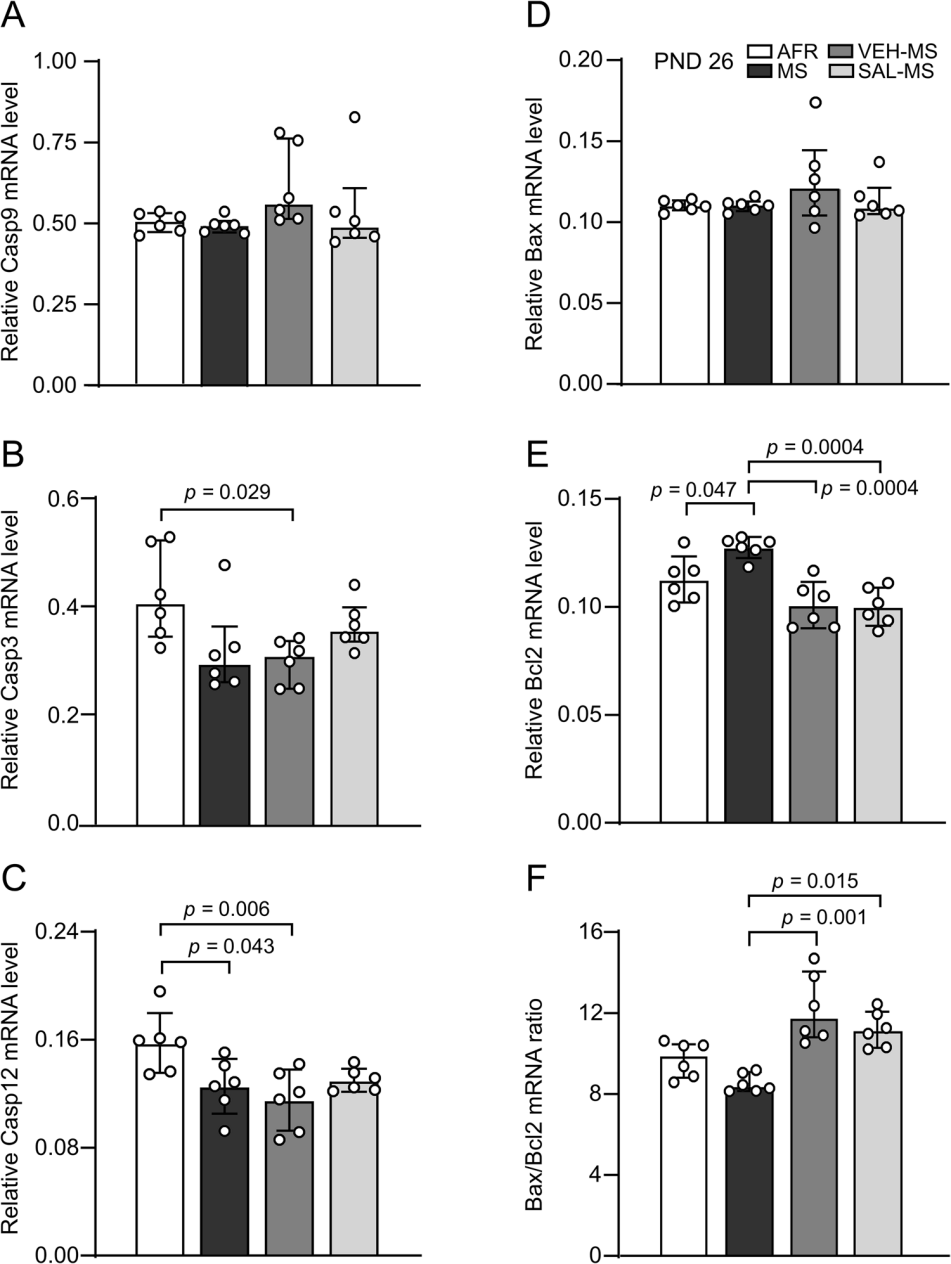
The effects of MS and early-life SAL/VEH injections on mRNA expression of apoptotic markers in the mPFC of preadolescent rats: Casp9 (**A**), Casp3 (**B**), Casp12 (**C**), Bax (**D**), Bcl2 (**E**), and Bax/Bcl2 mRNA ratio (**F**). The data are presented as the median and IQR (**A**, **B**, **D**, **F**) or mean ± SD (**C**, **E**) and were analyzed by Kruskal–Wallis test or one-way ANOVA, respectively (*n* = 6). Circles represent individual data points. Connectors indicate statistically significant differences between specific experimental groups in Dunn’s post hoc test (**B**, **F**) or Tukey’s HSD post hoc test (**C**, **E**). *AFR* animal facility rearing, *MS* maternal separation, *PND* postnatal day, *SAL* salubrinal, *VEH* vehicle

Statistical analysis of the mRNA expression of the analyzed caspases showed a significant effect of early-life treatment on caspase-3 (*H*_3_ = 10.82, N_1–4_ = 6, *p* = 0.013, Kruskal–Wallis test) and caspase-12 (*F*_3,20_ = 5.26, *p* = 0.008, ANOVA**)** but not caspase-9 mRNA levels (*H*_3_ = 6.76, N_1–4_ = 6, *p* = 0.080) (Online Resource ESM_1: Table S1) (Fig. 8). VEH-injected MS rats showed a lower level of caspase-3 mRNA than AFR rats (Dunn’s test) (Fig. 8B). Both MS and MS-VEH rats showed reduced caspase-12 mRNA expression compared to AFR rats (Tukey’s test) (Fig. 8C). However, statistical analysis did not reveal any significant differences between experimental groups in protein levels of procaspase-12 or -9 or cleaved forms of caspase-12, -9 or -3 (Table S1) (ESM_3: Fig. S6A–E).

Next, we investigated the effects of MS and SAL/VEH injections on the expression of Bcl-2 family members. Statistical analysis revealed that early-life treatment significantly affected Bcl2 mRNA expression (*F*_3,20_ = 12.02, *p* = 0.0001, ANOVA) and the Bax/Bcl2 mRNA ratio (*H*_3_ = 16.61, N_1–4_ = 6, *p* = 0.0009) but not Bax mRNA levels (*H*_3_ = 1.32, N_1–4_ = 6, *p* = 0.724, Kruskal–Wallis test) (Fig. 8). Specifically, MS increased Bcl2 mRNA expression compared to that in AFR rats, and this effect was prevented in MS rats by both VEH and SAL injections (Tukey’s test) (Fig. 8E). On the other hand, post hoc analysis of the Bax/Bcl2 mRNA ratio revealed that this parameter was similar in AFR and MS rat, but both VEH- and SAL-injected MS rats showed an increased Bax/Bcl2 mRNA ratio compared to MS rats (Dunn’s test) (Fig. 8F). However, analysis of the Bax/Bcl2 protein ratio revealed a different effect, namely that both MS and VEH-MS rats showed a lower level of this parameter compared to AFR rats (Kruskal–Wallis test, *H*_3_ = 11.29, N_1–4_ = 6, *p* = 0.010, followed by Dunn’s test) (ESM_3: Fig. S7C). No change in the protein levels of Bax and Bcl2 was observed between the treatment groups (Online Resource ESM_1: Table S1) (Fig. S7A–B).

Finally, we investigated whether repeated MS and SAL/VEH treatment affected the number of neurons and glial cells in specific subregions of the mPFC in preadolescent rats. Statistical analysis revealed that early-life treatment did not have any significant impact on the number of neurons (NeuN-IR), astrocytes (GFAP-IR) or microglial cells (IBA1-IR) in the mPFC of preadolescent rats (results and statistics are presented in Table 5). Representative photomicrographs showing neuronal and glial cells in the subregions of the mPFC of preadolescent rats are presented in Online Resource ESM_5˗7.

**Table 5:** The effects of MS and early-life SAL/VEH treatment on the number of neurons astrocytes and microglial cells in the mPFC of preadolescent rats

Cell marker	mPFC region	Group	Number of IR cells	Statistic
NeuN	Cg1	AFR	685,481.1 ± 59,206.6	*F*_3,18_ = 0.20, *p* = 0.896
		MS	698,794.9 ± 45,376.6	
		VEH-MS	681,216.5 ± 28,241.7	
		SAL-MS	684,556.8 ± 35,757.5	
	PLC	AFR	1,250,600.6 ± 132,051.7	*F*_3,18_ = 0.27, *p* = 0.844
		MS	1,281,136.8 ± 57,292.6	
		VEH-MS	1,252,841.1 ± 64,980.4	
		SAL-MS	1,291,928.6 ± 103,713.6	
	ILC	AFR	288,312.2 (35,984.3)	*H*_3_ = 0.88, *p* = 0.830
		MS	275,936.1 (29,923.8)	
		VEH-MS	275,092.7 (7595.9)	
		SAL-MS	281,630.6 (24,893.4)	
GFAP	Cg1	AFR	204,453.6 ± 29,435.2	*F*_3,18_ = 0.80, *p* = 0.510
		MS	200,720.3 ± 37,443.2	
		VEH-MS	189,177.3 ± 11,617.8	
		SAL-MS	181,914.7 ± 26,710.8	
	PLC	AFR	374,166.3 ± 67,722.5	*F*_3,18_ = 0.48, *p* = 0.700
		MS	359,114.3 ± 34,219.7	
		VEH-MS	347,362.8 ± 46,345.4	
		SAL-MS	338,200.3 ± 55,135.5	
	ILC	AFR	120,888.7 ± 18,295.7	*F*_3,18_ = 0.47, *p* = 0.704
		MS	118,740.8 ± 17,268.8	
		VEH-MS	114,362.4 ± 18,440.6	
		SAL-MS	108,330.1 ± 21,168.5	
IBA1	Cg1	AFR	212,681.4 ± 26,186.8	*F*_3,18_ = 0.61, *p* = 0.616
		MS	202,346.7 ± 13,552.4	
		VEH-MS	197,790.9 ± 19,641.8	
		SAL-MS	200,501.6 ± 15,313.6	
	PLC	AFR	319,342.3 ± 29,654.3	*F*_3,18_ = 0.23, *p* = 0.872
		MS	323,430.3 ± 31,567.8	
		VEH-MS	310,514.0 ± 46,495.7	
		SAL-MS	326,914.1 ± 30,180.7	
	ILC	AFR	73,607.2 ± 9470.6	*F*_3,18_ = 0.19, *p* = 0.902
		MS	76,013.2 ± 6420.1	
		VEH-MS	73,424.5 ± 5295.1	
		SAL-MS	73,042.3 ± 6925.9	

Data indicate the numbers of IR cells per region estimated by stereological method (the mean ± SD or median (IQR), *n* = 5–6). *AFR* animal facility rearing, *Cg1* cingulate cortex, *ILC* infralimbic cortex, *IQR* interquartile range, *IR* immunoreactive, *mPFC* medial prefrontal cortex, *MS* maternal separation, *PLC* prelimbic cortex, *PND* postnatal day, *SAL* salubrinal, *VEH* vehicle

### The effects of MS and early-life SAL/VEH injections on the anxiety-like behavior of preadolescent and adult rats in the light/dark box test

**Fig. 9:**
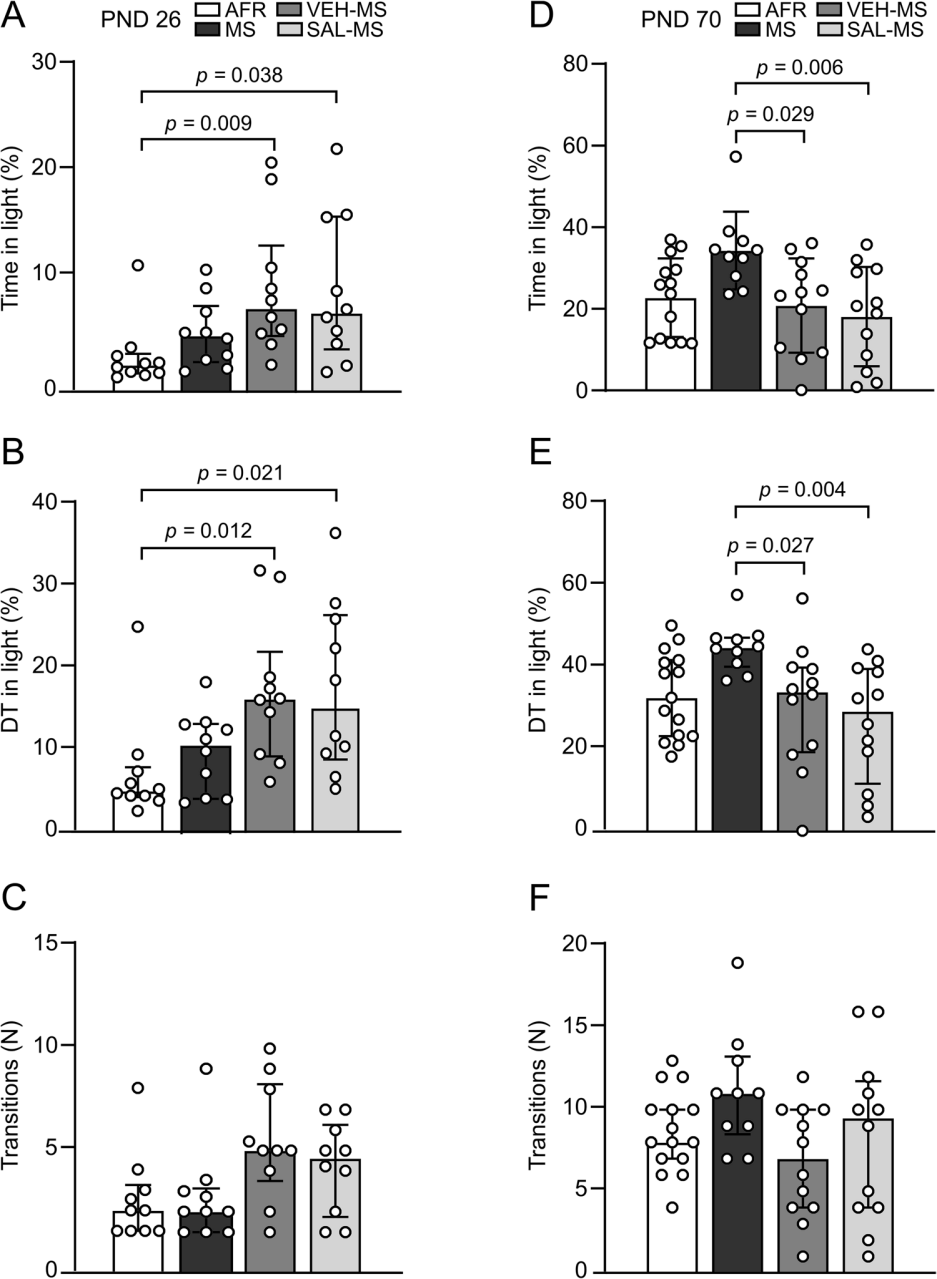
The effects of MS and early-life SAL/VEH injections on anxiety-like behavior of preadolescent (**A**–**C**) and adult rats (**D**–**F**) in the light/dark box test. Intensity of anxiety-like behavior was assessed as the time spent in the light side (**A**, **D**) and distance traveled in the light side (**B**, **E**) (both expressed as the percentage of the entire session) and the number of transitions between the dark and light sides (**C**, **F**). The data are presented as the median and IQR (**A**–**C**, **E**, **F**) or mean ± SD (**D**) and were analyzed by Kruskal–Wallis test or one-way ANOVA, respectively (*n* = 10 − 15). Circles represent individual data points. Connectors indicate statistically significant differences between specific experimental groups in Dunn’s post hoc test (**A**, **B**, **E**) or Tukey’s HSD post hoc test (**D**). *AFR* animal facility rearing, *DT* distance traveled, *MS* maternal separation, *PND* postnatal day, *SAL* salubrinal, *VEH* vehicle

The next goal of our study was to investigate the effects of early-life MS and SAL/VEH treatment on the behavioral phenotypes of both preadolescent and adult rats. We started by assessing anxiety-like behaviors in the light/dark box test. The experimental procedure for the light/dark exploration applied in the present study has been previously tested in adolescent (PND 35) [10] and adult rats [30]. Nevertheless, this specific procedure when performed on PND 26 in preadolescent rats induced a very high level of anxiety in the light compartment. For example, preadolescent AFR rats on average spent only 2.4% of a trial time (14.5 s) in the light compartment, and the distance traveled in the light compartment represented only 5.7% of the total distance traveled during the entire session (Fig. 9A–B). The average number of transitions between dark and light compartments was 2.6 for AFR rats (Fig. 9C). However, Kruskal–Wallis test followed by Dunn’s test revealed that VEH- and SAL-injected MS rats were significantly less fearful during this light/dark exploration procedure than AFR rats (Online Resource ESM_1: Table S2) (Fig. 9). Specifically, VEH-MS and SAL-MS preadolescent rats spent more time in the light compartment than AFR rats (*H*_3_ = 11.97, N_1–4_ = 10, *p* = 0.007) (Fig. 9A). Additionally, they traveled a longer distance in the light compartment than the AFR rats (*H*_3_ = 12.87, N_1–4_ = 10, *p* = 0.005) (Fig. 9B). There was also a trend toward a greater number of transitions between light and dark compartments for VEH-MS rats compared to AFR rats (*H*_3_ = 8.58, N_1–4_ = 10, *p* = 0.035, post hoc: *p* = 0.084) (Fig. 9C).

In adulthood, statistical analysis also revealed a significant effect of early-life treatment on the time spent in the light compartment (*F*_3,45_ = 4.51, *p* = 0.007, ANOVA) and the distance traveled in the light compartment (*H*_3_ = 12.83, *p* = 0.005) but not on the number of transitions between compartments (*H*_3_ = 6.0, N_1_ = 15, N_2_ = 10, N_3–4_ = 12, *p* = 0.112, Kruskal–Wallis test) (Fig. 9D–F). Adult MS rats showed a trend toward less fearful behavior during the light/dark exploration test than AFR rats (Fig. 9D–E), which is in agreement with our previous studies [8, 30]. However, in this experiment we did not observe statistical significance in a post hoc analysis (for the time in light: *p* = 0.093; for the distance traveled: *p* = 0.082). Nevertheless, we did observe a statistically significant effect when we performed a single comparison between the AFR and MS groups (for the time in light: *t*_23_ = 2.65, *p* = 0.014; for the distance traveled: *U* = 31, N_1_ = 15, N_2_ = 10, *p* = 0.016). Interestingly, both VEH-MS and SAL-MS adult rats showed more fearful behavior compared to MS rats but not AFR rats. Notably, they spent less time in the light compartment than MS rats (Tukey’s test) (Fig. 9D). They also traveled a shorter distance in the light compartment than the MS rats (Dunn’s test) (Fig. 9E).

### The effects of MS and early-life SAL/VEH injections on fear conditioning and memory in preadolescent and adult rats

**Fig. 10:**
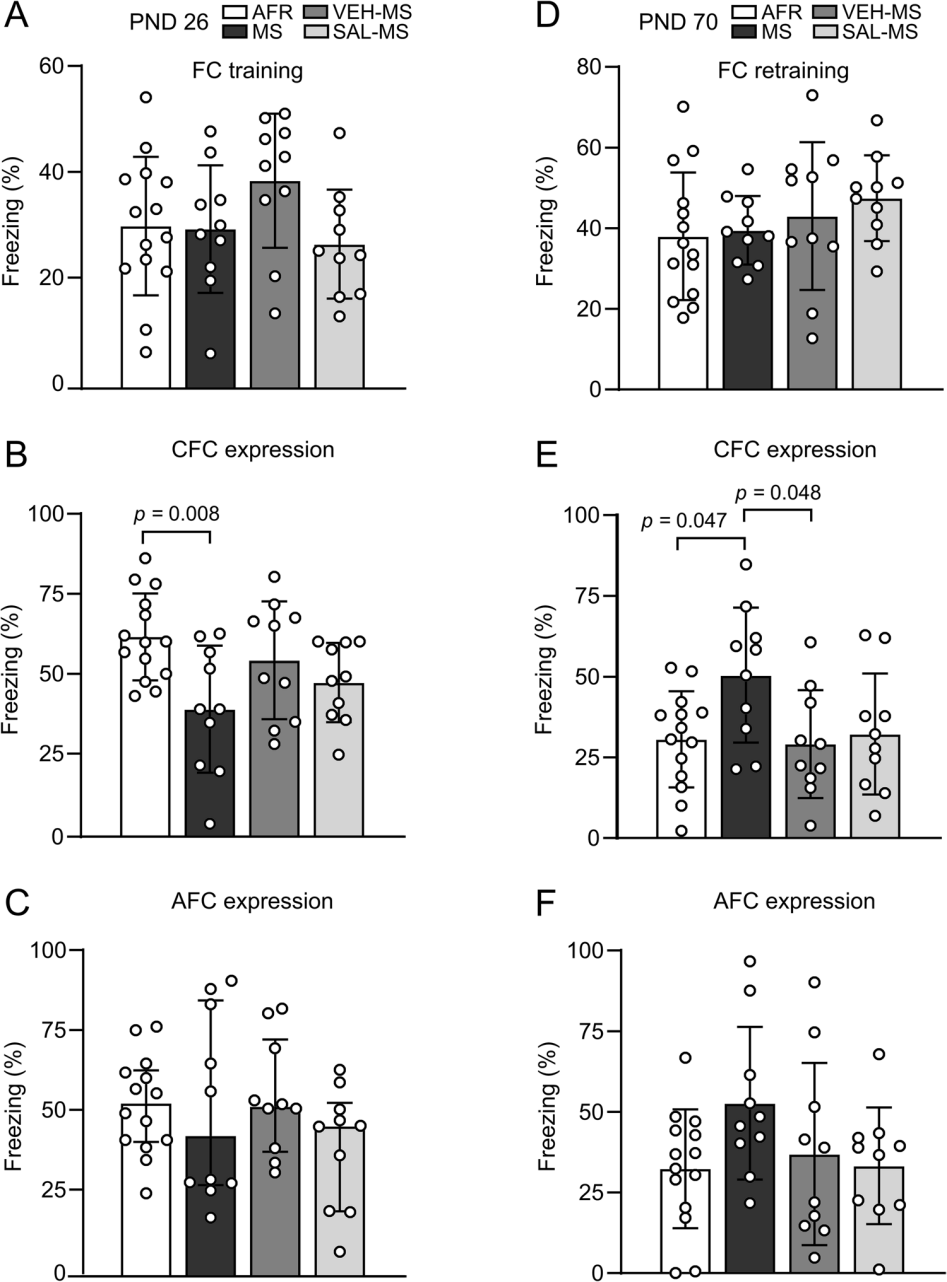
The effects of MS and early-life SAL/VEH injections on fear conditioning and memory in preadolescent (**A**–**C**) and adult rats (**D**–**F**). The data are presented as the mean ± SD (**A**, **B**, **D**–**F**) or median and IQR (**C**) and expressed as a percentage of the session time (*n* = 10–14). Results were analyzed by one-way ANOVA or Kruskal–Wallis test, respectively. Circles represent individual data points. Connectors indicate statistically significant differences between specific experimental groups in Tukey’s HSD post hoc test. *AFC* auditory fear conditioning, *AFR* animal facility rearing, *CFC* contextual fear conditioning, *FC* fear conditioning, *MS* maternal separation, *PND* postnatal day, *SAL* salubrinal, *VEH* vehicle

On PND 26, preadolescent rats underwent the FC procedure. Statistical analysis of freezing behavior during the acquisition/training session (Day 1, context A) revealed a lack of a significant effect of early-life treatment (one-way ANOVA, *F*_3,40_ = 1.82, *p* = 0.160) (Online Resource ESM_1: Table S2) (Fig. 10A). However, when the expression of CFC was analyzed (Day 2, context A), we observed that MS rats showed reduced freezing in response to context A compared to AFR rats (*F*_3,40_ = 4.20, *p* = 0.011, ANOVA followed by Tukey’s test) (Fig. 10B). The behavior of VEH-MS and SAL-MS rats did not differ significantly from MS rats (for VEH-MS: *p* = 0.162; for SAL-MS: *p* = 0.654) or AFR rats (for VEH-MS: *p* = 0.689; for SAL-MS: *p* = 0.156, Tukey’s test). Additionally, there was no significant effect of early-life treatment on the expression of AFC (Day 2, context B) in preadolescent rats (*H*_3_ = 2.91, N_1_ = 14, N_2–4_ = 10, *p* = 0.405, Kruskal–Wallis test) (Fig. 10C).

Six weeks after the FC training, the same animals were once again tested in both contexts A and B for the recall of fear memories in adulthood (Table 3). Statistical analysis of freezing behavior revealed a lack of a significant effect of early-life treatment on CFC (*H*_3_ = 4.14, *p* = 0.246) and AFC memory recall in adult rats (*H*_3_ = 1.63, N_1_ = 14, N_2–4_ = 10, *p* = 0.653, Kruskal–Wallis test) (Online Resource ESM_4: Fig. S8A–B).

Seven days later, all animals underwent a session of retraining of FC in context A. Retraining followed the same procedure as training during preadolescence (Day 1 of experiment) (Table 3). On the following day, fear memory was tested both in contexts A and B (the same sessions as on Day 2 during preadolescence). One way ANOVA of freezing behavior during retraining of FC did not show any differences between experimental groups (*F*_3,40_ = 0.99, *p* = 0.406) (Fig. 10D). Interestingly, in our analysis of the CFC expression after retraining in adulthood we observed that MS rats showed increased freezing in response to context A compared to AFR rats (*F*_3,40_ = 3.28, *p* = 0.031, ANOVA followed by Tukey’s test) (Table S2) (Fig. 10E). Additionally, VEH injections significantly reduced freezing behavior in MS rats. The SAL-MS rats did not differ significantly from the MS rats (*p* = 0.115), VEH-MS (*p* = 0.979) or AFR rats (*p* = 0.996) in their CFC expression after retraining in adulthood (Tukey’s test) (Fig. 10E). Additionally, there was no significant effect of early-life treatment on the expression of AFC after retraining in adulthood (in context B) (*F*_3,40_ = 1.92, *p* = 0.141, ANOVA) (Fig. 10F).

### The effects of MS and early-life SAL/VEH injections on sucrose preference in preadolescent and adult rats

To study anhedonic-like behaviors, we first applied the sucrose preference test in preadolescent rats, and then, six weeks later, the same animals were retested for sucrose preference when they approached adulthood (PND 70).

**Fig. 11:**
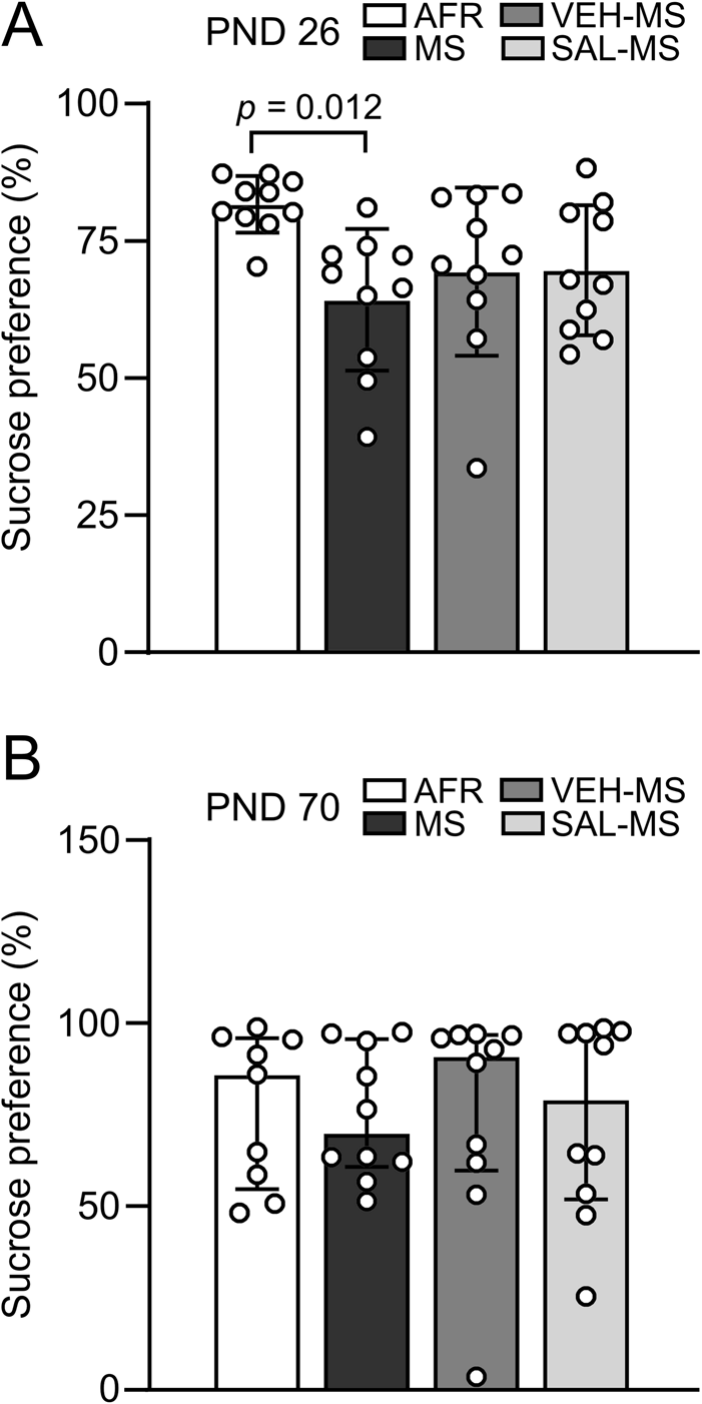
The effects of MS and early-life SAL/VEH injections on sucrose preference in preadolescent (**A**) and adult rats (**B**). The data are presented as the mean ± SD (**A**) or median and IQR (**B**) and were analyzed by one-way ANOVA or Kruskal–Wallis test, respectively (*n* = 10). Circles represent individual data points. Connector indicates statistically significant difference between specific experimental groups in Tukey’s HSD post hoc test. *AFR* animal facility rearing, *MS* maternal separation, *PND* postnatal day, *SAL* salubrinal, *VEH* vehicle

Statistical analysis of sucrose preference during the preadolescence period revealed a significant effect of early-life treatment (one-way ANOVA, *F*_3,36_ = 3.82, *p* = 0.018) (Online Resource ESM_1: Table S2). Specifically, MS rats showed reduced sucrose preference compared to AFR rats (Fig. 11A) (Tukey’s test). The behavior of VEH-MS and SAL-MS rats did not differ significantly from MS rats (for VEH-MS: *p* = 0.770; for SAL-MS: *p* = 0.743) or AFR rats (for VEH-MS: *p* = 0.117; for SAL-MS: *p* = 0.129, Tukey’s test) (Fig. 11A). There was no difference in sucrose preference between the treatment groups in adulthood (Kruskal–Wallis test, *H*_3_ = 0.21, N_1–4_ = 10, *p* = 0.996) (Fig. 11B).

### The effects of MS and early-life SAL/VEH injections on novelty- and amphetamine-induced locomotor activity in preadolescent and adult rats

**Fig. 12:**
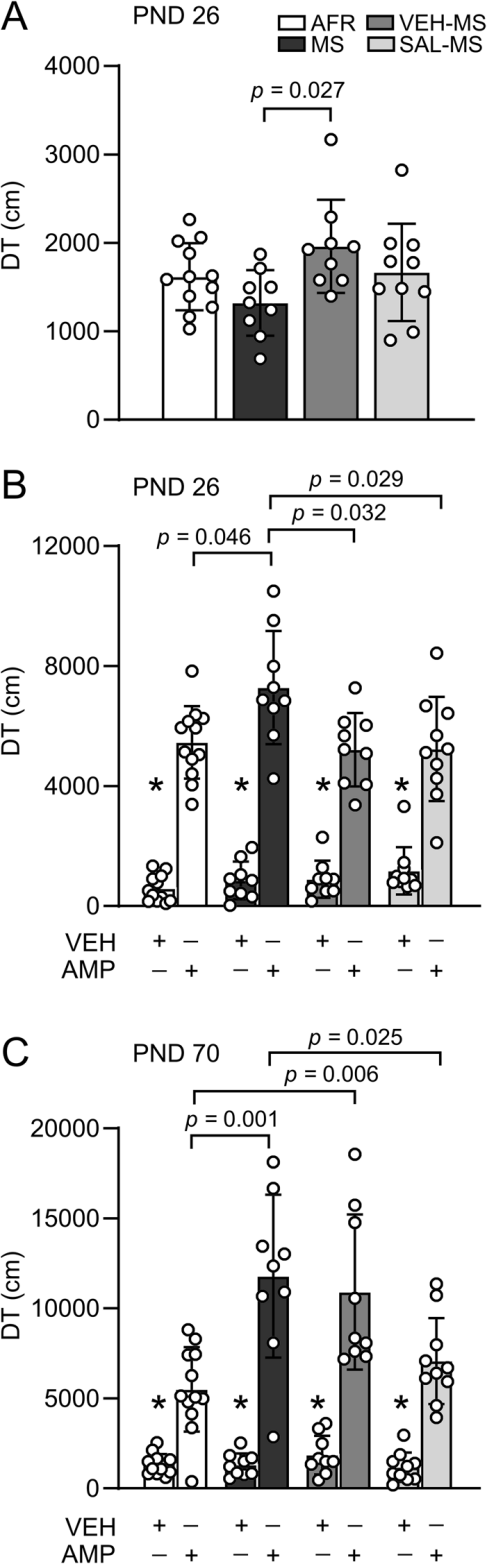
The effects of MS and early-life SAL/VEH injections on locomotor activity: novelty-induced locomotion in preadolescents (**A**), amphetamine (AMP)-induced locomotor activity in preadolescents (**B**) and adults (**C**). The data are presented as the mean ± SD (*n* = 10–14) and expressed as distance traveled during the specific session. Results were analyzed by mixed-design ANOVA. Circles represent individual data points. *p < 0.01 vs. AMP in corresponding early-life treatment group (Tukey’s HSD post hoc test). Connectors indicate other statistically significant differences between specific experimental groups in Tukey’s test. *AFR* animal facility rearing, *AMP* amphetamine, *DT* distance traveled, *MS* maternal separation, *PND* postnatal day, *SAL* salubrinal, *VEH* vehicle

The next goal of the study was to determine whether early-life treatment affected the locomotor activity of rats in response to novelty and amphetamine injections. Analysis of novelty-induced locomotion in preadolescent rats revealed that VEH-injected MS rats traveled a greater distance than MS rats (*F*_3,36_ = 2.91, *p* = 0.047, ANOVA followed by Tukey’s test) (Online Resource ESM_1: Table S2) (Fig. 12A). A mixed-design ANOVA of amphetamine-induced locomotion during preadolescence showed the significant effects of early-life treatment (*F*_3,36_ = 3.53, *p* = 0.024) and amphetamine injection (*F*_1,36_ = 338.20, *p* < 0.0001) on PND 26 and a significant interaction between these factors (*F*_3,36_ = 3.68, *p* = 0.021). In all experimental groups, amphetamine injection enhanced locomotor activity compared to VEH injection (Tukey’s test) (Fig. 12B). Moreover, MS rats showed greater amphetamine-induced locomotor activity than AFR rats, and this effect was prevented by early-life VEH and SAL treatment (Tukey’s test) (Fig. 12B).

After six weeks, the same groups of rats were retested for locomotor activity when they approached adulthood (PND 70) (Fig. 12C). A mixed-design ANOVA of amphetamine-induced locomotion during adulthood also revealed statistically significant effects of early-life treatment (*F*_3,36_ = 7.17, *p* = 0.0007) and amphetamine injection (*F*_1,36_ = 203.27, *p* < 0.0001) as well as a significant interaction between these factors (*F*_3,36_ = 7.96, *p* = 0.0003). In all experimental groups, amphetamine injection enhanced locomotor activity compared to VEH injection (Tukey’s test) (Fig. 12C). Additionally, MS and VEH-MS rats showed greater amphetamine-induced locomotion than AFR rats. Moreover, SAL injections reduced amphetamine-triggered locomotor activity in MS rats (Tukey’s test) (Fig. 12C).

### A search for a permanent imprint of MS and early-life SAL/VEH treatment on ER stress, the UPR and apoptosis in the mPFC

To determine whether MS and early-life SAL/VEH treatment left a permanent imprint on the expression of ER stress, UPR and apoptosis markers in the mPFC, we measured expression levels of relevant mRNA in adult rats.

Statistical analysis revealed that among ER stress and UPR markers, only the expression of Eif2a was significantly affected by early-life treatment (one-way ANOVA: *F*_3,20_ = 4.07, *p* = 0.021) (all results and statistics are presented in Table 6). Specifically, SAL-injected MS rats showed lower Eif2a mRNA levels than AFR rats. A similar trend was also observed in VEH-injected MS rats, though it was not statistically significant (*p* = 0.059) (Tukey’s test) (Table 6).

**Table 6:** The effects of MS and early-life SAL/VEH treatment on mRNA expression of ER stress, UPR and apoptosis markers in the mPFC of adult rats

Gene	Group	Relative mRNA level	Statistic
Hspa5	AFR	0.1622 ± 0.0071	*F*_3,20_ = 1.74, *p* = 0.190
	MS	0.1618 ± 0.0072	
	VEH-MS	0.1534 ± 0.0134	
	SAL-MS	0.1512 ± 0.0127	
Eif2ak3	AFR	0.0222 ± 0.0019	*F*_3,20_ = 3.00, *p* = 0.055
	MS	0.0206 ± 0.0012	
	VEH-MS	0.0211 ± 0.0016	
	SAL-MS	0.0231 ± 0.0014	
Ern1	AFR	0.00420 (0.0005)	*H*_3_ = 3.99, *p* = 0.262
	MS	0.00440 (0.0004)	
	VEH-MS	0.00404 (0.0004)	
	SAL-MS	0.00463 (0.0008)	
Atf6	AFR	0.0312 (0.0074)	*H*_3_ = 4.73, *p* = 0.193
	MS	0.0305 (0.0015)	
	VEH-MS	0.0307 (0.0022)	
	SAL-MS	0.0324 (0.0032)	
Eif2a	AFR	0.0116 ± 0.0010	***F***_**3,20**_** = 4.07, *p* = 0.021**
	MS	0.0106 ± 0.0009	
	VEH-MS	0.0091 ± 0.0013	
	SAL-MS	0.0088 ± 0.0025*	
Casp9	AFR	0.0161 ± 0.0011	***F***_**3,20**_** = 4.20, *p* = 0.019**
	MS	0.0175 ± 0.0009	
	VEH-MS	0.0181 ± 0.0018*	
	SAL-MS	0.0182 ± 0.0005*	
Casp3	AFR	0.00105 (0.0002)	*H*_3_ = 3.85, *p* = 0.278
	MS	0.00093 (0.0004)	
	VEH-MS	0.00093 (0.0001)	
	SAL-MS	0.00104 (0.0001)	
Casp12	AFR	0.00010 (0.00011)	*H*_3_ = 5.93, *p* = 0.115
	MS	0.00010 (0.00003)	
	VEH-MS	0.00008 (0.00002)	
	SAL-MS	0.00015 (0.00012)	
Bax	AFR	0.0506 (0.0028)	*H*_3_ = 7.43, *p* = 0.069
	MS	0.0474 (0.0018)	
	VEH-MS	0.0406 (0.0048)	
	SAL-MS	0.0463 (0.0040)	
Bcl2	AFR	0.0042 ± 0.0002	***F***_**3,20**_** = 4.71, *p* = 0.012**
	MS	0.0043 ± 0.0004	
	VEH-MS	0.0039 ± 0.0004	
	SAL-MS	0.0046 ± 0.0002^**#**^	

The mRNA expression was determined by RT-qPCR and presented as relative values of mRNA levels in arbitrary units. The data are presented as the mean ± SD or median (IQR), *n* = 6. Statistically significant effects are given in bold. *p < 0.05 vs. AFR, ^#^*p* < 0.05 vs. VEH-MS (Tukey’s HSD post hoc test). *AFR* animals facility rearing, *ER* endoplasmic reticulum, *IQR* interquartile range, *mPFC* medial prefrontal cortex, *MS* maternal separation, *SAL* salubrinal, *UPR* unfolded protein response, *VEH* vehicle

Analysis of the effect of early-life treatment on the expression of apoptotic markers showed statistical significance only in the case of caspase-9 (*F*_3,20_ = 4.20, *p* = 0.019) and Bcl2 mRNA expression (*F*_3,20_ = 4.71, *p* = 0.012, ANOVA) (Table 6). Specifically, both SAL- and VEH-injected MS rats had greater levels of caspase-9 mRNA compared to AFR rats (Tukey’s test). Additionally, SAL-MS rats showed increased Bcl2 expression compared with VEH-MS rats (Tukey’s test) (Table 6).

Finally, we analyzed the effect of early-life MS and SAL/VEH treatment on the number of neurons and glial cells in the mPFC of adult rats. Representative photomicrographs showing NeuN-IR neurons, GFAP-IR astrocytes and IBA1-IR microglial cells in the subregions of the mPFC of adult rats are presented in Online Resource ESM_8–10. Statistical analysis revealed that early-life treatment significantly affected the number of microglial cells but not the other populations of analyzed cells (all results and statistics are presented in Table 7). Notably, SAL-injected MS rats had a lower number of IBA1-IR microglial cells than MS and AFR rats in the PLC region (*F*_3,20_ = 4.01, *p* = 0.022, one-way ANOVA followed by Tukey’s test). Moreover, in the Cg1 region of the mPFC, SAL-MS rats also had a lower number of microglial cells than AFR rats (*F*_3,20_ = 3.47, *p* = 0.035, ANOVA followed by Tukey’s test) (Table 7).

**Table 7:** The effects of MS and early-life SAL/VEH treatment on the number of neurons astrocytes and microglial cells in the mPFC of adult rats

Cell marker	mPFC region	Group	Number of IR cells	Statistic
NeuN	Cg1	AFR	610,968.1 ± 29,926.0	*F*_3,20_ = 1.54, *p* = 0.236
		MS	573,292.1 ± 50,768.4	
		VEH-MS	573,422.1 ± 28,788.9	
		SAL-MS	585,200.9 ± 24,677.5	
	PLC	AFR	1,164,797.7 ± 77,242.8	*F*_3,20_ = 1.06, *p* = 0.386
		MS	1,148,378.2 ± 69,778.6	
		VEH-MS	1,123,820.5 ± 48,786.8	
		SAL-MS	1,100,882.2 ± 66,967.7	
	ILC	AFR	225,567.6 ± 11,134.5	*F*_3,20_ = 0.94, *p* = 0.442
		MS	221,745.4 ± 13,552.3	
		VEH-MS	215,989.6 ± 16,123.5	
		SAL-MS	214,057.7 ± 12,151.9	
GFAP	Cg1	AFR	173,806.4 (22,206.7)	*H*_3_ = 6.31, *p* = 0.097
		MS	175,147.5 (57,261.2)	
		VEH-MS	208,999.4 (50,311.9)	
		SAL-MS	177,485.1 (22,710.1)	
	PLC	AFR	350,950.1 (46,224.9)	*H*_3_ = 3.39, *p* = 0.335
		MS	387,533.7 (126,461.1)	
		VEH-MS	379,827.4 (77,912.9)	
		SAL-MS	373,984.3 (33,948.9)	
	ILC	AFR	104,831.6 ± 10,104.6	*F*_3,20_ = 2.88, *p* = 0.061
		MS	118,909.7 ± 18,272.5	
		VEH-MS	124,738.9 ± 13,458.0	
		SAL-MS	107,309.7 ± 11,342.5	
IBA1	Cg1	AFR	103,190.2 ± 5918.0	***F***_**3,20**_** = 3.47, *p* = 0.035**
		MS	98,219.3 ± 7121.3	
		VEH-MS	94,696.7 ± 9686.7	
		SAL-MS	88,517.6 ± 9156.4*	
	PLC	AFR	175,265.5 ± 7211.8	***F***_**3,20**_** = 4.01, *p* = 0.022**
		MS	173,557.7 ± 16,899.1	
		VEH-MS	165,775.0 ± 12,457.6	
		SAL-MS	154,006.0 ± 8281.9*^#^	
	ILC	AFR	40,693.6 ± 3630.7	*F*_3,20_ = 3.06, *p* = 0.052
		MS	40,061.2 ± 3470.1	
		VEH-MS	37,174.0 ± 4103.3	
		SAL-MS	35,219.0 ± 3023.6	

Data indicate the numbers of IR cells per region estimated by stereological method (the mean ± SD or median (IQR), *n* = 6). Statistically significant effects are given in bold. ^*^p < 0.05 vs. AFR, ^#^*p* < 0.05 vs. MS (Tukey’s HSD post hoc test). *AFR* animal facility rearing, *Cg1* cingulate cortex, *ILC* infralimbic cortex, *IQR* interquartile range, *IR* immunoreactive, *mPFC* medial prefrontal cortex, *MS* maternal separation, *PLC* prelimbic cortex, *PND* postnatal day, *SAL* salubrinal, *VEH* vehicle

## Discussion

The main goal of the present study was to investigate whether ER stress and UPR processes are affected by the MS procedure and thereby underlie the cellular and behavioral consequences of ELS. We found that MS enhanced the activation of the UPR in juveniles to a small degree and modulated the mRNA expression of a few apoptotic markers in the mPFC of juveniles and preadolescents but not in adults. However, MS did not affect the numbers of neurons or glial cells in the mPFC at any age. Both early-life SAL and VEH injections (often in a treatment-specific manner) affected the expression of UPR and apoptotic markers, especially in juvenile and preadolescent MS rats, and in some cases prevented MS-induced effects at the biochemical level. Moreover, SAL/VEH generally mitigated the behavioral effects of MS.

### The effects of MS procedure on ER stress and UPR processes and apoptosis in the mPFC

Enhanced expression and activation of ER stress and UPR markers have been observed in animal models of depression based on chronic stress procedures in adults [27–29] and in different acute stress models [53, 54]. Moreover, the infusion of tunicamycin, an activator of the UPR, into the hippocampus produces a depressive-like phenotype in rats [55]. We have recently shown that MS produces long-lasting upregulation of chaperones HSPA5 and HSPA1B in the brain and blood, which suggests that ELS may influence ER stress and UPR processes throughout development [30]. To the best of our knowledge, the present study is the first to comprehensively examine the role of ER stress and UPR processes in ELS-induced effects. We once again confirmed that MS increased the protein expression of HSPA5 in the mPFC of juveniles on PND 15, which is 24 h after the last MS. MS also increased the phosphorylation (activation) of one of the ER stress sensors, IRE1α, in juveniles. These effects were temporal, and we generally did not observe any MS-induced changes in the expression of ER stress or UPR markers in the mPFC of preadolescents and adults. Concurrently, in this study, we observed subtle changes in the mRNA expression of apoptotic markers in MS juveniles and preadolescents but not in adults. Specifically, the expression of Casp12 was increased in juveniles and decreased in preadolescent MS rats. Casp12 is a specific caspase that is localized in the ER membrane and engaged in the ER stress-induced pathway of apoptosis [56]. We also observed an MS-induced increase in the mRNA expression of Bcl2 in preadolescents. These subtle changes in the expression of UPR and apoptotic markers were not accompanied by any changes in the numbers of neurons, astrocytes or microglial cells in the mPFC of preadolescent or adult MS rats. These results are in contrast to our previous observations in adolescent rats (on PND 35), in which we found a clear delay in neurodevelopmental apoptosis, manifested as increased numbers of neurons in the mPFC and antiapoptotic trends in the expression and activation of apoptotic markers [10]. Thus, our results suggest that adolescence is a developmental period that specifically unveils the effects of ELS on neurodevelopmental apoptosis of the mPFC [10, 37]. This is not surprising, because during adolescence, the mPFC undergoes intensive structural and functional reorganization [32, 34]. Nevertheless, we expected that the effects of MS would be manifested even earlier, in preadolescence period, but our biochemical results did not support that hypothesis. However, in the present study we did observe some behavioral effects of MS in the preadolescence period, such as a decrease in sucrose preference (anhedonia-like behavior) and an impairment in CFC expression. Moreover, MS-triggered enhancement of the locomotor response to psychostimulant drugs, a typical behavioral phenotype observed in ELS models [39, 57, 58], was also observed in preadolescents. This behavioral effect was the only enduring effect observed also in adult MS rats. Additionally, MS rats showed an increased CFC expression after retraining in adulthood. It is worth noting that the mPFC is highly implicated both in the expression of FC [59] and psychostimulant-induced hyperlocomotion [60]. Taken together, the results showed that, although we did not detect a strong impact of MS on ER stress, the UPR and apoptosis in the mPFC of juveniles and preadolescents, MS did produce long-lasting functional consequences observed at behavioral level even during preadolescence period. Our study implicates that MS procedure may influence ER stress and the UPR in an age-specific manner and manifest its strongest effects on the abovementioned processes in different developmental time points than that chosen for the present experiment. However, we should bear in mind that the studied behaviors are also regulated by many other cellular mechanisms and brain regions.

### The effects of early-life modulation of ER stress and the UPR by SAL/VEH treatment on biochemical and behavioral phenotype of MS rats

To modulate ER stress and UPR processes in MS rats, we applied repeated early-life treatment with SAL before each MS procedure. SAL is a small-molecule inhibitor of eIF2α phosphatases that prolongs eIF2α phosphorylation at residue S51 and thereby its inactivation, which causes an inhibition of general protein synthesis. SAL has been shown to reduce cell death and have neuroprotective properties in animal models of neurodegenerative disorders [13, 61], cerebral ischemia [43] and traumatic brain injury [42, 62]. In the present study, we also used conventional solvent/vehicle injections (VEH) to adequately control experimental conditions. Interestingly, early-life VEH treatment by itself affected the studied parameters and sometimes produced similar effects to SAL treatment. This phenomenon greatly complicated understanding and interpretation of the results. Both VEH and SAL treatments prevented some MS-induced effects. For example, SAL treatment decreased Bcl2 mRNA levels in preadolescent rats and dampened amphetamine-induced hyperlocomotion in preadolescents and adults (SAL-MS vs. MS rats). VEH injections prevented the effects of MS at the level of Casp12 and Bcl2 transcription in juveniles and preadolescents, respectively. At behavioral level, VEH treatment also reduced amphetamine-triggered locomotor activity in preadolescent MS rats and CFC expression in MS adult rats (VEH-MS vs. MS rats). In many cases, when MS rats resembled AFR rats at the biochemical level, SAL or VEH significantly modulated the mRNA expression of ER stress, UPR and apoptosis markers especially in juvenile and preadolescent MS rats. For example, SAL- and/or VEH-MS rats generally showed reduced mRNA expression of many ER stress and UPR markers when compared to MS rats. The results suggest that both VEH and SAL treatment exerted inhibitory influence on ER stress and UPR processes in MS rats.

At the behavioral level, in sucrose preference or CFC tests, SAL- and VEH-MS preadolescent rats exhibited intermediate behavioral phenotypes between AFR and MS rats (not significantly different from either AFR and MS rats), and those phenotypes turned out to be advantageous in the specific experimental conditions of this study.

The effect of SAL/VEH on anxiety-like behaviors in the light/dark box test is also worth noting. Interestingly, preadolescent SAL-MS and VEH-MS rats were less anxious than AFR rats. On the other hand, in adulthood, SAL and VEH normalized the behavior of MS rats, which showed a statistically insignificant trend toward less fearful (impulsive-like) behavior. However, when a single comparison between the AFR and MS groups was performed, the analysis revealed that MS significantly enhanced impulsive-like behavior in adults, which is in line with our previous studies [8, 30]. Interestingly, Logstdon et al. reported that SAL treatment reduced impulsive-like behaviors in adult rats subjected to traumatic brain injury [42, 62]. Whereas, Jangra et al. showed that other ER stress inhibitor, the chemical chaperone sodium phenylbutyrate, abrogated anxiety- and depressive-like behaviors in adult mice subjected to chronic restraint stress [27]. It is worth emphasizing that, in contrast to the abovementioned studies, we injected SAL/VEH during the early-life period, and the behavioral consequences of that treatment were observed at later developmental stages, even in adulthood.

Our results concerning SAL/VEH action in our experimental paradigm lead to the question of whether SAL treatment exerted a specific biological effect as an inhibitor of eIF2α dephosphorylation, promoting the inhibition of global translation. Some data evidently supported the specific action of SAL in our experiment. Namely, 24 h after the last separation in juveniles, the mRNA and protein expression of eIF2α was significantly lower in SAL-MS rats than in MS rats. These results may indicate that some kind of compensation or adaptation to repeated SAL injections and to an inhibition of eIF2α activity occurred. Interestingly, in adulthood, SAL-MS rats still showed low levels of Eif2a expression, though this effect was statistically significant only when compared to AFR rats. Although we did not observe an increase in eIF2α phosphorylation after SAL treatment in MS juveniles, this result was not surprising because MS by itself did not induce activation/phosphorylation of PERK or eIF2α. However, it is important to note that PERK is not the only kinase that phosphorylates eIF2α [63]. Additionally, although eIF2α phosphorylation attenuates general translation, it simultaneously promotes translation of specific proteins, such as activating transcription factor 4 (ATF4). ATF4 is known to activate the transcription of the regulatory subunit of protein phosphatase 1, also known as growth arrest and DNA damage-inducible protein GADD34, to generate active eIF2α phosphatase and initiate a feedback loop to dephosphorylate eIF2α and consequently restore general protein synthesis. This feedback loop in the regulation of eIF2α phosphorylation is the main concern associated with the use of SAL and other eIF2α phosphatase inhibitors in the clinic and in animal models because it limits the duration of their action [13]. Searching for another evidence for the specific action of SAL, it is worth noting a decrease in the number of microglial cells in the PLC of SAL-MS adult rats compared to MS and AFR rats. This interesting observation needs further studies. It is well known that ER stress and the UPR are key regulators of inflammation and function of immune cells in the periphery and brain [11, 14, 15]. Early-life SAL treatment could potentially influence the rate of postnatal proliferation and/or apoptosis of microglial cells.

### Enduring biological action of early-life VEH injections: a pitfall and challenge for controlling of experimental conditions

It is well known that routine laboratory procedures such as animal handling and injections involve some level of mild to moderate physical and psychological stress. Acute procedures usually activate the hypothalamic–pituitary–adrenal axis and increase the levels of glucocorticoids, whereas chronic interventions lead to a desensitization of this response with time [64–66]. Therefore, in pharmacological studies, solvent/vehicle injections are commonly used to control experimental conditions. However, growing amount of data has accumulated and shown that both acute and chronic VEH injections not only modulate serum glucocorticoids levels but also affect animal behavior [64, 65, 67]. For example, single *ip* injections of saline produced anxiogenic effect in mice [66]. A recent study also demonstrated that repeated saline injections for 6 weeks (starting during the adolescent period) increased anxiety-like behaviors, decreased systemic inflammation, and increased corticosterone reactivity and microglial activation in the dentate gyrus of the hippocampus [67]. However, when saline treatment was combined with additional stress (social isolation), it did not worsen and even improved some effects produced by chronic stress [67]. A similar trend was observed in our study. It has been argued that exposure to moderate but not minimal or substantial amounts of stressors, especially during the perinatal period, may facilitate coping with other environmental challenges later in life. This phenomenon is known as stress inoculation [68, 69]. In the case of our study, we have a combination of two early-life stressors (MS and VEH/SAL injections) that turned out to be beneficial for MS rats and produced a more resilient phenotype in preadolescents and adults. Our study suggests that a modulation of ER stress and UPR processes may underlie the injection-triggered changes in animal behavior, especially when injections are applied during a critical period of early-life development. However, it is worth underlining that we studied the effects of VEH treatment only in stressed subjects and not in control (AFR) animals.

We cannot also overlook in our discussion a potential biological action of the solvent/vehicle used in our experiments, 2.5% DMSO diluted in PBS and given in a dose of 0.125 µl of DMSO per gram of body weight. We chose DMSO as a vehicle for SAL based on a large amount of previous data in the literature [42, 43, 70]. DMSO is routinely used in biological research as a solvent and a cryopreservative in bone marrow and organ transplants. However, data have accumulated showing that DMSO may produce both adverse and beneficial effects on brain tissue [71–75]. A small dose of 0.2 µl/g given for 5 days to juvenile rats has been shown to lead to global changes in the brain metabolome and increase oxidative stress and proteolysis markers. However, only higher doses of DMSO (2 and 4 µl/g) have been shown to affect rat behavior, i.e., decreased social habits [75]. Another group demonstrated that DMSO (0.3–10 µl/g) produced widespread apoptosis in the developing brain [73]. Nevertheless, in the above-cited studies, DMSO was injected in undiluted form (~ 100%) and at higher doses than our VEH treatment. On the other hand, DMSO has also been shown to have neuroprotective and procognitive properties in animal models of ischemia, cerebral hypoperfusion and Alzheimer’s disease [71, 72, 74]. It has been argued that the neuroprotective effects of DMSO may be linked to its anti-inflammatory and free radical scavenging activities [72, 76]. To the best of our knowledge, only one study showed that DMSO modulated (increased) the expression of UPR genes, including Hspa5, though in mouse embryos and in the context of the cytotoxic effects of DMSO [77].

Unfortunately, based on the results presented in our study, we cannot explicitly determine whether the effects produced by VEH/SAL treatments in MS rats are specifically related to the action of SAL or DMSO or the procedure of repeated injections during the early-life period. The results show how unpredictable the effects of repeated VEH injections can be in the early-life period. However, we can at least state that early-life VEH/SAL treatment modulated ER stress and UPR processes in MS rats to some extent. Although, SAL/VEH treatment did not leave a permanent imprint on the expression of ER stress and UPR markers in the mPFC of MS adults, it promoted resilience at the behavioral level in both preadolescent and adult rats.

Recently, it has been argued that ER stress and the UPR, next to oxidative stress and hormonal regulation, may play a role in mediating inter- and intraspecific variations in response to different environmental conditions and, in this way, may shape susceptibility or resilience to stressors [78]. ER stress and the UPR are evolutionarily conserved and heritable cellular processes. There are also considerable individual variations in ER stress and UPR phenotypes in humans and nonhuman animals, suggesting that these phenotypes can be subjected to natural selection [78, 79]. Strong individual variations in ER stress and UPR phenotypes may be, to some degree, responsible for the relatively small changes in the expression of ER stress and UPR markers observed in our experimental paradigm, which involved Wistar outbred rats. Nevertheless, these small changes may better reflect the situation in naturally existing populations.

The main limitation of the present study was that we did not include female subjects in the whole experiment and analysis and not explore sex differences in ER stress and UPR signaling. However, our pilot study of the mRNA expression of UPR and apoptotic markers showed that females were less affected by MS procedure and early-life SAL/VEH treatment than males. It is generally in line with our previous reports showing that in many aspects female rats are more resilient to MS procedure conducted in our laboratory [8, 9, 40].

### The role of ER stress and UPR signaling in the pathophysiology of ELS-related diseases: potential clinical implications

Epidemiological and clinical studies clearly show that ELS not only increases the risk of mental disorders but also physical health problems, such as metabolic syndrome that may lead to cardiovascular diseases and type 2 diabetes [80, 81]. Recently, broadscale attempts to identify causative mechanisms linking ELS to psycho-cardio-metabolic multimorbidity have been started [82]. We hypothesize that ER stress and UPR processes may potentially represent shared molecular pathways and mechanisms by which ELS affects both mental and physical health. ER stress and UPR signaling acts in most cells and tissues and has been implicated in the pathophysiology of numerous diseases, such as cancer, diabetes, atherosclerosis, neurodegenerative diseases [15, 16, 83], as well as MDD and BD [17–22]. Interestingly, all the above mentioned diseases have evident inflammatory components and ER stress and UPR processes are known to regulate inflammatory response [11, 14, 15, 84–86]. In the present study we applied a systemic SAL administration, therefore this inhibitor of ER stress could potentially affect not only the brain but also other organs and systems. Further multiorgan studies are needed to confirm the hypothesis that ER stress and UPR signaling can be implicated in the pathophysiology of ELS-related diseases or the phenomenon of resilience.

Many pharmacological strategies targeting different components of UPR signaling for disease intervention have been tested in preclinical and clinical studies [12, 87]. They include chemical chaperones and small-molecule activators or inhibitors of the UPR, such as SAL [13, 87] The most promising strategies concern the treatment of cancer and cardiovascular and neurodegenerative disorders [87, 88]. However, UPR-targeting drugs are non-selective and their potential administration to the patients with a history of ELS and multimorbidity is rather unlikely. Nevertheless, the key players in ER stress and the UPR can be at least potential candidate biomarkers of ELS-related changes, measured in blood or peripheral organ biopsy samples and help to diagnose ELS-induced multimorbidity [12]. UPR biomarkers have been already used to monitor progression of cancer, kidney disease, neurodegenerative diseases [12, 89]. It is worth mentioning that we previously showed that MS procedure caused enduring upregulation of Hspa5 expression in the blood that was accompanied by impulsive- and depressive-like behavior in male adult rats [30].

## Conclusions

We found that MS did not exert a strong impact on ER stress and UPR processes or apoptosis at developmental stages under study. However, both early-life SAL and VEH treatment (often in an injection-specific manner) influenced the expression of UPR and apoptotic markers, especially in juvenile and preadolescent MS rats, and in some cases prevented MS-induced effects at the biochemical level. Moreover, SAL and/or VEH alleviated some behavioral effects of MS in both preadolescent and adult rats. These results suggest that a regulation of ER stress and UPR processes may play a potential role in the mechanisms of susceptibility or resilience to ELS and other environmental factors. Further multiorgan studies are needed to validate this interesting hypothesis in future.

## Supplementary Information

Below is the link to the electronic supplementary material.

Supplementary file1 (PDF 493 KB)

Supplementary file2 (PDF 4195 KB)

Supplementary file3 (PDF 6720 KB)

Supplementary file4 (PDF 1219 KB)

Supplementary file5 (PDF 12705 KB)

Supplementary file6 (PDF 13772 KB)

Supplementary file7 (PDF 13115 KB)

Supplementary file8 (PDF 13115 KB)

Supplementary file9 (PDF 13264 KB)

Supplementary file10 (PDF 13173 KB)

Supplementary file11 (PDF 10308 KB)

Supplementary file12 (PDF 2971 KB)

Supplementary file13 (PDF 6188 KB)

Supplementary file14 (PDF 3250 KB)

## Data Availability

The datasets generated during and/or analyzed during the current study are available from the corresponding author on reasonable request.

## Abbreviations

AFC: Auditory fear conditioning
AFR: Animal facility rearing
ATF6: Activating transcription factor 6
BD: Bipolar disorder
CFC: Contextual fear conditioning
Cg1: Cingulate cortex 1
eIF2α: Eukaryotic translation initiation factor 2α
Eif2ak3: Eukaryotic translation initiation factor 2 α kinase 3
alias: PERK: RNA-activated protein kinase (PRK)-like endoplasmic reticulum kinase
ELS: Early-life stress
ER: Endoplasmic reticulum
Ern1: Endoplasmic reticulum to nucleus signaling 1
alias: IRE1α: Inositol-requiring enzyme 1
FC: Fear conditioning
GFAP: Glial fibrillary acidic protein
Hspa5: Heat shock protein family A member 5
alias: GRP78: Glucose-regulated protein 78 kDa
IBA1: Ionized calcium-binding adapter molecule
ILC: Infralimbic cortex
IR: Immunoreactive
MDD: Major depressive disorder
mPFC: Medial prefrontal cortex
MS: Maternal separation
PLC: Prelimbic cortex
PND: Postnatal day
SAL: Salubrinal
SAL-MS: MS with salubrinal injections
SD: Standard deviation
UPR: Unfolded protein response
VEH-MS: MS with vehicle injections
