# Supplementary material for: Modulation of the endoplasmic reticulum stress and unfolded protein response mitigates the behavioral effects of early-life stress
Source: Pharmacol Rep. 2023 Feb 27;75(2):293–319. doi: 10.1007/s43440-023-00456-6 (PMC10060333; doi:10.1007/s43440-023-00456-6)
Supplement: Supplementary file 1 — Supplementary file1 (PDF 493 KB) [file 43440_2023_456_MOESM1_ESM.pdf]

Table S1. Results of one-way ANOVA or Kruskal-Wallis test investigating the effects of MS and early-life SAL/VEH treatment on mRNA and protein expression of ER stress, UPR and apoptosis markers in the mPFC of juvenile and preadolescent rats

**Parameter**	**PND 15**<br>**Statistic**	**PND 26**<br>**Statistic**
Hspa5 mRNA	**H_3 = 17.02, p = 0.0007**	**$F_{3,20}$ = 11.23, p < 0.0001**
HSPA5 protein	**$F_{3,20}$ = 3.35, p = 0.039**	H_3 = 0.99, p = 0.803
Eif2ak3 mRNA	$F_{3,20}$ = 1.78, p = 0.183	$F_{3,20}$ = 1.51, p = 0.242
PERK protein	$F_{3,20}$ = 1.47, p = 0.253	$F_{3,20}$ = 0.26, p = 0.850
p-PERK (Thr980) protein	**$F_{3,20}$ = 5.93, p = 0.005**	H_3 = 0.67, p = 0.879
Ern1 mRNA	$F_{3,20}$ = 1.70, p = 0.200	**$F_{3,20}$ = 11.15, p = 0.0002**
IRE1α protein	$F_{3,20}$ = 0.52, p = 0.672	H_3 = 3.18, p = 0.365
p-IRE1α (S724) protein	**$F_{3,20}$ = 3.88, p = 0.024**	$F_{3,20}$ = 2.88, p = 0.061
Atf6 mRNA	$F_{3,20}$ = 1.49, p = 0.248	**$F_{3,20}$ = 4.42, p = 0.015**
ATF protein	$F_{3,20}$ = 1.57, p = 0.229	$F_{3,20}$ = 0.90, p = 0.459
Eif2a mRNA	**H_3 = 18.83, p = 0.003**	H_3 = 1.67, p = 0.643
eIF2α protein	**$F_{3,20}$ = 6.10, p = 0.004**	$F_{3,20}$ = 1.10, p = 0.373
p-eIF2α (S51) protein	$F_{3,20}$ = 4.97, p = 0.050	H_3 = 1.63, p = 0.652
Casp9 mRNA	**$F_{3,20}$ = 5.11, p = 0.009**	H_3 = 6.76, p = 0.080
Caspase-9 protein	$F_{3,20}$ = 1.59, p = 0.222	$F_{3,20}$ = 0.56, p = 0.647
Cleaved caspase-9 protein	$F_{3,20}$ = 2.09, p = 0.135	$F_{3,20}$ = 0.49, p = 0.693
Casp3 mRNA	H_3 = 7.13, p = 0.068	**H_3 = 10.82, p = 0.013**
Cleaved caspase-3 protein	$F_{3,20}$ = 1.06, p = 0.389	H_3 = 9.53, p = 0.0500
Casp12 mRNA	**$F_{3,20}$ = 5.36, p = 0.007**	**$F_{3,20}$ = 5.26, p = 0.008**
Caspase-12 protein	$F_{3,20}$ = 0.62, p = 0.061	$F_{3,20}$ = 0.47, p = 0.708
Cleaved caspase-12 protein	$F_{3,20}$ = 0.52, p = 0.670	$F_{3,20}$ = 0.36, p = 0.785
Bax mRNA	**$F_{3,20}$ = 15.34, p < 0.0001**	H_3 = 1.32, p = 0.724
Bax protein	$F_{3,20}$ = 2.72, p = 0.072	$F_{3,20}$ = 1.79, p = 0.189
Bcl2 mRNA	$F_{3,20}$ = 2.67, p = 0.075	**$F_{3,20}$ = 12.02, p = 0.0001**
Bcl2 protein	$F_{3,20}$ = 1.60, p = 0.221	$F_{3,20}$ = 1.91, p = 0.160
Bax/Bcl2 mRNA	**$F_{3,20}$ = 11.29, p < 0.0001**	**H_3 = 16.61, p = 0.0009**
Bax/Bcl2 protein	$F_{3,20}$ = 0.51, p = 0.679	**H_3 = 11.29, p = 0.010**

Statistically significant differences are given in bold. *Abbreviations:* mPFC, medial prefrontal cortex; MS, maternal separation; PND, postnatal day; SAL, salubrinal; VEH, vehicle.

Table S2. Results of ANOVA or Kruskal-Wallis test investigating the effects of MS and early-life SAL/VEH treatment on behavioral phenotype of preadolescent and adult rats

**Parameter**	**PND 26**<br>**Statistic**	**PND 70**<br>**Statistic**
Light/dark box:		
% Time in light	$\boldsymbol{H_3 = 11.97, p = 0.007}$	$\boldsymbol{F_{3,45} = 4.51, p = 0.007}$
% Distance traveled in light	$\boldsymbol{H_3 = 12.87, p = 0.005}$	$\boldsymbol{H_3 = 12.83, p = 0.005}$
Transitions (N)	$\boldsymbol{H_3 = 8.58, p = 0.035}$	$H_3 = 6.0, p = 0.112$
Fear memory:		
FC acquisition	$F_{3,40} = 1.82, p = 0.160$	$F_{3,40} = 0.99, p = 0.406$
CFC expression	$\boldsymbol{F_{3,40} = 4.20, p = 0.011}$	$\boldsymbol{F_{3,40} = 3.28, p = 0.031}$
AFC expression	$H_3 = 2.91, p = 0.405$	$F_{3,40} = 1.92, p = 0.141$
CFC recall	N.A.	$H_3 = 4.14, p = 0.246$
AFC recall	N.A.	$H_3 = 1.63, p = 0.653$
Sucrose preference	$\boldsymbol{F_{3,36} = 3.82, p = 0.018}$	$H_3 = 0.21, p = 0.996$
Novelty-induced locomotion	$\boldsymbol{F_{3,36} = 2.91, p = 0.047}$	N/A
Amphetamine-induced locomotion:		
Effect of early-life treatment	$\boldsymbol{F_{3,36} = 3.53, p = 0.024}$	$\boldsymbol{F_{3,36} = 7.17, p = 0.0007}$
Effect of amphetamine	$\boldsymbol{F_{1,36} = 338.20, p < 0.0001}$	$\boldsymbol{F_{1,36} = 203.27, p < 0.0001}$
Effect of early-life x amphetamine	$\boldsymbol{F_{3,36} = 3.68, p = 0.021}$	$\boldsymbol{F_{3,36} = 7.96, p = 0.0003}$

Statistically significant differences are given in bold. *Abbreviations:* AFC, auditory fear conditioning; CFC, contextual fear conditioning; FC, fear conditioning; MS, maternal separation; N/A; not assessed; PND, postnatal day; SAL, salubrinal; VEH, vehicle.
