# Supplementary material for: Modulation of the endoplasmic reticulum stress and unfolded protein response mitigates the behavioral effects of early-life stress
Source: Pharmacol Rep. 2023 Feb 27;75(2):293–319. doi: 10.1007/s43440-023-00456-6 (PMC10060333; doi:10.1007/s43440-023-00456-6)
Supplement: Supplementary file 2 — Supplementary file2 (PDF 4195 KB) [file 43440_2023_456_MOESM2_ESM.pdf]

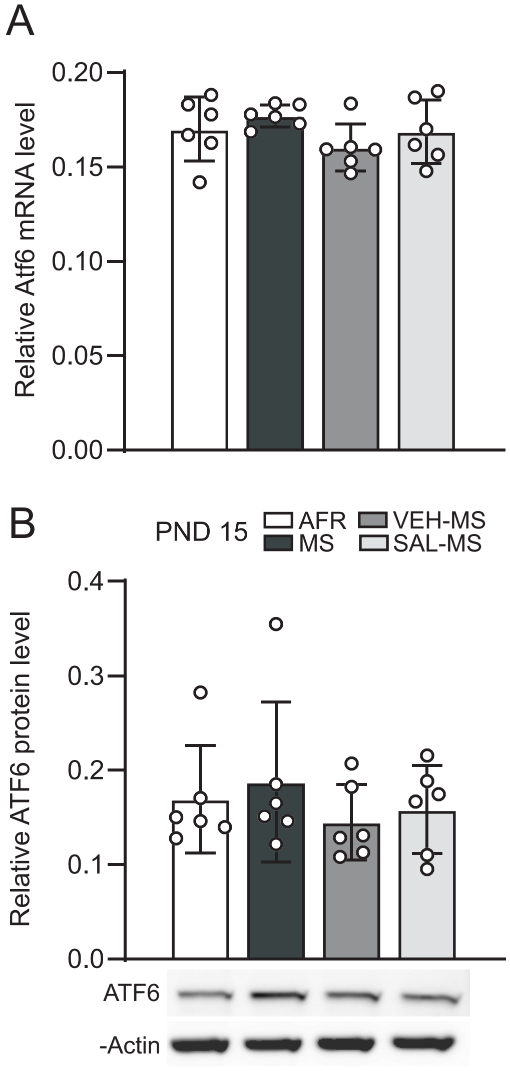

Fig. S1. The effects of MS and early-life SAL/VEH injections on mRNA (A) and protein expression (B) of ER stress sensor ATF6 in the mPFC of juvenile rats. The data are presented as the mean ± SD (n = 6) and were analyzed by one-way ANOVA. Circles represent individual data points. Statistical analysis showed no significant differences between experimental groups. *Abbreviations:* AFR, animal facility rearing; MS, maternal separation; PND, postnatal day; SAL, salubrinal; VEH, vehicle.

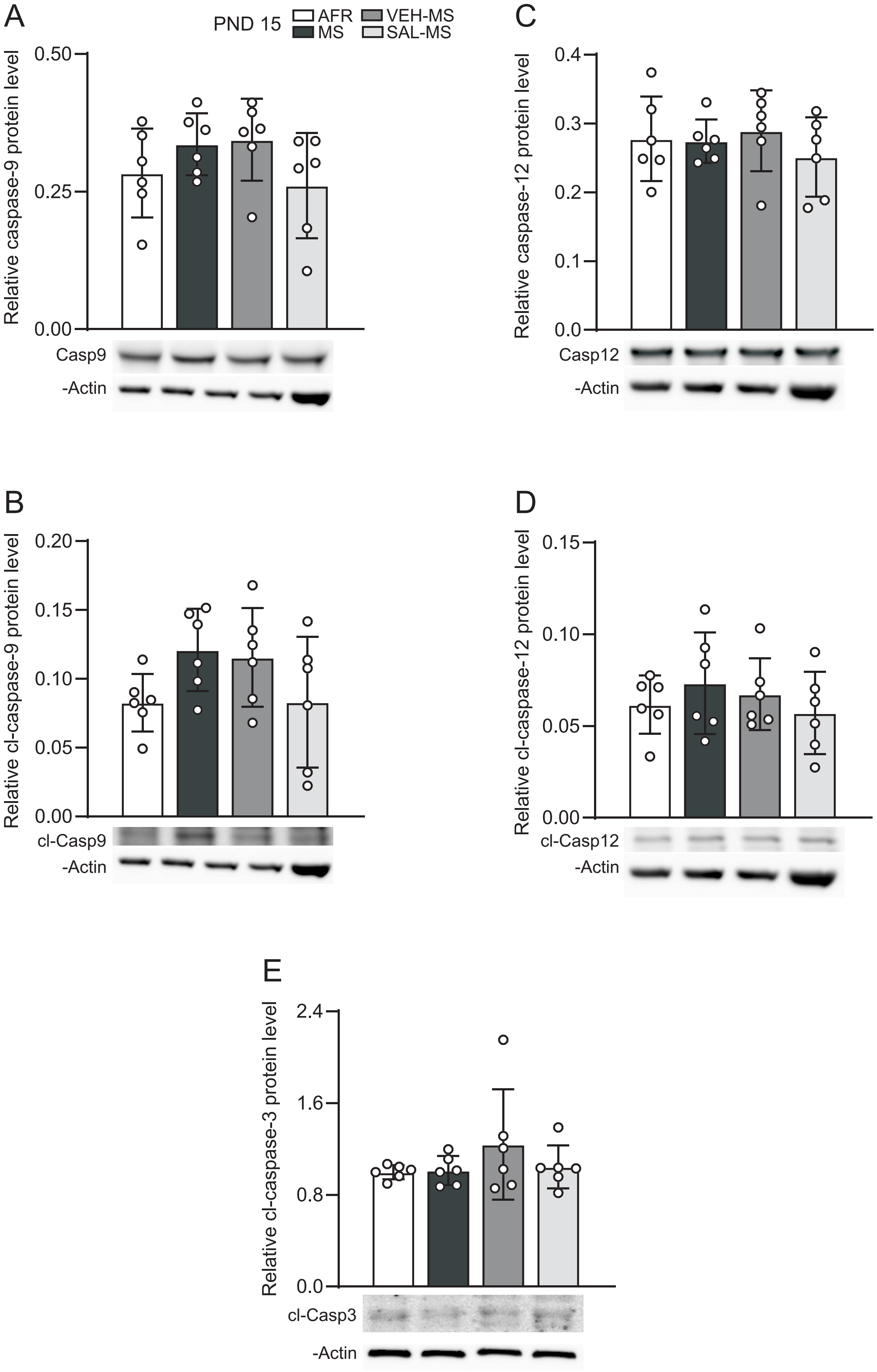

Fig. S2. The effects of MS and early-life SAL/VEH injections on protein expression and cleavage of caspase-9 (A-B), caspase-12 (C-D) and cleavage of caspase-3 (E) in the mPFC of juvenile rats. The data are presented as the mean ± SD (n = 6) and were analyzed by one-way ANOVA. Circles represent individual data points. Statistical analysis showed no significant differences between experimental groups. *Abbreviations:* AFR, animal facility rearing; cl, cleaved; MS, maternal separation; PND, postnatal day; SAL, salubrinal; VEH, vehicle.

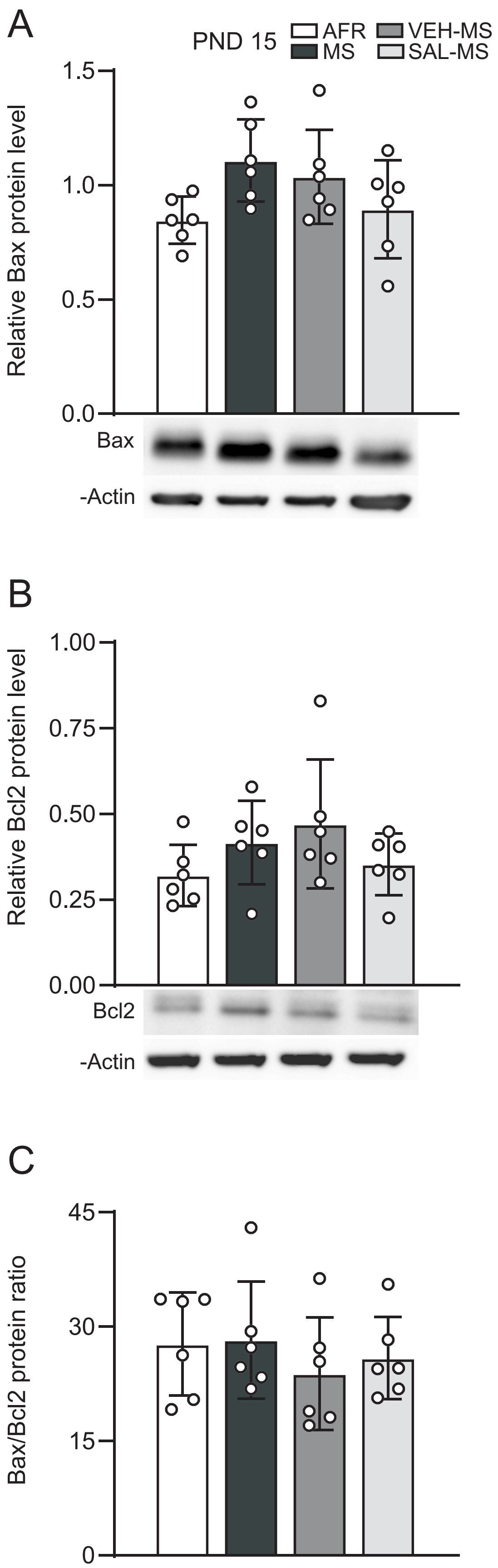

Fig. S3. The effects of MS and early-life SAL/VEH injections on Bax (A) and Bcl2 (B) protein levels and Bax/Bcl2 protein ratio (C) in the mPFC of juvenile rats. The data are presented as the mean ± SD (*n* = 6) and were analyzed by one-way ANOVA. Circles represent individual data points. Statistical analysis showed no significant differences between experimental groups. The same immunoblot of -Actin was used for normalization of both Bcl2 (B) and ATF6 immunoblots (Fig. S1B). After protein electrotransfer the blots were horizontally cut into appropriate pieces to separately evaluate ATF, -Actin and Bcl2 protein levels from the same samples. *Abbreviations:* AFR, animal facility rearing; MS, maternal separation; PND, postnatal day; SAL, salubrinal; VEH, vehicle.
