# Supplementary material for: Modulation of the endoplasmic reticulum stress and unfolded protein response mitigates the behavioral effects of early-life stress
Source: Pharmacol Rep. 2023 Feb 27;75(2):293–319. doi: 10.1007/s43440-023-00456-6 (PMC10060333; doi:10.1007/s43440-023-00456-6)
Supplement: Supplementary file 3 — Supplementary file3 (PDF 6720 KB) [file 43440_2023_456_MOESM3_ESM.pdf]

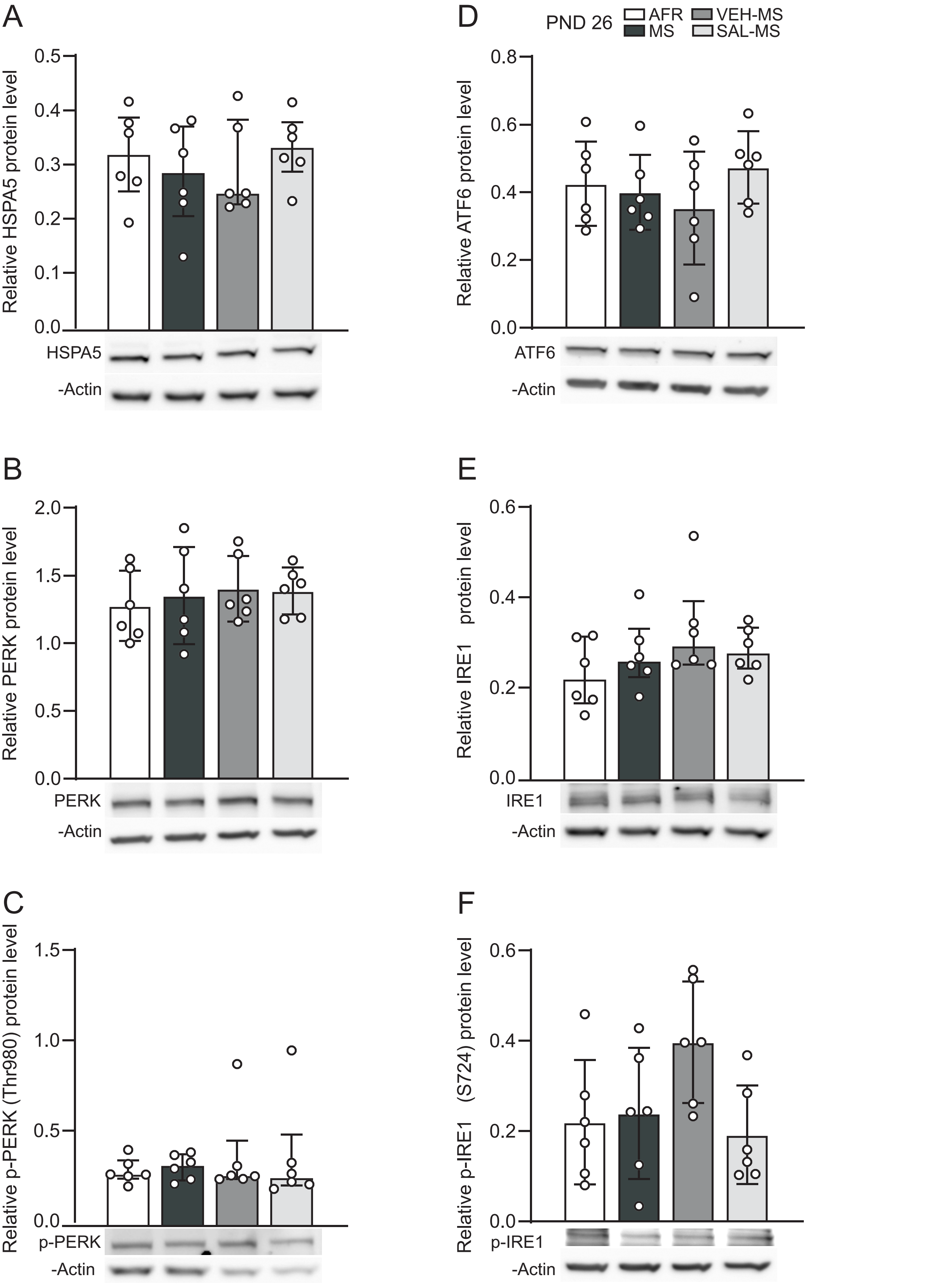

Fig. S4. The effects of MS and early-life SAL/VEH injections on protein expression and activation of ER stress markers in the mPFC of preadolescent rats. (A) HSPA5, (B-C) PERK and p-PERK, (D) ATF6, (E-F) IRE1 and p-IRE1 . The data are presented as the mean ± SD (B, D, F) or median and IQR (A, C, E) and were analyzed by one-way ANOVA or Kruskal-Wallis test, respectively (*n* = 6). Circles represent individual data points. Statistical analysis showed no significant differences between experimental groups. *Abbreviations:* AFR, animal facility rearing; IQR, interquartile range; MS, maternal separation; PND, postnatal day; SAL, salubrinal; VEH, vehicle.

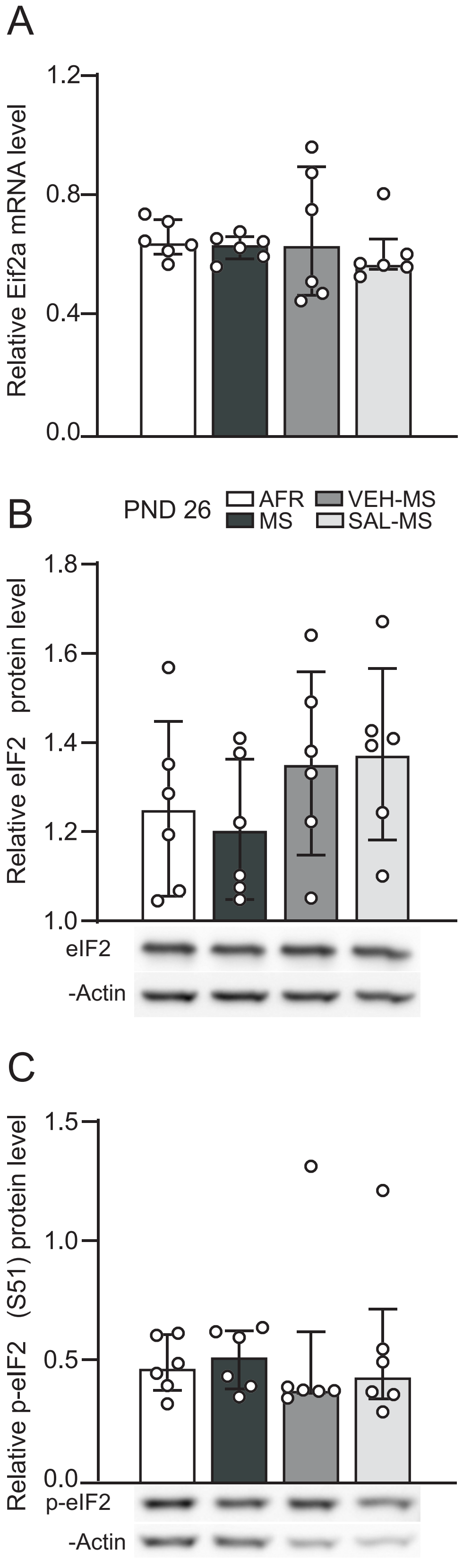

Fig. S5. The effects of MS and early-life SAL/VEH injections on mRNA expression (A) and protein expression and phosphorylation (B-C) of eIF2 in the mPFC of preadolescent rats. The data are presented as the median and IQR (A, C) or mean ± SD (B) and were analyzed by Kruskal-Wallis test or one-way ANOVA, respectively (n = 6). Circles represent individual data points. Statistical analysis showed no significant differences between experimental groups. The same immunoblot of -Actin was used for normalization of both p-eIF2 (C) and p-PERK immunoblots (Fig. S4C). After protein electrotransfer the blots were horizontally cut into two pieces to separately evaluate p-eIF2 and p-PERK protein levels from the same samples. Next, after membrane stripping procedure, appropriate blot was reprobed with -Actin antibody. *Abbreviations:* AFR, animal facility rearing; IQR, interquartile range; MS, maternal separation; PND, postnatal day; SAL, salubrinal; VEH, vehicle.

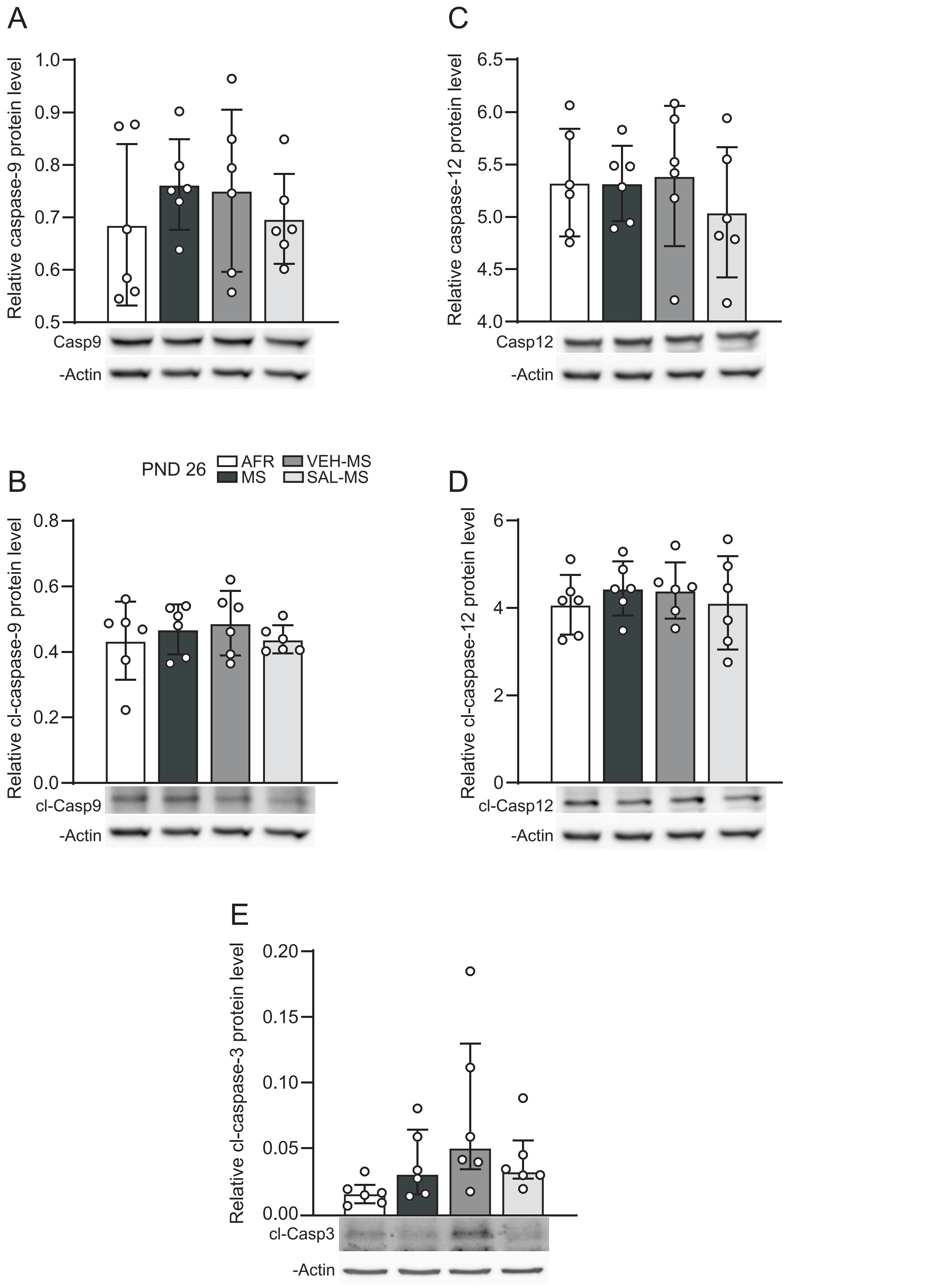

Fig. S6. The effects of MS and early-life SAL/VEH injections on protein expression and cleavage of caspase-9 (A-B), caspase-12 (C-D) and cleavage of caspase-3 (E) in the mPFC of preadolescent rats. The data are presented as the mean ± SD (A-D) or median and IQR (E) and were analyzed by one-way ANOVA or Kruskal-Wallis test, respectively (n = 6). Circles represent individual data points. The same immunoblots of -Actin was used for normalization of specific procaspases and their cleaved forms. Additionally, -Actin blots presented in A, B and C, D served also for normalization of IRE1 (Fig. S4E) and HSPA5 (Fig. S4A) immunoblots, respectively (membrane cutting after protein transfer, stripping and reprobing with -Actin antibody). *Abbreviations:* AFR, animal facility rearing; IQR, interquartile range; MS, maternal separation; PND, postnatal day; SAL, salubrinal; VEH, vehicle.

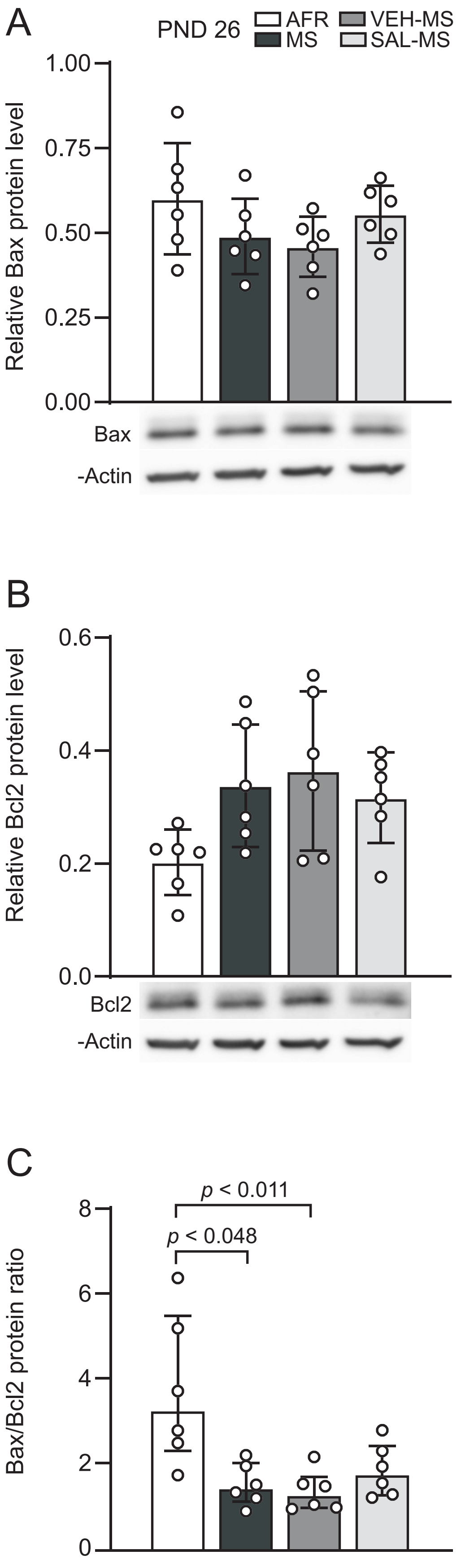

Fig. S7. The effects of MS and early-life SAL/VEH injections on Bax (A) and Bcl2 (B) protein levels and Bax/Bcl2 protein ratio (C) in the mPFC of preadolescent rats. The data are presented as the mean ± SD (A, B) or median and IQR (C) and were analyzed by one-way ANOVA or Kruskal-Wallis test, respectively ($n = 6$). Circles represent individual data points. Connectors indicate statistically significant difference between specific experimental groups in Dunn's test *post hoc* analysis, $p < 0.05$. -Actin blots presented in A and B served also for normalization of PERK (Fig. S4B) and p-IRE1 (Fig. S4F) immunoblots, respectively (membrane cutting after protein transfer and separate evaluation of specific proteins expression from the same samples). *Abbreviations:* AFR, animal facility rearing; IQR, interquartile range; MS, maternal separation; PND, postnatal day; SAL, salubrinal; VEH, vehicle.
