# Supplementary material for: Modulation of the endoplasmic reticulum stress and unfolded protein response mitigates the behavioral effects of early-life stress
Source: Pharmacol Rep. 2023 Feb 27;75(2):293–319. doi: 10.1007/s43440-023-00456-6 (PMC10060333; doi:10.1007/s43440-023-00456-6)
Supplement: Supplementary file 4 — Supplementary file4 (PDF 1219 KB) [file 43440_2023_456_MOESM4_ESM.pdf]

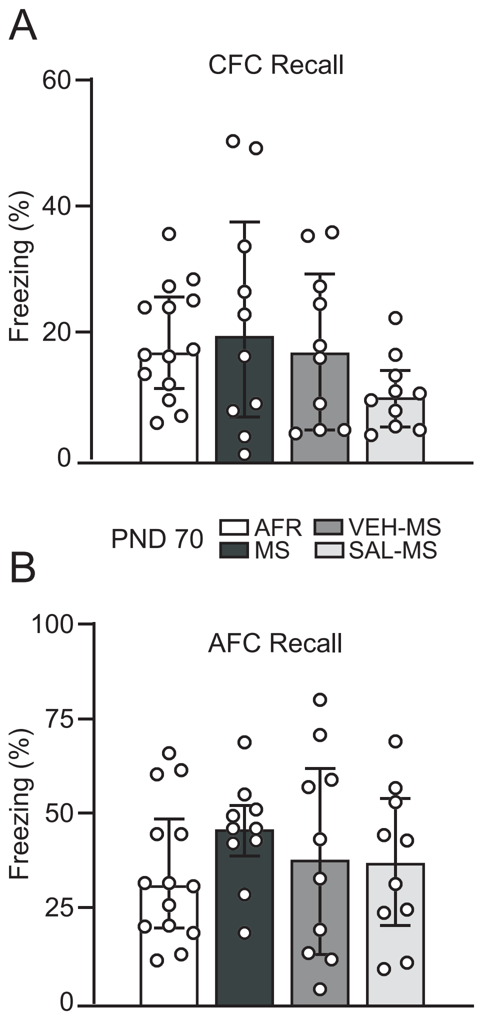

Fig. S8. The effects of MS and early-life SAL/VEH injections on the recall of contextual (A) and auditory (B) fear memories in adulthood. The data are presented as the median and IQR (*n* = 10-14) and expressed as a percentage of the session time. Circles represent individual data points. Kruskal-Wallis test showed no significant differences between experimental groups. *Abbreviations:* AFC, auditory fear conditioning; AFR, animal facility rearing; CFC, contextual fear conditioning; IQR, interquartile range; MS, maternal separation; PND, postnatal day; SAL, salubrinal; VEH, vehicle.
