# Supplementary material for: Modulation of the endoplasmic reticulum stress and unfolded protein response mitigates the behavioral effects of early-life stress
Source: Pharmacol Rep. 2023 Feb 27;75(2):293–319. doi: 10.1007/s43440-023-00456-6 (PMC10060333; doi:10.1007/s43440-023-00456-6)
Supplement: Supplementary file 8 — Supplementary file8 (PDF 13115 KB) [file 43440_2023_456_MOESM8_ESM.pdf]

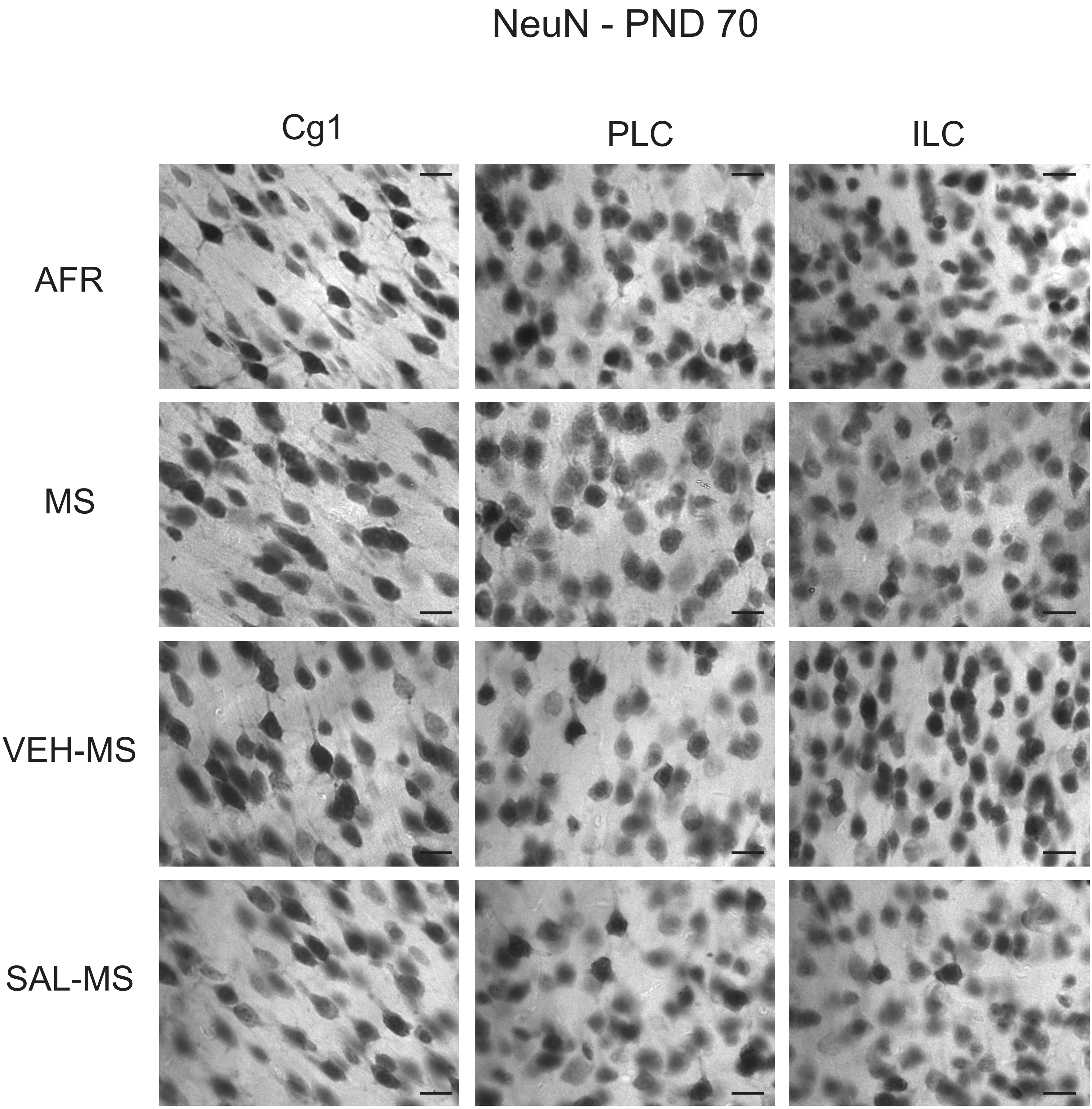

ESM_8. Representative photomicrographs showing NeuN-IR neurons in the subregions of the mPFC (columns) of adult rats. Photomicrographs of the specific experimental groups are presented in rows. *Abbreviations:* AFR, animal facility rearing; Cg1, cingulate cortex 1; ILC, infralimbic cortex; IR, immunoreactive; MS, maternal separation; mPFC, medial prefrontal cortex; PLC, prelimbic cortex; PND, postnatal day; SAL, salubrinal; VEH, vehicle. Scale bar: 20 μm.
