# Supplementary material for: Modulation of the endoplasmic reticulum stress and unfolded protein response mitigates the behavioral effects of early-life stress
Source: Pharmacol Rep. 2023 Feb 27;75(2):293–319. doi: 10.1007/s43440-023-00456-6 (PMC10060333; doi:10.1007/s43440-023-00456-6)
Supplement: Supplementary file 11 — Supplementary file11 (PDF 10308 KB) [file 43440_2023_456_MOESM11_ESM.pdf]

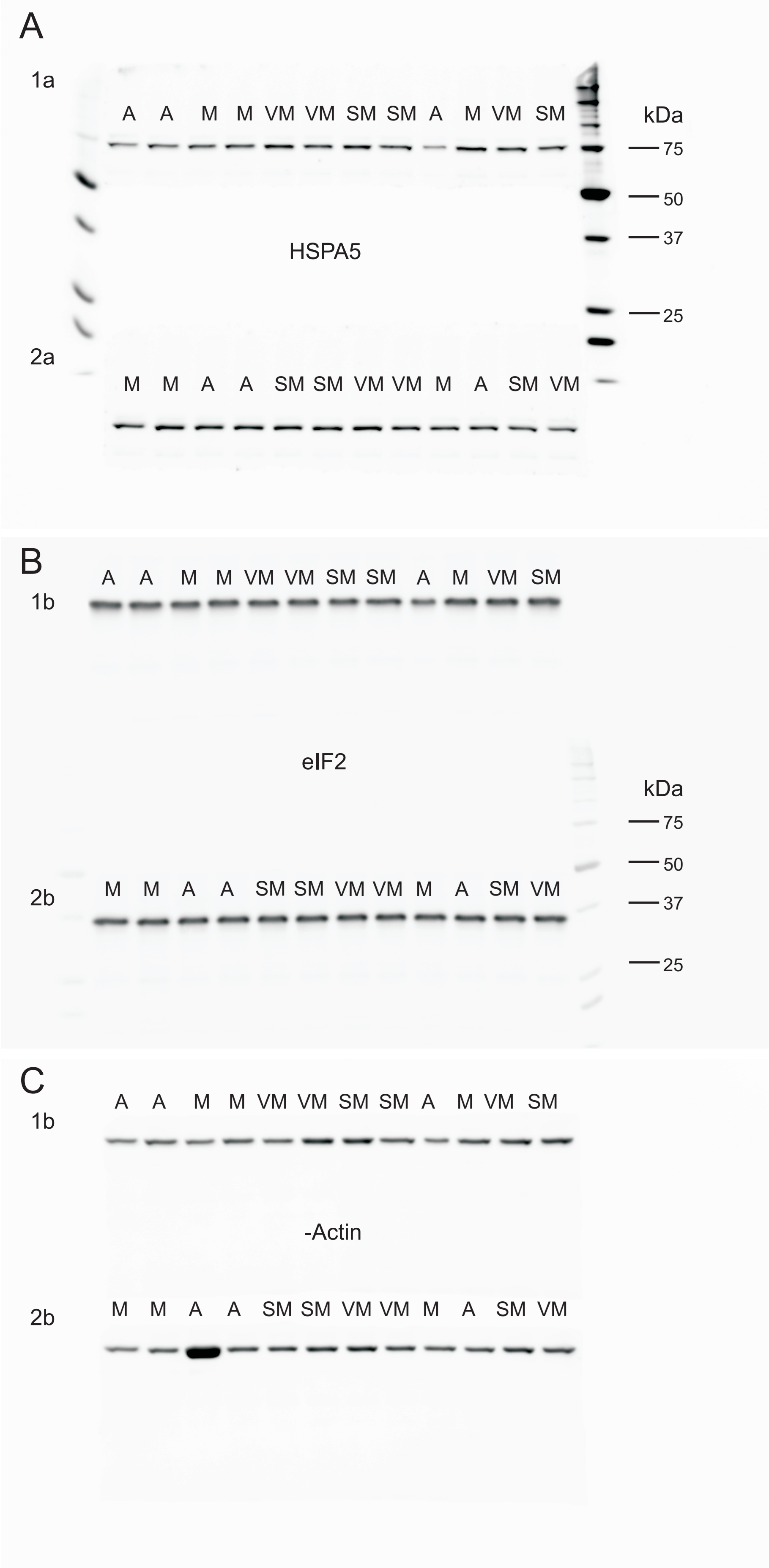

Fig. S9. Original blots presenting HSPA5 (A) and eIF2 (B) expression in juvenile rats and -Actin immunoreactivity as control of gel loading and transfer (C). After a transfer, two distinct blots (1 and 2) were cut into pieces (a and b) slightly above the level of 50 kDa to separately evaluate HSPA5 (1a, 2a) and eIF2 (1b, 2b). Next, after membrane stripping, blots 1b and 2b were reprobed with anti- -Actin antibody. The blots 1a, 2a and 1b, 2b, respectively, were exposed together, therefore they constitute one image. Molecular weight standards were matched only with blot 1a and 2b (B), respectively. *Abbreviations:* A (AFR), animal facility rearing; M (MS), maternal separation; VM (VEH-MS); SM (SAL-MS); SAL, salubrinal; VEH, vehicle.

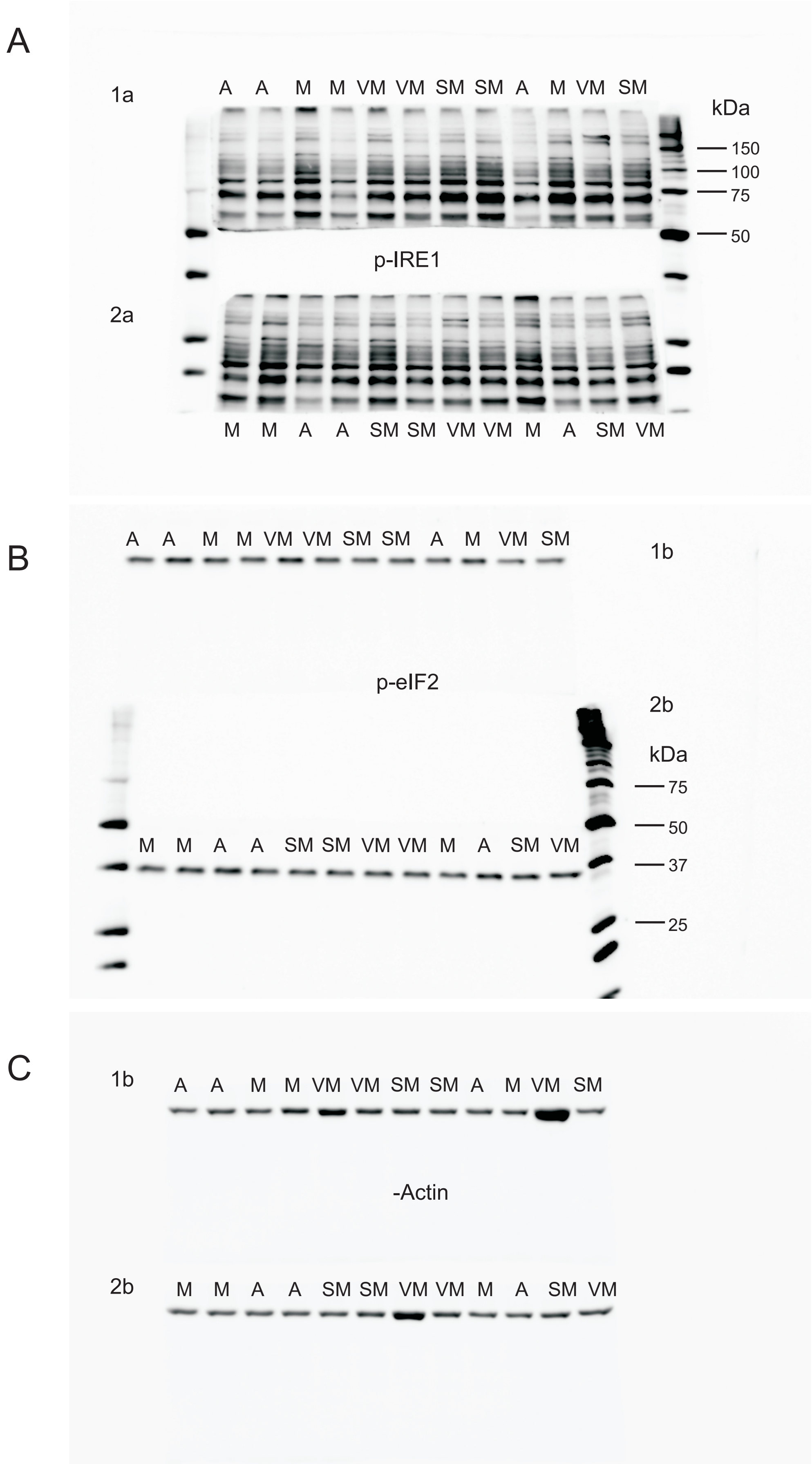

Fig. S10. Original blots presenting p-IRE1 (A) and p-eIF2 (B) expression in juvenile rats and -Actin immunoreactivity as control of gel loading and transfer (C). After a transfer, two distinct blots (1 and 2) were cut into pieces (a and b) slightly above the level of 50 kDa to separately evaluate p-IRE1 (1a, 2a) and p-eIF2 (1b, 2b). Next, after membrane stripping, blots 1b and 2b were reprobed with anti- -Actin antibody. The blots 1a, 2a and 1b, 2b, respectively, were exposed together, therefore they constitute one image. Molecular weight standards were matched only with blot 1a and 2b (B), respectively. Red arrows indicate the band subjected to the analysis. *Abbreviations:* A (AFR), animal facility rearing; M (MS), maternal separation; VM (VEH-MS); SM (SAL-MS); SAL, salubrinal; VEH, vehicle.

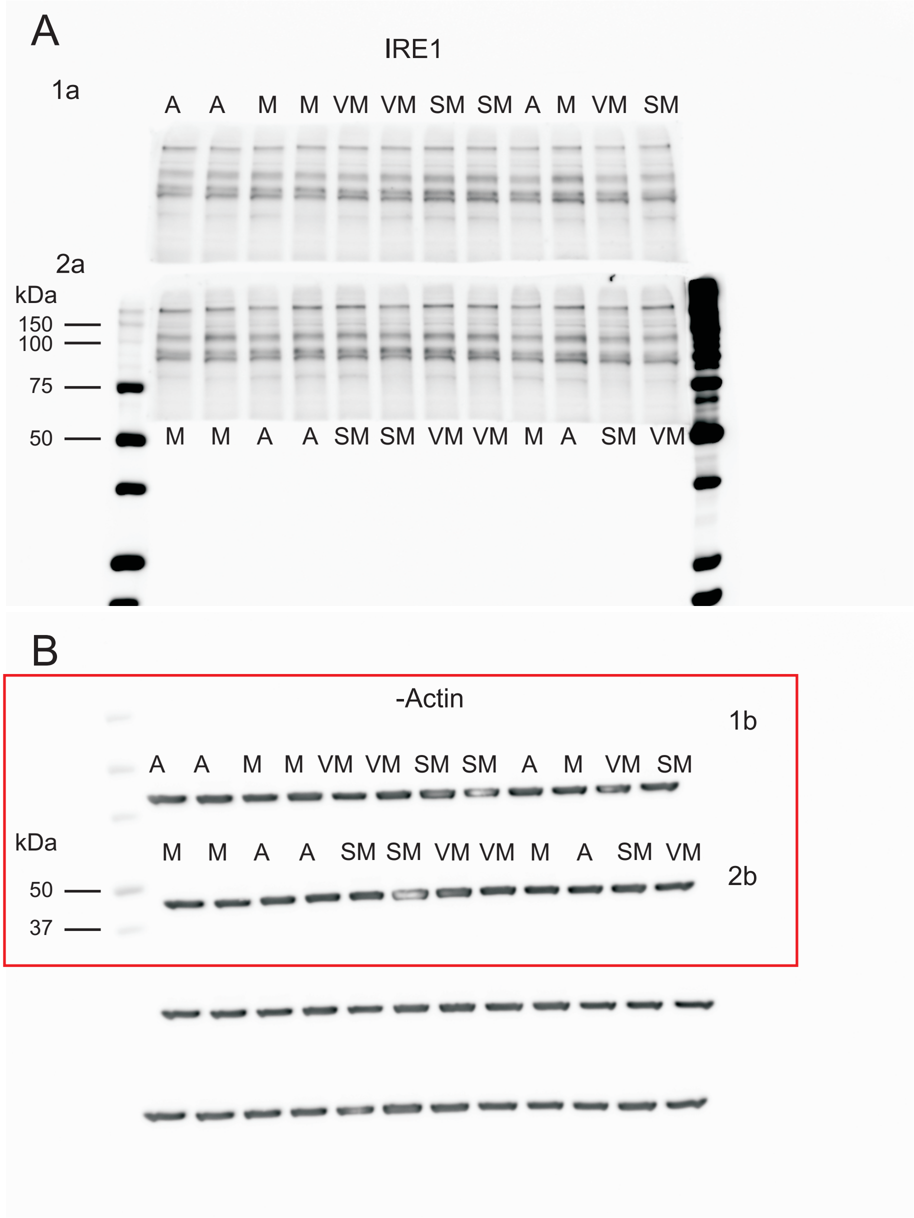

Fig. S11. Original blots presenting IRE1 expression (A) in juvenile rats and -Actin immunoreactivity as control of gel loading and transfer (B). After a transfer, two distinct blots (1 and 2) were cut into pieces (a and b) slightly above the level of 50 kDa to separately evaluate IRE1 (1a, 2a) and -Actin (1b, 2b). The blots 1a, 2a and 1b, 2b, respectively, were exposed together, therefore they constitute one image. Molecular weight standards were matched only with blot 2a and 2b, respectively. Red arrows indicate the band subjected to the analysis. *Abbreviations:* A (AFR), animal facility rearing; M (MS), maternal separation; VM (VEH-MS); SM (SAL-MS); SAL, salubrinal; VEH, vehicle.

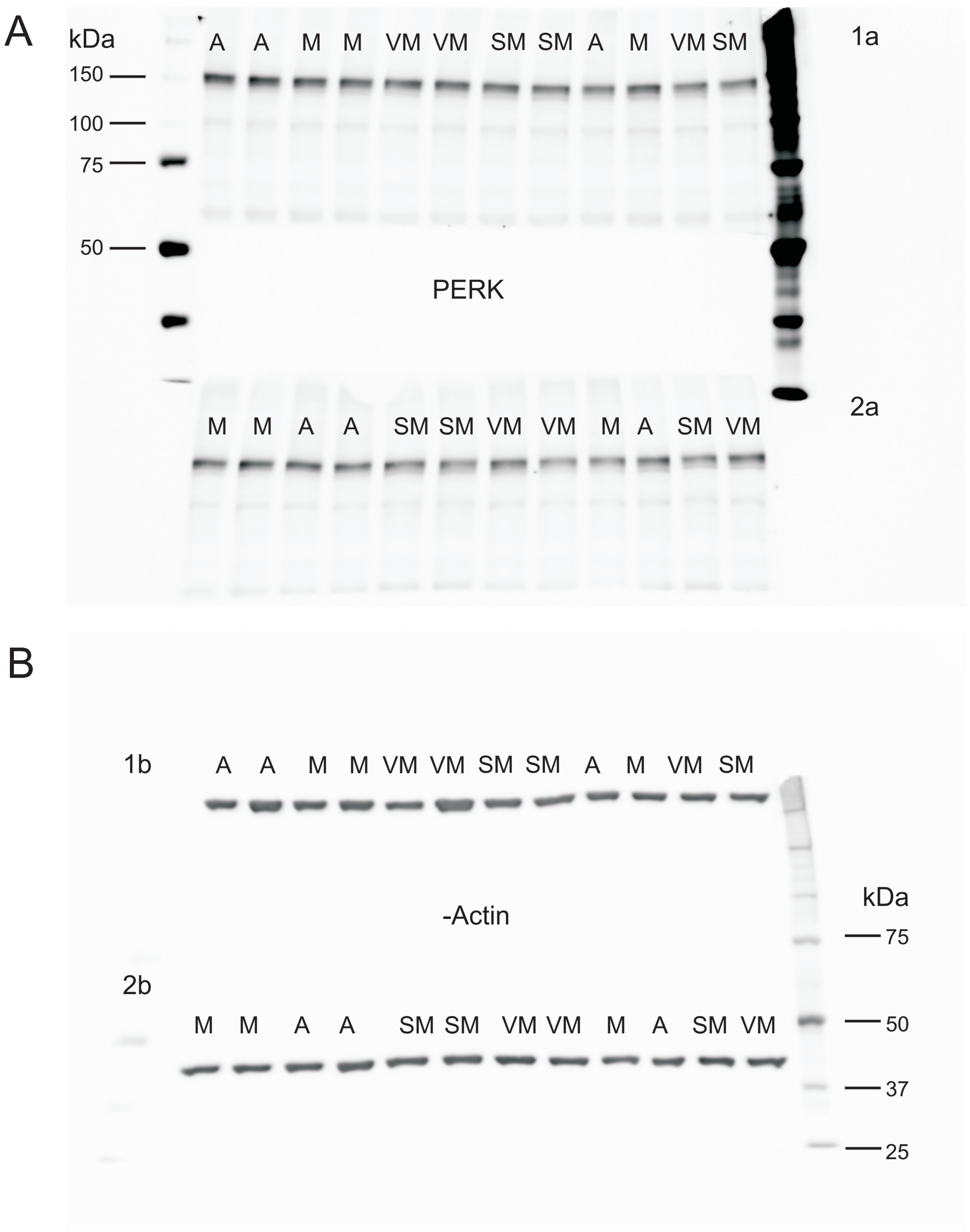

Fig. S12. Original blots presenting PERK expression (A) in juvenile rats and -Actin immunoreactivity as control of gel loading and transfer (B). After a transfer, two distinct blots (1 and 2) were cut into pieces (a and b) slightly above the level of 50 kDa to separately evaluate PERK (1a, 2a) and -Actin (1b, 2b). The blots 1a, 2a and 1b, 2b, respectively, were exposed together, therefore they constitute one image. Molecular weight standards were matched only with blot 1a and 2b, respectively. *Abbreviations:* A (AFR), animal facility rearing; M (MS), maternal separation; VM (VEH-MS); SM (SAL-MS); SAL, salubrinal; VEH, vehicle.

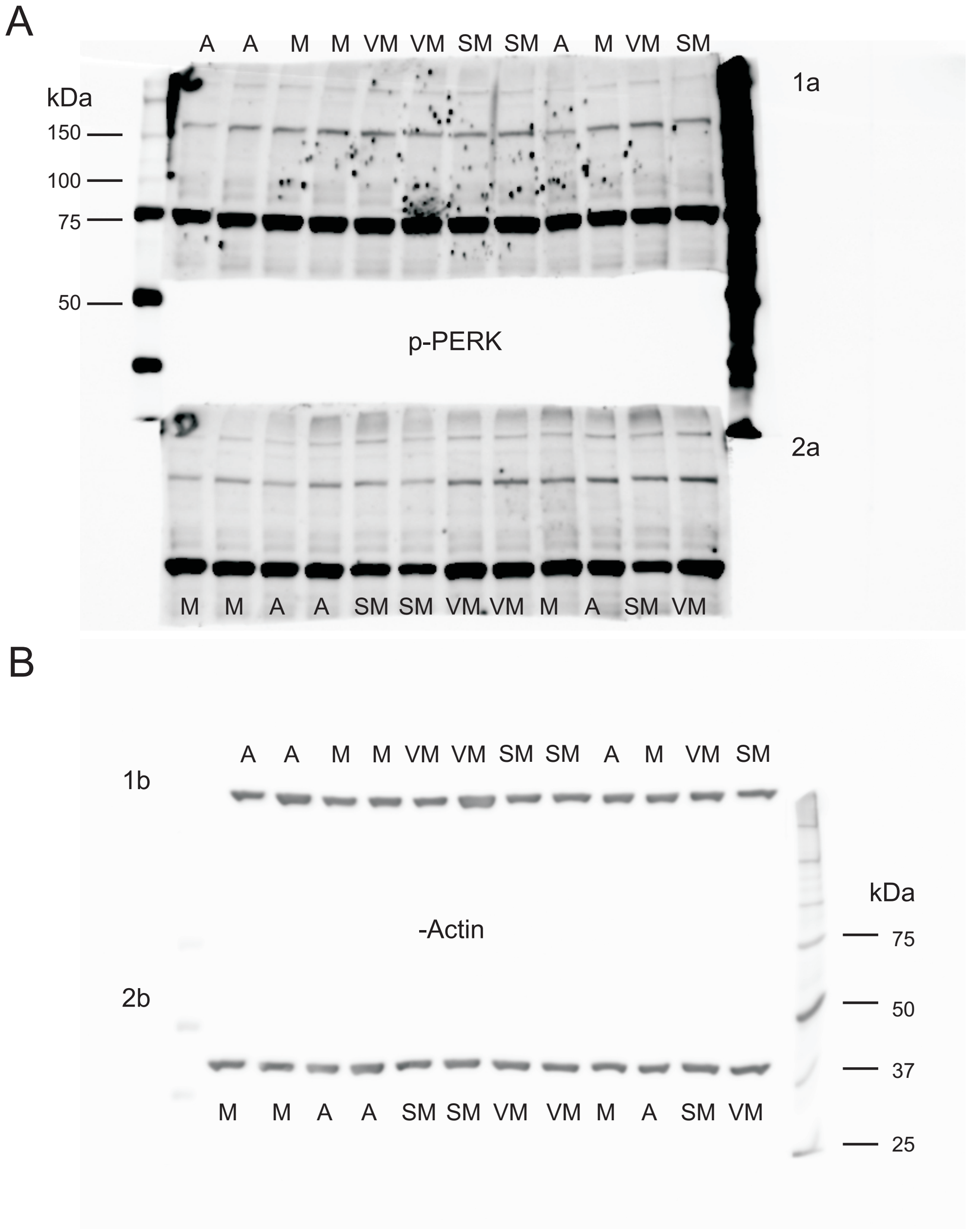

Fig. S13. Original blots presenting p-PERK expression (A) in juvenile rats and -Actin immunoreactivity as control of gel loading and transfer (B). After a transfer, two distinct blots (1 and 2) were cut into pieces (a and b) slightly above the level of 50 kDa to separately evaluate p-PERK (1a, 2a) and -Actin (1b, 2b). The blots 1a, 2a and 1b, 2b, respectively, were exposed together, therefore they constitute one image. Molecular weight standards were matched only with blot 1a and 2b, respectively. Red arrows indicate the band subjected to the analysis. *Abbreviations:* A (AFR), animal facility rearing; M (MS), maternal separation; VM (VEH-MS); SM (SAL-MS); SAL, salubrinal; VEH, vehicle.

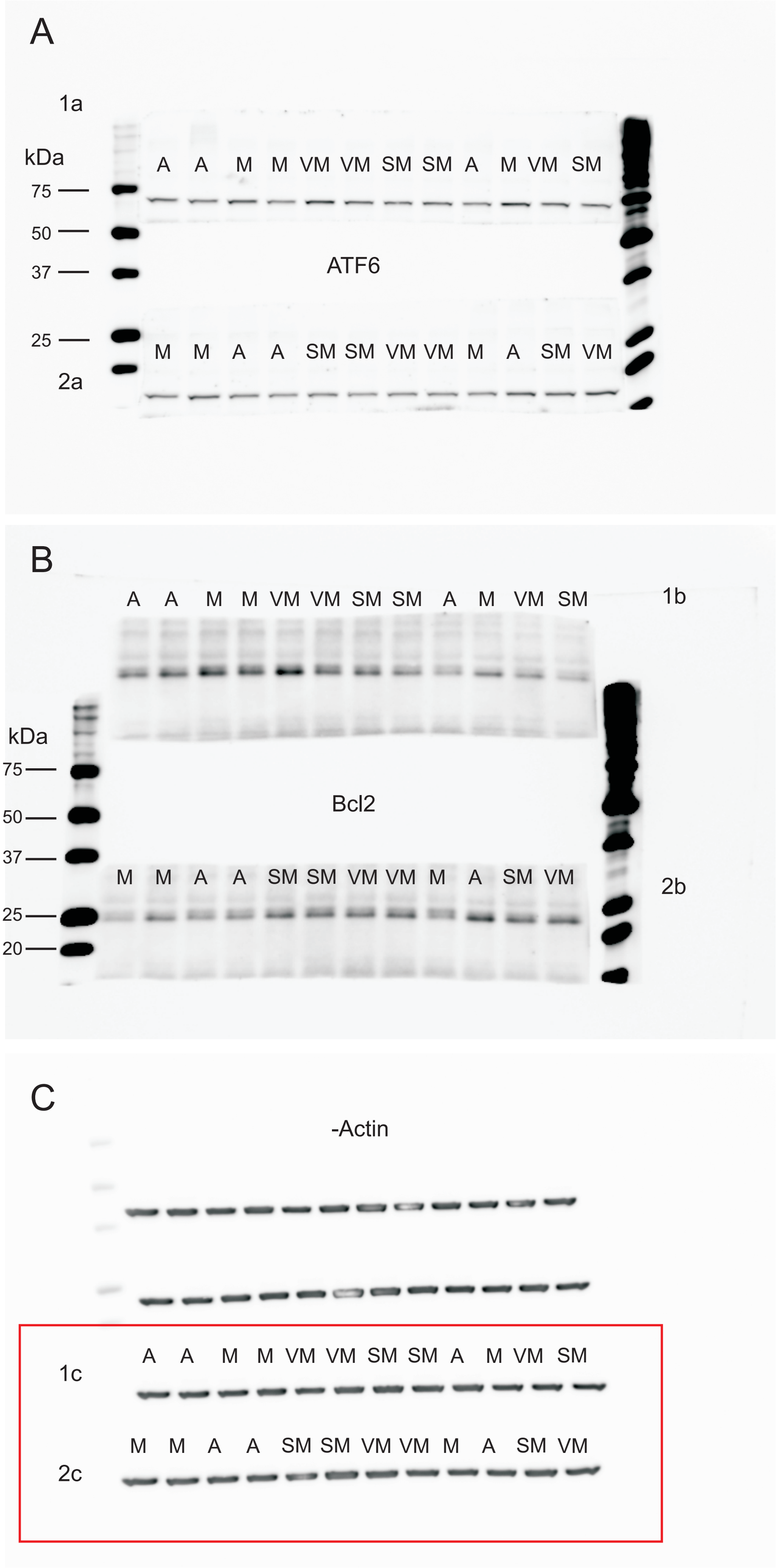

Fig. S14. Original blots presenting ATF6 (A) and Bcl2 (B) expression in juvenile rats and -Actin immunoreactivity as control of gel loading and transfer (C). After a transfer, two distinct blots (1 and 2) were cut into pieces (a, b and c) slightly above the level of 50 kDa and below 37 kDa to separately evaluate ATF6 (1a, 2a), Bcl2 (1b, 2b) and -Actin (1c, 2c). The blots 1a, 2a and 1b, 2b and 1c, 2c, respectively, were exposed together, therefore they constitute one image. Molecular weight standards were matched only with blot 1a and 2b. *Abbreviations:* A (AFR), animal facility rearing; M (MS), maternal separation; VM (VEH-MS); SM (SAL-MS); SAL, salubrinal; VEH, vehicle.

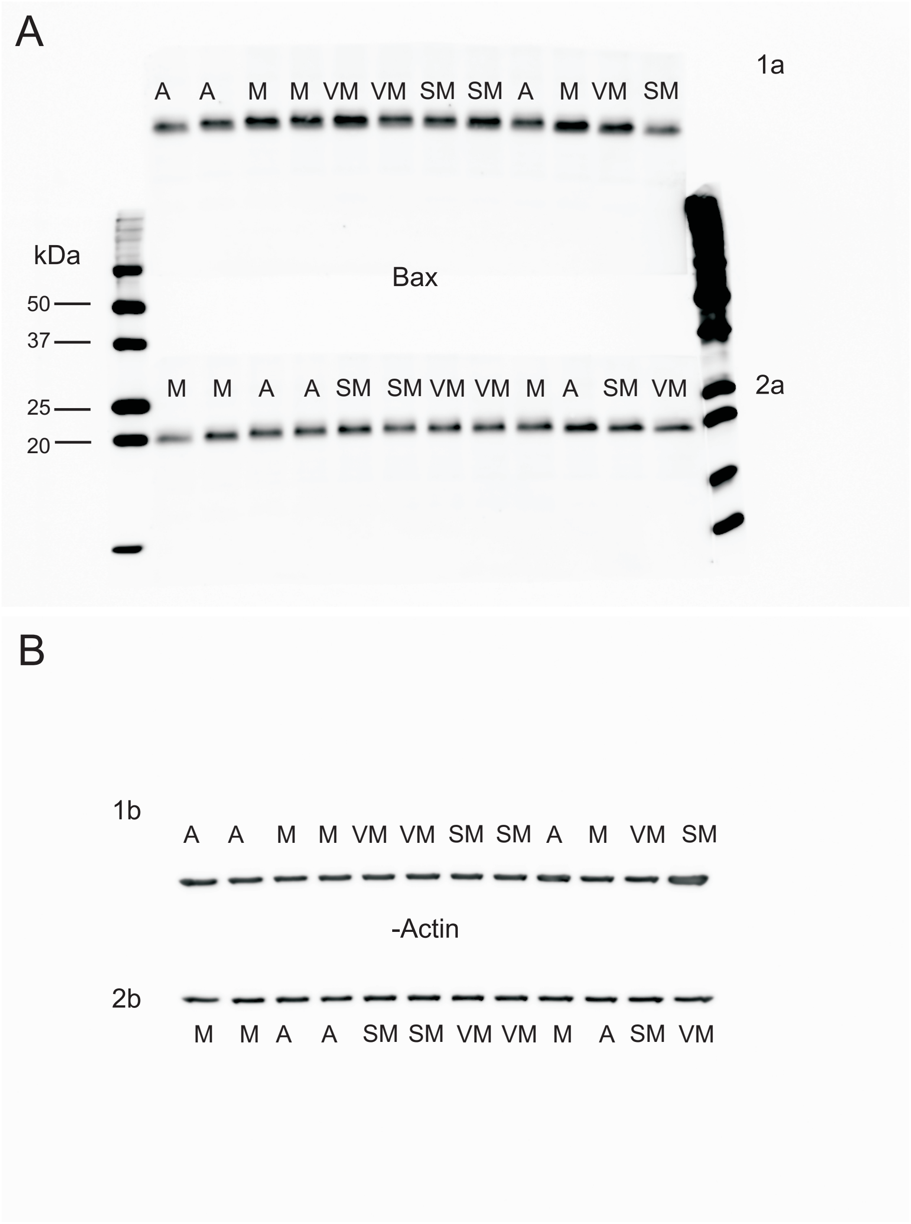

Fig. S15. Original blots presenting Bax expression (A) in juvenile rats and -Actin immunoreactivity as control of gel loading and transfer (B). After a transfer, two distinct blots (1 and 2) were cut into pieces (a and b) slightly below the level of 37 kDa to separately evaluate Bax (1a, 2a) and -Actin (1b, 2b). The blots 1a, 2a and 1b, 2b, respectively, were exposed together, therefore they constitute one image. Molecular weight standards were matched only with blot 2a. *Abbreviations:* A (AFR), animal facility rearing; M (MS), maternal separation; VM (VEH-MS); SM (SAL-MS); SAL, salubrinal; VEH, vehicle.
