# Supplementary material for: Modulation of the endoplasmic reticulum stress and unfolded protein response mitigates the behavioral effects of early-life stress
Source: Pharmacol Rep. 2023 Feb 27;75(2):293–319. doi: 10.1007/s43440-023-00456-6 (PMC10060333; doi:10.1007/s43440-023-00456-6)
Supplement: Supplementary file 12 — Supplementary file12 (PDF 2971 KB) [file 43440_2023_456_MOESM12_ESM.pdf]

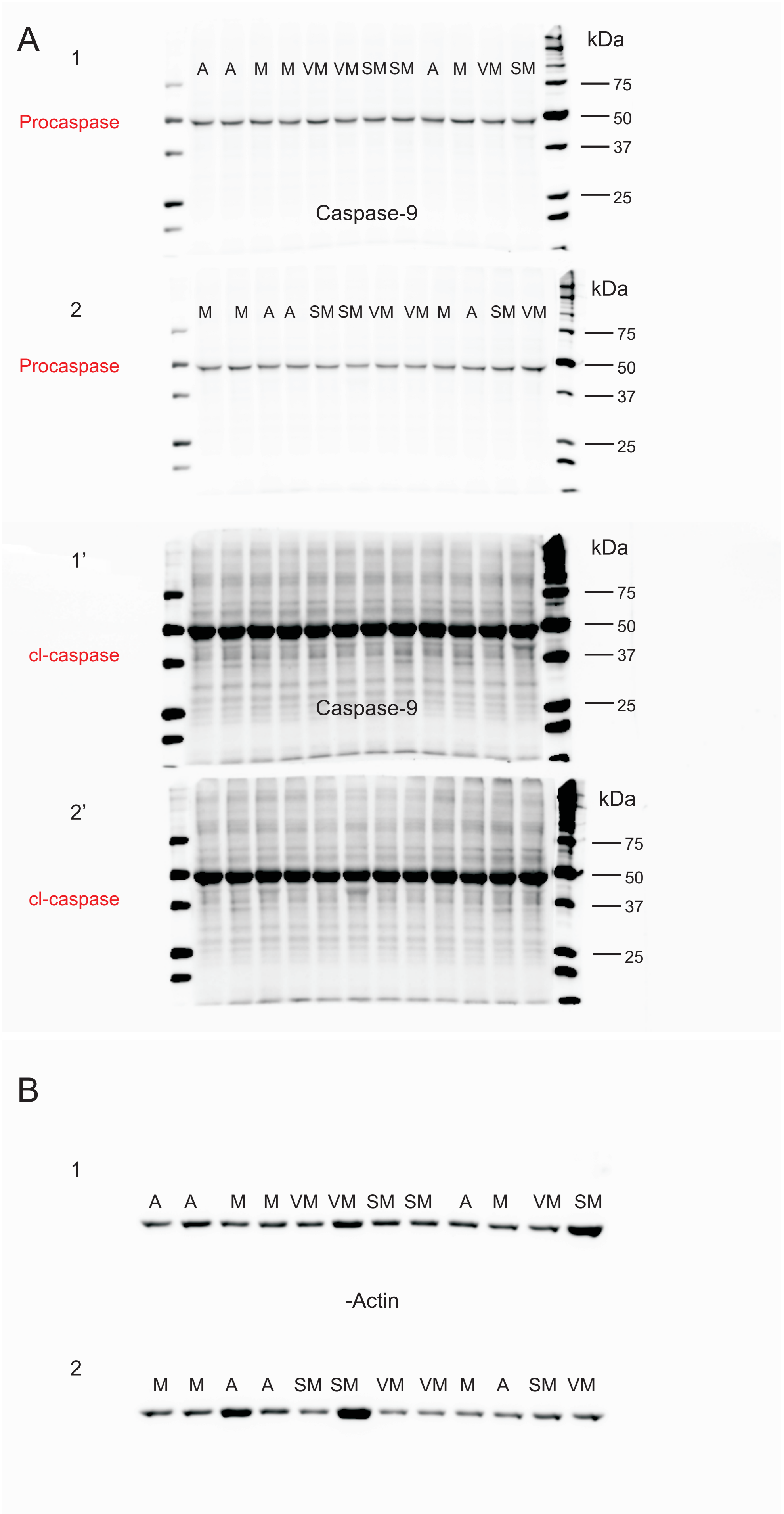

Fig. S16. Original blots presenting caspase-9 expression in juvenile rats (A). After membrane stripping, blots 1 and 2 were reprobed with anti- -Actin antibody to control gel loading and transfer (B). The blots 1, 2 were exposed together, therefore they constitute one image. Lower image in A (1’, 2’) was subjected to a higher exposure time than upper image (the same blots) to evaluate cl-caspase levels. Red arrows indicate the bands subjected to the analysis. *Abbreviations:* A (AFR), animal facility rearing; cl, cleaved; M (MS), maternal separation; VM (VEH-MS); SM (SAL-MS); SAL, salubrinal; VEH, vehicle.

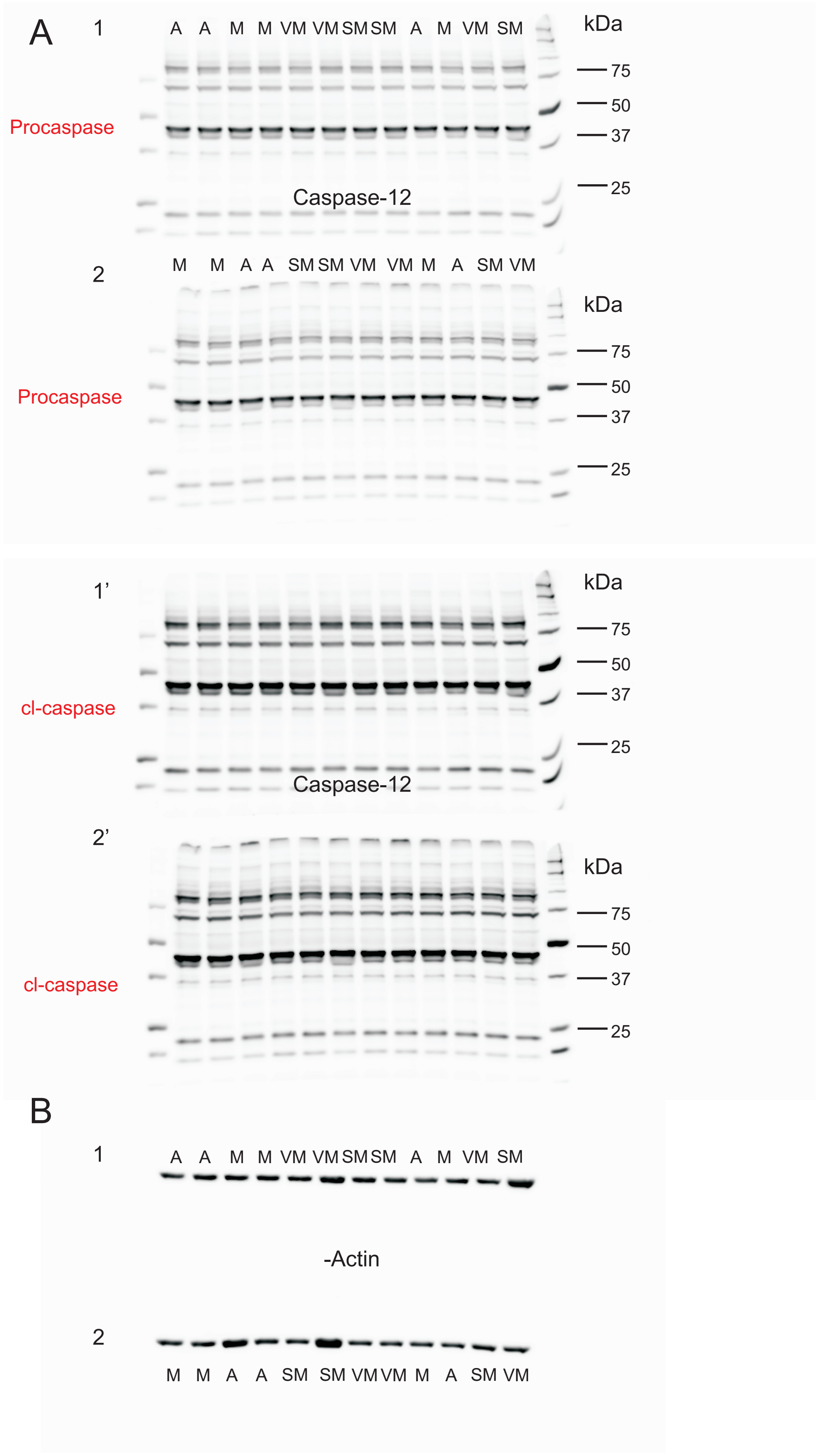

Fig. S17. Original blots presenting caspase-12 expression in juvenile rats (A). After membrane stripping, blots 1 and 2 were reprobed with anti- -Actin antibody to control gel loading and transfer (B). The blots 1, 2 were exposed together, therefore they constitute one image. Lower image in A (1’, 2’) was subjected to a higher exposure time than upper image (the same blots) to evaluate cl-caspase levels. Red arrows indicate the bands subjected to the analysis. *Abbreviations:* A (AFR), animal facility rearing; cl, cleaved; M (MS), maternal separation; VM (VEH-MS); SM (SAL-MS); SAL, salubrinal; VEH, vehicle.

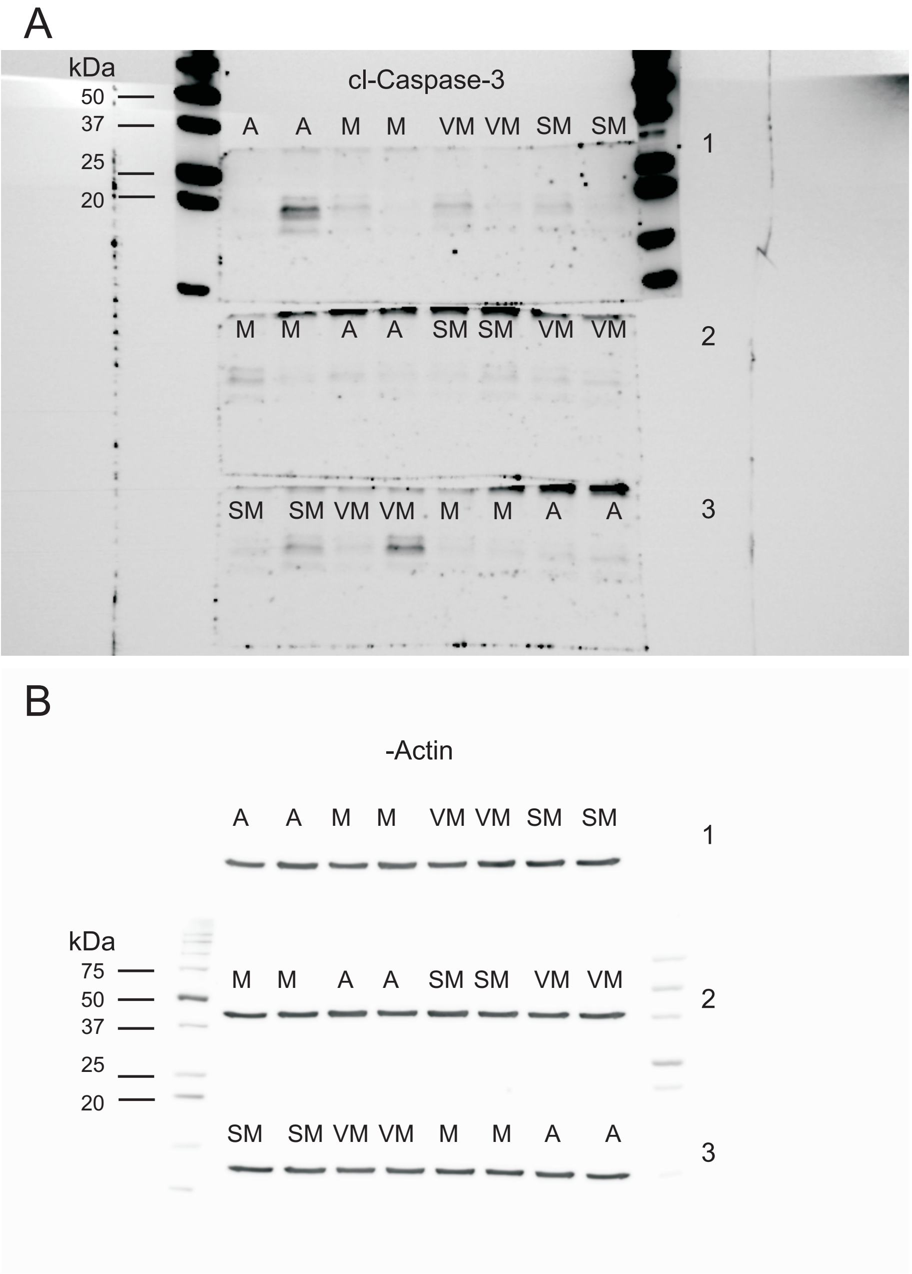

Fig. S18. Original blots presenting cl-caspase-3 expression in juvenile rats (A). After membrane stripping, blots 1, 2 and 3 were reprobed with anti- -Actin antibody to control gel loading and transfer (B). The blots 1, 2, 3 were exposed together, therefore they constitute one image. Red arrows indicate the band subjected to the analysis. Molecular weight standards were matched only with blot 1 (A) and 2 (B). *Abbreviations:* A (AFR), animal facility rearing; cl, cleaved; M (MS), maternal separation; VM (VEH-MS); SM (SAL-MS); SAL, salubrinal; VEH, vehicle,
