# Supplementary material for: Modulation of the endoplasmic reticulum stress and unfolded protein response mitigates the behavioral effects of early-life stress
Source: Pharmacol Rep. 2023 Feb 27;75(2):293–319. doi: 10.1007/s43440-023-00456-6 (PMC10060333; doi:10.1007/s43440-023-00456-6)
Supplement: Supplementary file 13 — Supplementary file13 (PDF 6188 KB) [file 43440_2023_456_MOESM13_ESM.pdf]

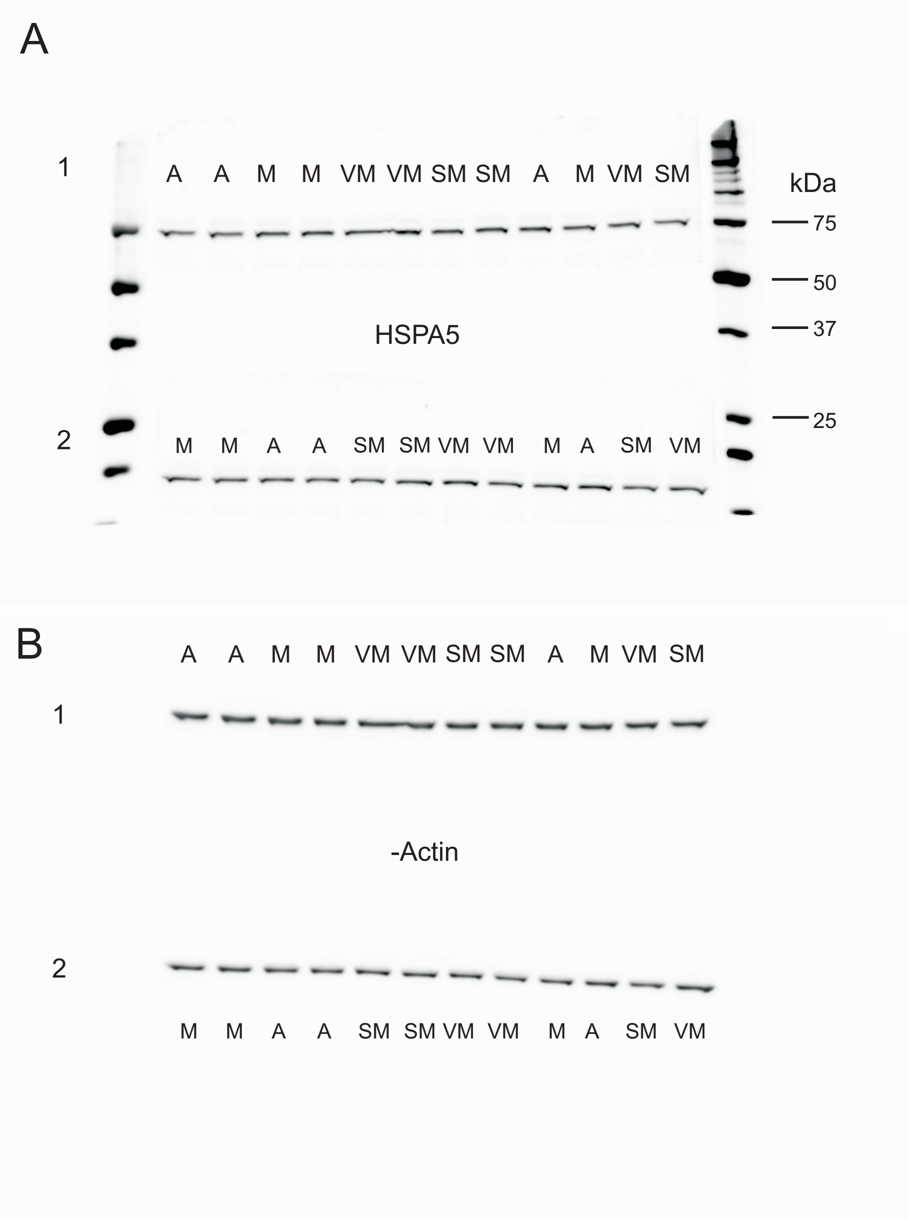

Fig. S19. Original blots presenting HSPA5 expression (A) in preadolescent rats and -Actin immunoreactivity as control of gel loading and transfer (B). After a transfer, two distinct blots (1 and 2) were cut into pieces above the level of 50 kDa to separately evaluate HSPA5, caspase-12 and finally -Actin (after membrane stripping), therefore -Actin blot served also for normalization of caspas-12 blot (Fig. S27). The blots 1 and 2, respectively, were exposed together, therefore they constitute one image. Molecular weight standards were matched only with blot 1 (A). *Abbreviations:* A (AFR), animal facility rearing; M (MS), maternal separation; VM (VEH-MS); SM (SAL-MS); SAL, salubrinal; VEH, vehicle.

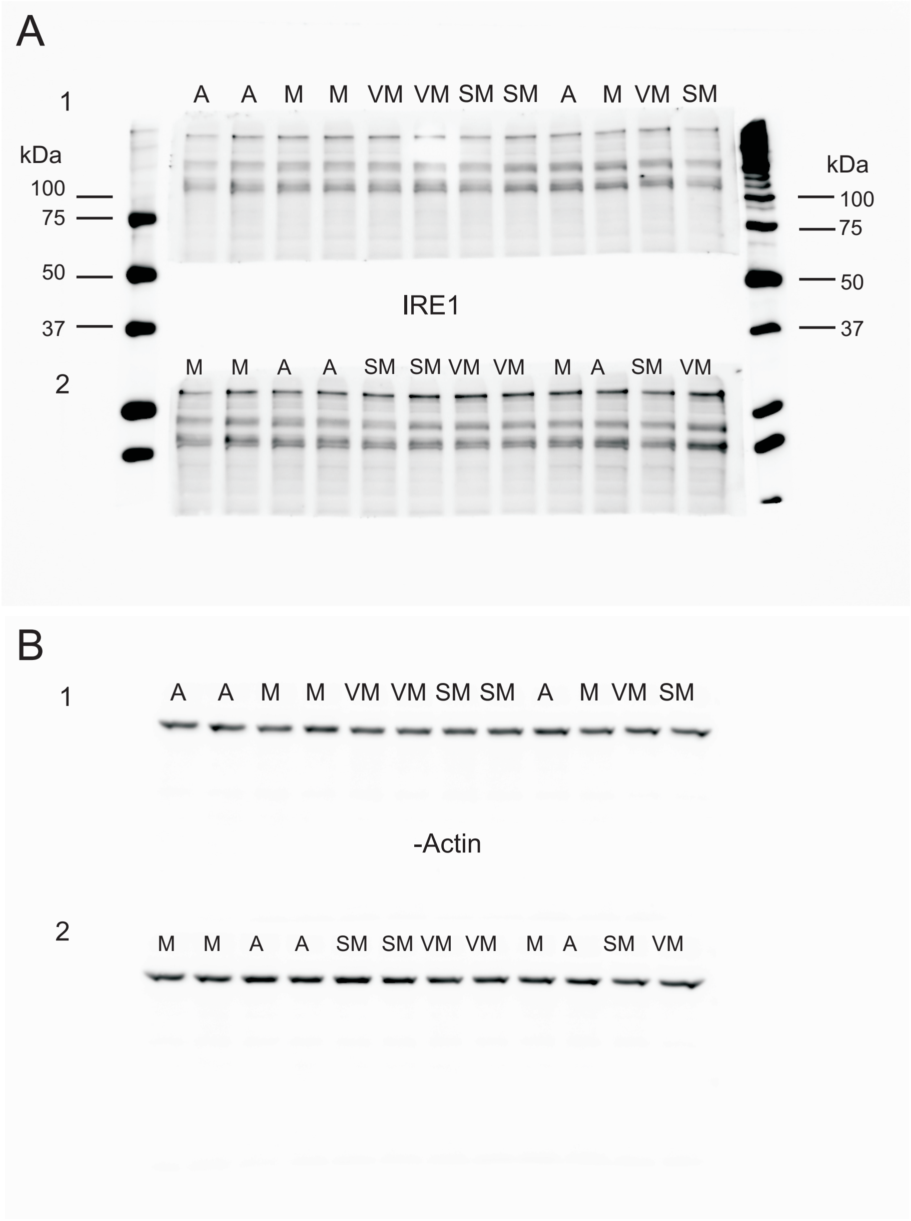

Fig. S20. Original blots presenting IRE1 expression (A) in preadolescent rats and -Actin immunoreactivity as control of gel loading and transfer (B). After a transfer, two distinct blots (1 and 2) were cut into pieces above the level of 50 kDa to separately evaluate IRE1 , caspase-9 and finally -Actin (after membrane stripping), therefore -Actin blot served also for normalization of caspas-9 blot (Fig. S26). The blots 1 and 2, respectively, were exposed together, therefore they constitute one image. Molecular weight standards were matched only with blot 1 (A). Red arrows indicate the band subjected to the analysis. *Abbreviations:* A (AFR), animal facility rearing; M (MS), maternal separation; VM (VEH-MS); SM (SAL-MS); SAL, salubrinal; VEH, vehicle.

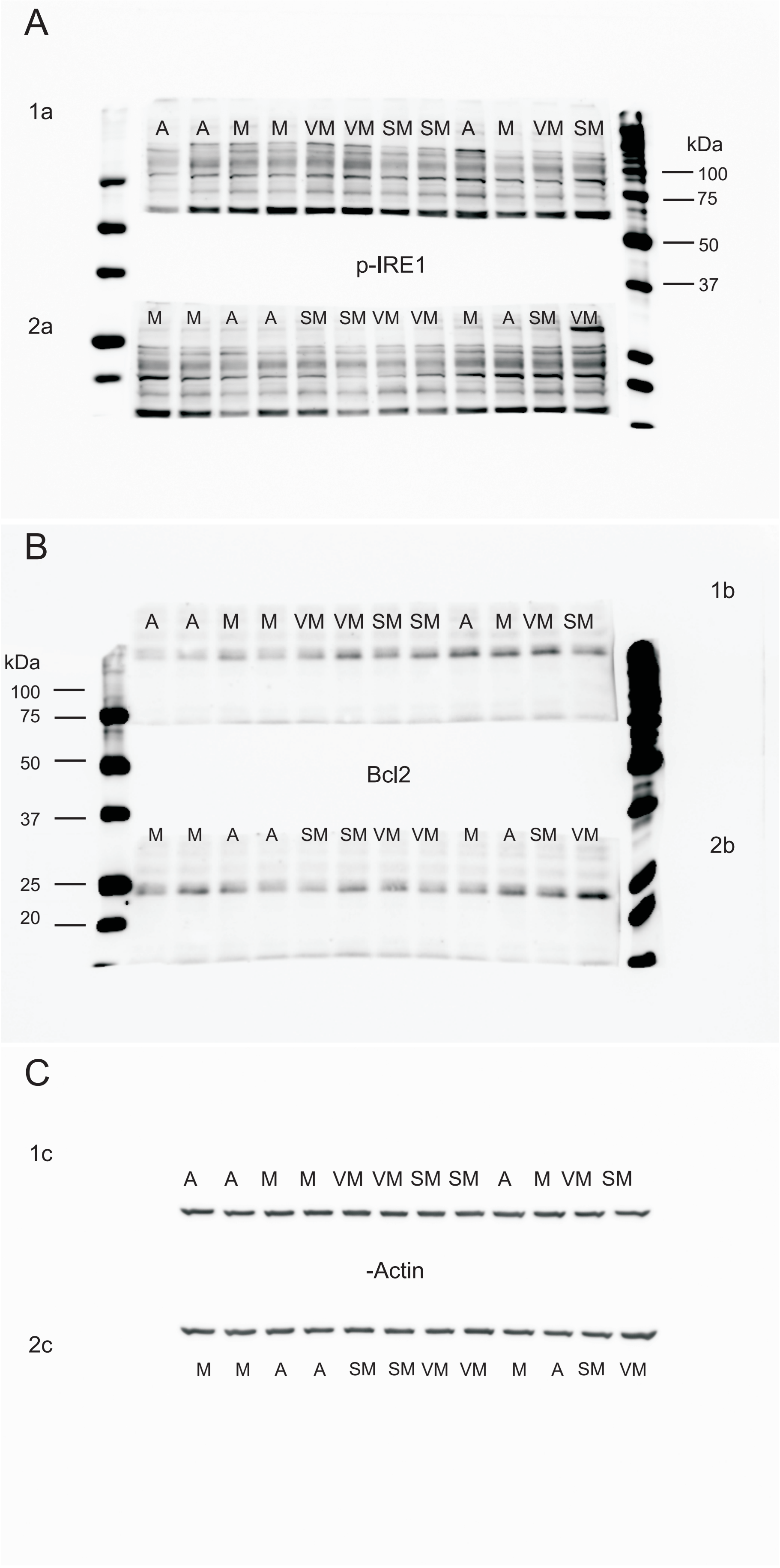

Fig. S21. Original blots presenting p-IRE1 (A) and Bcl2 (B) expression in preadolescent rats and -Actin immunoreactivity as control of gel loading and transfer (C). After a transfer, two distinct blots (1 and 2) were cut into pieces (a, b and c) slightly above the level of 50 kDa and below 37 kDa to separately evaluate p-IRE1 (1a, 2a), Bcl2 (1b, 2b) and -Actin (1c, 2c). The blots 1a, 2a and 1b, 2b and 1c, 2c, respectively, were exposed together, therefore they constitute one image. Molecular weight standards were matched only with blot 1a and 2b. Red arrows indicate the band subjected to the analysis. *Abbreviations:* A (AFR), animal facility rearing; M (MS), maternal separation; VM (VEH-MS); SM (SAL-MS); SAL, salubrinal; VEH, vehicle.

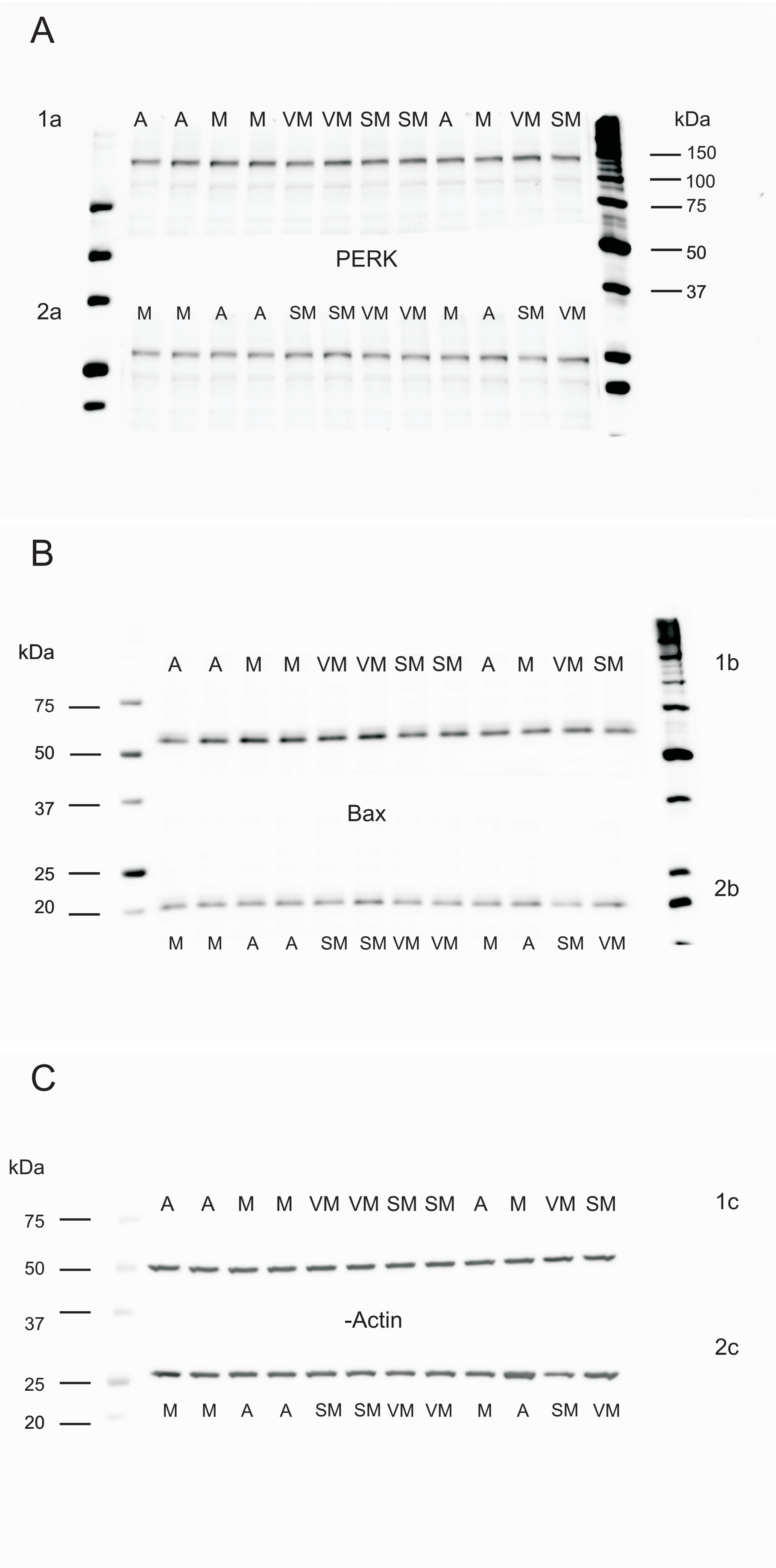

Fig. S22. Original blots presenting PERK (A) and Bax (B) expression in preadolescent rats and -Actin immunoreactivity as control of gel loading and transfer (C). After a transfer, two distinct blots (1 and 2) were cut into pieces (a, b and c) slightly above the level of 50 kDa and below 37 kDa to separately evaluate PERK (1a, 2a), Bax (1b, 2b) and -Actin (1c, 2c). The blots 1a, 2a and 1b, 2b and 1c, 2c, respectively, were exposed together, therefore they constitute one image. Molecular weight standards were matched only with blot 1a, 2b and 1c . *Abbreviations:* A (AFR), animal facility rearing; M (MS), maternal separation; VM (VEH-MS); SM (SAL-MS); SAL, salubrinal; VEH, vehicle.

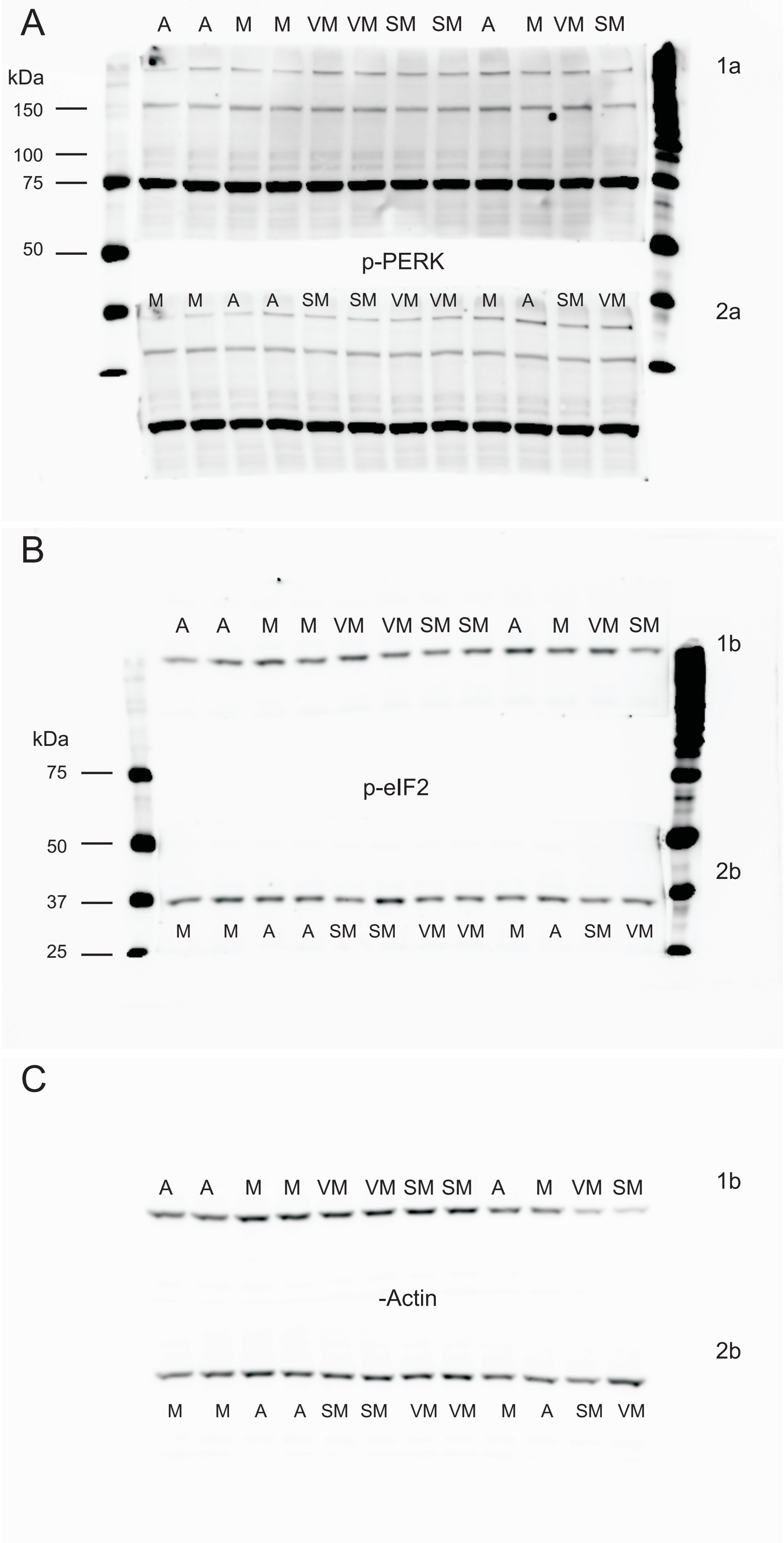

Fig. S23. Original blots presenting p-PERK (A) and p-eIF2 (B) expression in preadolescent rats and -Actin immunoreactivity as control of gel loading and transfer (C). After a transfer, two distinct blots (1 and 2) were cut into pieces (a and b) slightly above the level of 50 kDa to separately evaluate p-PERK (1a, 2a) and p-eIF2 (1b, 2b). Next, after membrane stripping, blots 1b and 2b were reprobed with anti- -Actin antibody. The blots 1a, 2a and 1b, 2b, respectively, were exposed together, therefore they constitute one image. Molecular weight standards were matched only with blot 1a and 2b (B), respectively. Red arrows indicate the band subjected to the analysis. *Abbreviations:* A (AFR), animal facility rearing; M (MS), maternal separation; VM (VEH-MS); SM (SAL-MS); SAL, salubrinal; VEH, vehicle.

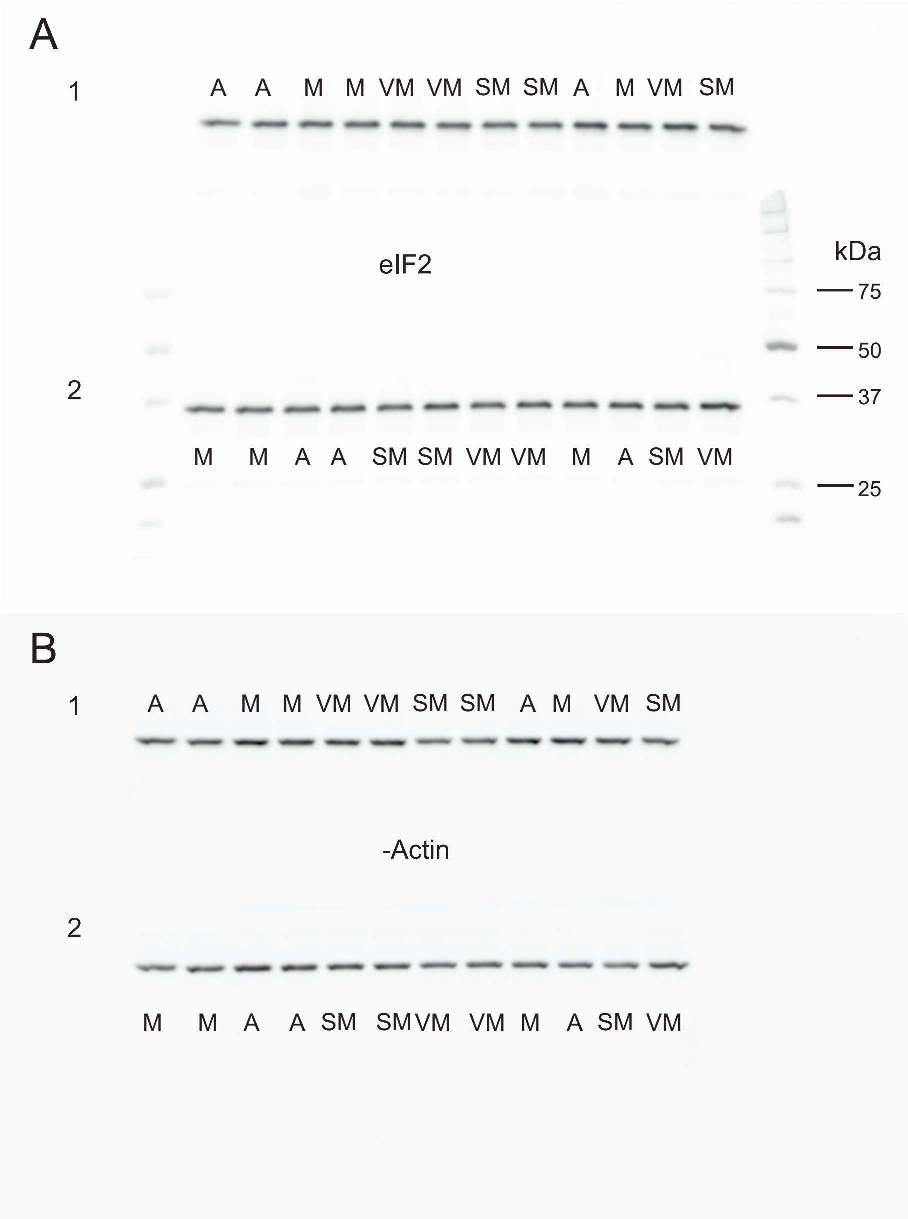

Fig. S24. Original blots presenting eIF2 expression in preadolescent rats (A). After membrane stripping, blots 1 and 2 were reprobed with anti- -Actin antibody to control gel loading and transfer (B). The blots 1 and 2 were exposed together, therefore they constitute one image. Molecular weight standards were matched only with blot 2 (A). *Abbreviations:* A (AFR), animal facility rearing; M (MS), maternal separation; VM (VEH-MS); SM (SAL-MS); SAL, salubrinal; VEH, vehicle.

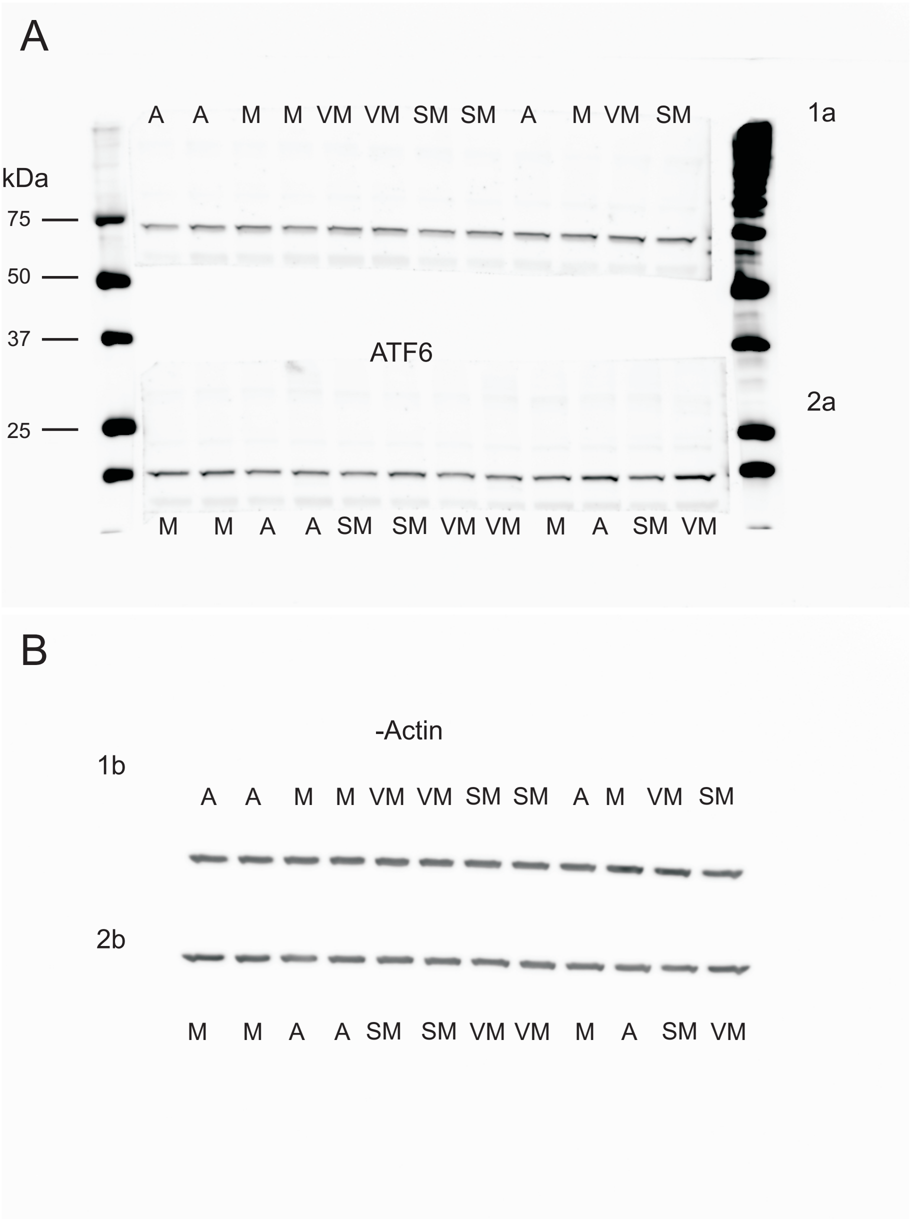

Fig. S25. Original blots presenting ATF6 expression in preadolescent rats (A) and -Actin immunoreactivity as control of gel loading and transfer (B). After a transfer, two distinct blots (1 and 2) were cut into pieces (a and b) slightly above the level of 50 kDa to separately evaluate ATF6 (1a, 2a) and -Actin (1b, 2b). The blots 1a, 2a and 1b, 2b, respectively, were exposed together, therefore they constitute one image. Molecular weight standards were matched only with blot 1a. *Abbreviations:* A (AFR), animal facility rearing; M (MS), maternal separation; VM (VEH-MS); SM (SAL-MS); SAL, salubrinal; VEH, vehicle.
