# Supplementary material for: Modulation of the endoplasmic reticulum stress and unfolded protein response mitigates the behavioral effects of early-life stress
Source: Pharmacol Rep. 2023 Feb 27;75(2):293–319. doi: 10.1007/s43440-023-00456-6 (PMC10060333; doi:10.1007/s43440-023-00456-6)
Supplement: Supplementary file 14 — Supplementary file14 (PDF 3250 KB) [file 43440_2023_456_MOESM14_ESM.pdf]

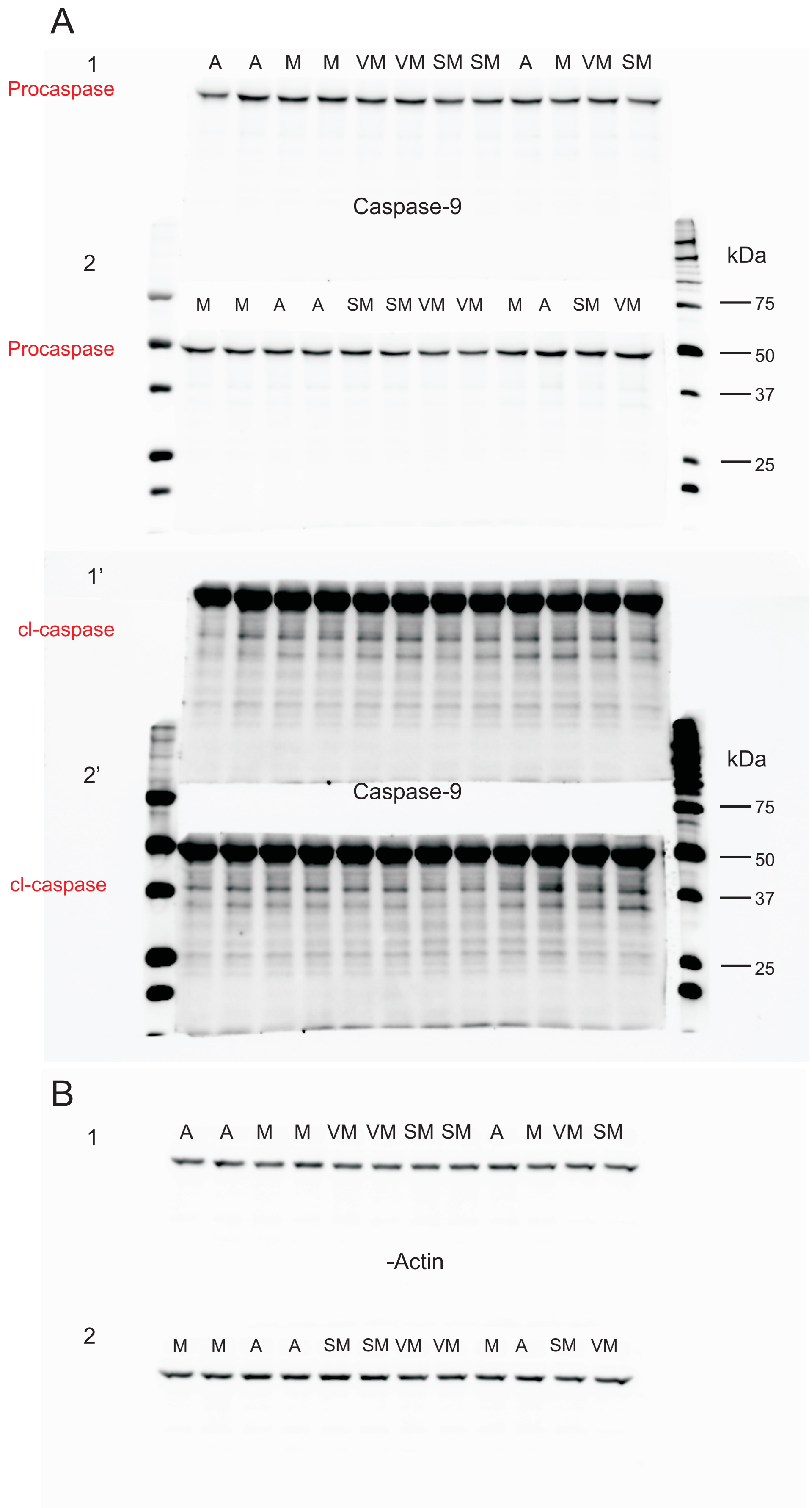

Fig. S26. Original blots presenting caspase-9 expression in preadolescent rats (A). After membrane stripping, blots 1 and 2 were reprobed with anti- -Actin antibody to control gel loading and transfer (B). The blots 1, 2 were exposed together, therefore they constitute one image. Lower image in A (1’, 2’) was subjected to a higher exposure time than upper image (the same blots) to evaluate cl-caspase levels. Molecular weight standards were matched only with blot 2. Red arrows indicate the bands subjected to the analysis. *Abbreviations:* A (AFR), animal facility rearing; cl, cleaved; M (MS), maternal separation; VM (VEH-MS); SM (SAL-MS); SAL, salubrinal; VEH, vehicle.

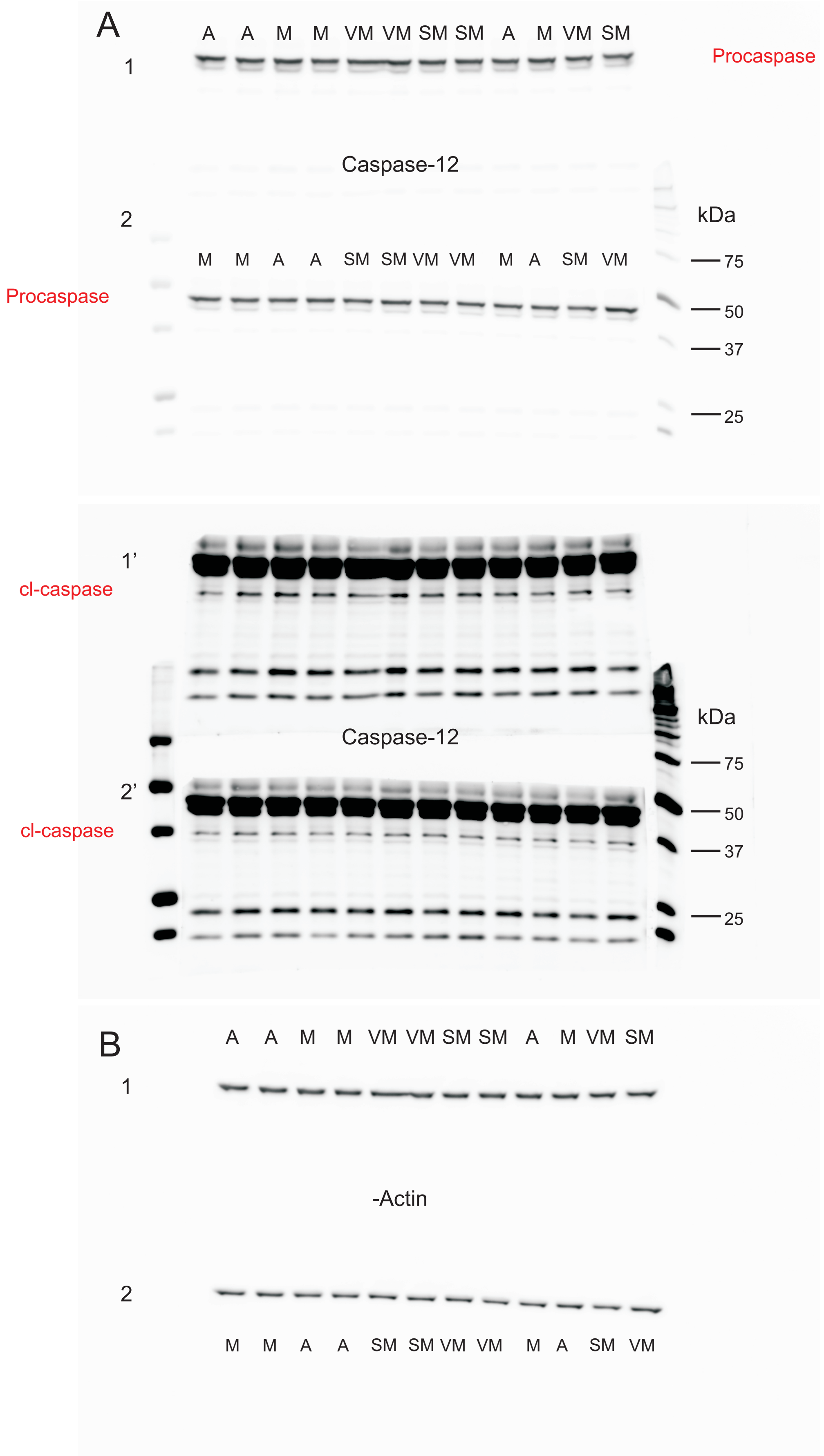

Fig. S27. Original blots presenting caspase-12 expression in preadolescent rats (A). After membrane stripping, blots 1 and 2 were reprobed with anti- -Actin antibody to control gel loading and transfer (B). The blots 1, 2 were exposed together, therefore they constitute one image. Lower image in A (1’, 2’) was subjected to a higher exposure time than upper image (the same blots) to evaluate cl-caspase levels. Molecular weight standards were matched only with blot 2. Red arrows indicate the bands subjected to the analysis. *Abbreviations:* A (AFR), animal facility rearing; cl, cleaved; M (MS), maternal separation; VM (VEH-MS); SM (SAL-MS); SAL, salubrinal; VEH, vehicle.

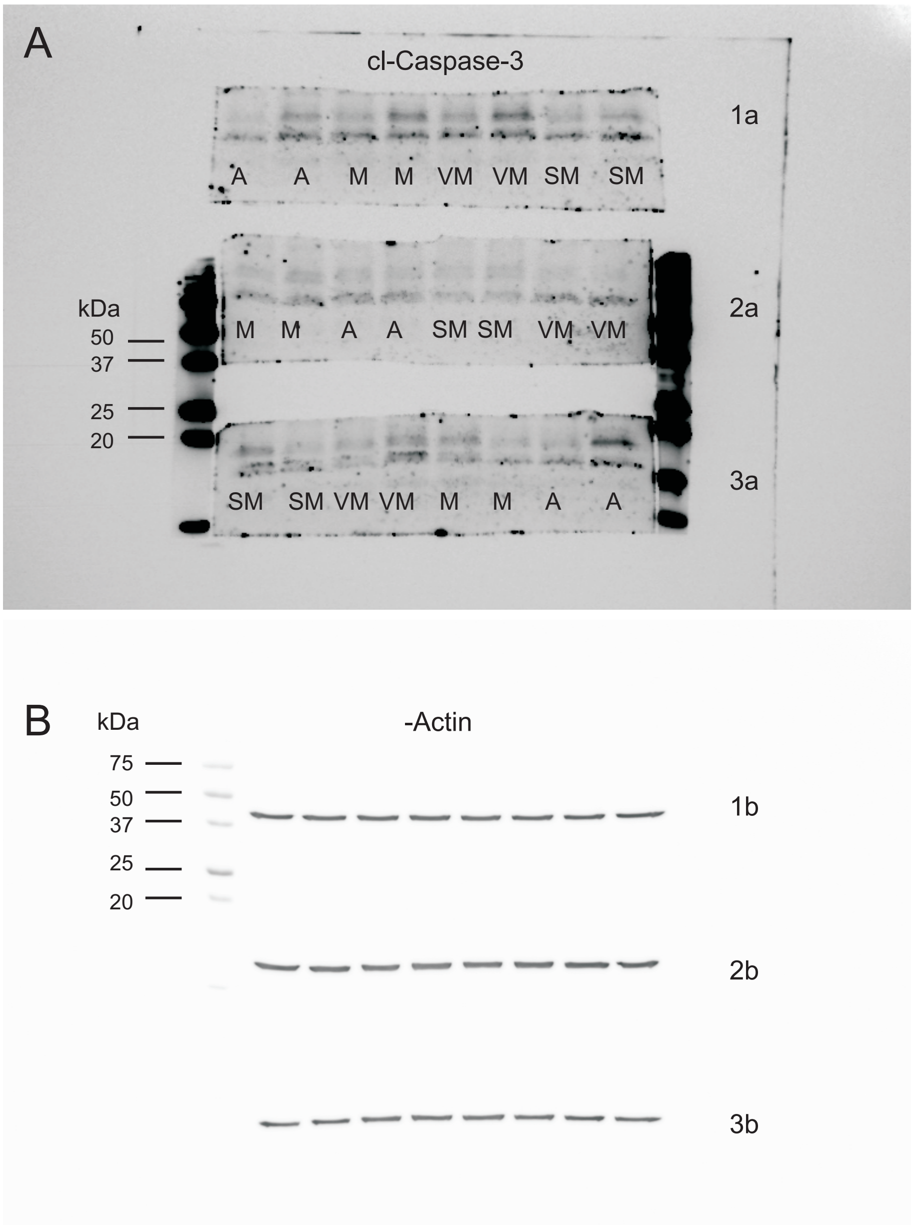

Fig. S28. Original blots presenting cl-caspase-3 expression in preadolescent rats (A) and -Actin immunoreactivity as control of gel loading and transfer (B). After a transfer, three distinct blots (1, 2, 3) were cut into pieces (a and b) at the level of 25 kDa to separately evaluate cl-caspase-3 (1a, 2a, 3a) and -Actin (1b, 2b, 3b). The blots 1a, 2a, 3a and 1b, 2b, 3b were exposed together, therefore they constitute one image. Red arrows indicate the band subjected to the analysis. Molecular weight standards were matched only with blot 3a and 1b, respectivelly. *Abbreviations:* A (AFR), animal facility rearing; cl, cleaved; M (MS), maternal separation; VM (VEH-MS); SM (SAL-MS); SAL, salubrinal; VEH, vehicle.
